# Disordered–Ordered Protein Binary Classification by Circular Dichroism Spectroscopy

**DOI:** 10.3389/fmolb.2022.863141

**Published:** 2022-05-03

**Authors:** András Micsonai, Éva Moussong, Nikoletta Murvai, Ágnes Tantos, Orsolya Tőke, Matthieu Réfrégiers, Frank Wien, József Kardos

**Affiliations:** ^1^ ELTE NAP Neuroimmunology Research Group, Department of Biochemistry, Institute of Biology, ELTE Eötvös Loránd University, Budapest, Hungary; ^2^ Department of Biochemistry, Institute of Biology, ELTE Eötvös Loránd University, Budapest, Hungary; ^3^ Institute of Enzymology, Research Centre for Natural Sciences, Budapest, Hungary; ^4^ Laboratory for NMR Spectroscopy, Research Centre for Natural Sciences, Budapest, Hungary; ^5^ Synchrotron SOLEIL, Gif-sur-Yvette, France; ^6^ Centre de Biophysique Moléculaire, CNRS UPR4301, Orléans, France

**Keywords:** intrinsically disordered proteins, CD spectroscopy, protein secondary structure, disorder identifier, disorder–order classification, machine learning

## Abstract

Intrinsically disordered proteins lack a stable tertiary structure and form dynamic conformational ensembles due to their characteristic physicochemical properties and amino acid composition. They are abundant in nature and responsible for a large variety of cellular functions. While numerous bioinformatics tools have been developed for *in silico* disorder prediction in the last decades, there is a need for experimental methods to verify the disordered state. CD spectroscopy is widely used for protein secondary structure analysis. It is usable in a wide concentration range under various buffer conditions. Even without providing high-resolution information, it is especially useful when NMR, X-ray, or other techniques are problematic or one simply needs a fast technique to verify the structure of proteins. Here, we propose an automatized binary disorder–order classification method by analyzing far-UV CD spectroscopy data. The method needs CD data at only three wavelength points, making high-throughput data collection possible. The mathematical analysis applies the *k*-nearest neighbor algorithm with cosine distance function, which is independent of the spectral amplitude and thus free of concentration determination errors. Moreover, the method can be used even for strong absorbing samples, such as the case of crowded environmental conditions, if the spectrum can be recorded down to the wavelength of 212 nm. We believe the classification method will be useful in identifying disorder and will also facilitate the growth of experimental data in IDP databases. The method is implemented on a webserver and freely available for academic users.

## Introduction

Intrinsically disordered proteins (IDPs) or protein regions (IDRs) lack a stable tertiary structure and form dynamic conformational ensembles (Dunker et al., 2002; Habchi et al., 2014). They are abundant in nature, especially in eukaryotes, and responsible for a plethora of cellular functions (Peng et al., 2015). Overall, 3–17% of eukaryotic proteins are estimated to be fully disordered (Dunker et al., 2000), and 30–50% of proteins contain IDRs (Dunker et al., 2000; Ward et al., 2004). The recently published state-of-the-art structure prediction method, AlphaFold2, provides confident prediction for only 58% of the residues on nearly the entire human proteome (Tunyasuvunakool et al., 2021), indicating that more than 40% of the residues fall into regions with significant structural flexibility. The biological importance of disordered proteins is underlined by the fact that malfunction of IDPs can lead to a variety of diseases (Uversky et al., 2008; Ruan et al., 2019). Given that IDPs have fundamentally different physicochemical properties than globular proteins, identifying disordered proteins and regions based on the amino acid sequence is highly desirable. In the last decade, dozens of bioinformatics tools have been developed to predict intrinsic disorder and its molecular function (Varadi et al., 2015; Katuwawala et al., 2020). Although these tools provide fast and high-throughput analysis, they have a substantial error rate and the actual predictions need experimental verification. The main experimental techniques applied to investigate intrinsic disorder include NMR, X-ray, circular dichroism (CD) spectroscopy, cryo-EM, and other spectroscopic techniques and techniques that study the hydrodynamic radius or surface exposure. Despite significant efforts to characterize structural disorder in detail, our knowledge remains limited. Even DisProt, the largest database of manually curated, experimentally verified disordered proteins and regions (Quaglia et al., 2021), only contains annotations of around 2000 proteins covering a small fraction of the predicted amount. Most of the structure characterization methods have high time and sample requirements; hence, there is a high need for fast, high-throughput, and inexpensive experimental methods to verify disorder.

CD spectroscopy has been widely used to study the structure of proteins. Near-UV CD spectra in the 250–300 nm wavelength range are determined by the aromatic side chains and their environment. In disordered conformation, these side chains are accessible for the polar solvent, and their environment is averaged out resulting in a nearly zero CD signal. Therefore, such a low signal could be the sign of disorder; however, IDPs usually contain a low number of aromatic residues, which restricts the practical use of this method. Moreover, near-UV CD needs a relatively large amount of sample because of the long path length and high required protein concentration (Woody and Berova, 2000). Far-UV CD spectra are characteristic of the secondary structure of proteins and need two orders of magnitude less amount of protein for the measurements than near-UV measurements. Disordered proteins exhibit characteristic CD spectra with an intensive minimum in the vicinity of 200 nm and a low amplitude around 222 nm (Adler et al., 1973; Provencher and Gloeckner, 1981; Johnson, 1988; Kelly and Price, 1997; Uversky, 1999). Uversky and co-workers reported that by using these two wavelengths for a double plot, it is possible to distinguish intrinsically disordered proteins from the ones with high secondary structure contents, such as molten globules and native globular proteins (Uversky, 2002; Uversky, 2003; Uversky and Fink, 2004). Our aim in the present work was to revise this observation and work out an automatized method for improved identification of IDPs by CD spectroscopy. We collected a larger reference set of CD spectra of ordered globular proteins and disordered polypeptide chains based on our own measurements, data downloaded from the protein CD database (PCDDB) (Whitmore et al., 2017), and collected from the literature. Starting with the double-wavelength plot, we applied various algorithms searching for an optimal method to identify disordered proteins from the spectral information gathered by CD spectroscopy. We examined the number and values of wavelengths needed for accurate disorder detection. To find the optimal method, the robustness regarding the sensitivity for incorrect concentration determination and experimental noise were also taken into account. Based on our findings, we provide a thorough comparison of the various analysis methods and propose an optimal protocol for IDP detection.

## Materials and Methods

### CD Spectroscopy

Synchrotron radiation CD (SRCD) spectra were recorded at the DISCO beamline of SOLEIL French synchrotron facility (proposal Nos. 20181890, 20191810, and 20200751). Samples at 5–7 mg/ml were measured in CaF_2_ cells with path lengths of 6–20 μm. In total, 6–12 scans were accumulated in the 175–270 nm or 180–270 nm wavelength range depending on the sample absorption; 1 nm data steps with a lock-in time constant of 300 ms and integration time of 1,200 ms were used. After baseline subtraction, the spectrum was corrected with the CSA calibration (Chen and Yang, 1977). Protein concentration was determined by directly measuring the absorbance of the CD sample and buffer reference at 205 and 214 nm (Kuipers and Gruppen, 2007; Anthis and Clore, 2013). For the case studies, CD experiments were carried out on a Jasco J-810 spectropolarimeter (Japan Spectroscopic Co., Tokyo, Japan). Protein concentrations 10, 1, and 0.1 mg/ml were used with quartz cells of 13 μm, 103 μm, and 1 mm path lengths, respectively.

### Mathematical Models Used for Disordered–Ordered Binary Classification

For disordered–ordered classification, the following built-in models of the MATLAB Classification Toolbox were used.

*Tree:* A binary classification decision tree is a learning method, where internal nodes represent the inspection of a predictor, branches show the outcome of the inspection, and leaf nodes represent class labels. Based on the number of leaves, we categorized trees as “simple” and “medium.” The maximum number of leaves is 4 in a simple tree and 20 in a medium tree.

*Support vector machines:* SVMs are methods which use a subset of training data to create a decision function. The data points in this subset are called support vectors. We used different kernel functions for our models: linear and radial basis function (RBF). SVM algorithms aim to find a hyperplane that separates two labeled classes with the widest possible margin.

*K-nearest neighbors:* KNN classification is based on finding the k-nearest training point to the new data point and using them to predict the label. We used four types of KNN methods which calculate the Euclidean distance between data points. “Fine,” “medium,” and “coarse” examine 1, 10, and 100 nearest neighbors, respectively. “Weighted” applies a squared inverse distance weighting function on the 10 nearest neighbors, which results in nearer neighbors having a larger impact. The fifth KNN method we implemented uses a different distance metric; it considers the cosine of the angle between vectors pointing from the origin to data points searching for 10 nearest neighbors.

*Discriminant:* Discriminant analyses create a decision surface, which may be linear or quadratic. In the case of “diaglinear” and “diagquadratic” models, the covariance matrices are diagonal (i.e., all the off-diagonal elements—covariances—are zeros; only variances are non-zero values). As opposed to SVM models, discriminant analyses do not include the condition of making margins as wide as possible.

### Steps of Finding the Optimal Classification Method

We aimed to classify proteins based on two or three data points. Therefore, we implemented classifiers that consider either a pair or a triplet of wavelengths, and perform classification by using CD values at the given wavelengths. Wavelength pairs and triplets consisted of wavelengths with a minimum pairwise difference of 3 nm in the 175–250 nm wavelength range. To develop the disorder determination method in a certain wavelength range, we used all proteins whose spectra covered the studied range.

Leave-one-out cross-validation error rates were calculated by summing misclassified proteins and dividing their number by the total number of proteins. Error rates were determined separately for disordered and ordered proteins and for the total dataset.

The robustness of each method was also tested. We simulated the effect of inaccurate concentration measurement by rescaling the amplitude of test spectra and examined the sensitivity of methods to the scaling factor in the range of 0.5–2. Furthermore, the dependence of the methods’ accuracy on noise was evaluated. Noise was added independently to each CD value using random values from normal distribution (µ = 0 M^−1^ cm^−1^, σ = 0.1 M^−1^ cm^−1^ or σ = 0.05 M^−1^ cm^−1^). The effect of noise was calculated by averaging the results of 1,000 simulations.

When picking the best classifiers, the global error, error on disordered structure, preferably higher wavelengths for analysis, and the robustness were considered.

MATLAB scripts used in the present study are provided in the Supplementary Material.

## Results and Discussion

### Reference Dataset of IDPs and Ordered Proteins

To investigate the problem of distinction between disordered and ordered protein structures based on CD data alone, we collected the CD spectra of IDPs and proteins with ordered structures from various sources. In total, 140 high-quality SRCD spectra in a wide wavelength range from 175 or 180 nm of globular native proteins were downloaded from the protein CD databank (PCDDB) (Whitmore et al., 2017). The spectra of 9 globular native proteins, 2 amyloid fibrils, and 26 disordered polypeptides were the result of our SRCD measurements. These include IDPs, such as ERD14 (early responsive to dehydration) plant chaperone and its variants (Murvai et al., 2021), histone–lysine N-methyltransferase constructs, artificial peptides designed for maximal disorder, and β-structure-rich globular proteins, such as dUTPase and SH3 domains that have CD spectra similar to disordered proteins. Overall, 85 spectra were collected from the literature (based on the references in Uversky (2002), Uversky (2003), Uversky and Fink (2004)), including those of 30 globular proteins and 55 IDPs. These spectra varied in their wavelength range. To develop the disorder prediction method in a certain wavelength range, we used all proteins whose spectra covered the studied range. The proteins of the reference set are presented in Supplementary Table S1, and the size of the reference set as a function of the wavelength cutoff is presented in Supplementary Figure S1.

### Classical CD Plot of IDPs and Ordered Proteins

**FIGURE 1:**
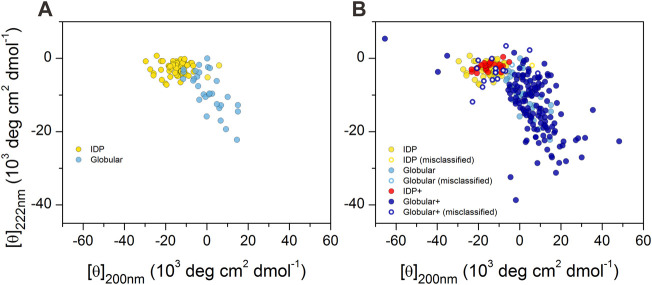
2D-plot of CD data of IDPs and ordered proteins. **(A)** Mean residue ellipticities at 200 and 222 nm wavelengths for IDPs (yellow) and globular proteins (light blue) were collected from the literature for proteins previously studied by Uversky (2002), Uversky (2003), Uversky and Fink (2004). “Random coil” and “premolten globule” types of IDPs were not distinguished in our work. **(B)** Plot of the full reference database. IDPs over the ones presented in **(A)** are shown in red, while the additional globular ones are shown in dark blue. Hollow circles show those proteins that are incorrectly classified as disordered or ordered by using the 200 and 222 nm wavelength data of proteins presented in panel A as training set for disordered–ordered classification (see later). Note the large spectral (and conformational) space covered by the ordered proteins.

We reproduced the double-wavelength plot using CD intensities at 200 and 222 nm wavelengths on the available data on proteins reported by Uversky (2002), Uversky (2003), Uversky and Fink (2004), as shown in Figure 1A. IDPs and globular proteins were separated with some overlap in the plot. However, when we completed this plot with all the proteins in our database, this picture has changed significantly (Figure 1B). Although the newly added spectra of disordered peptides concentrated well on the previous disordered ones, the globular proteins covered a much wider space and even overlapped with the disordered region ruining the spatial separation.

**FIGURE 2:**
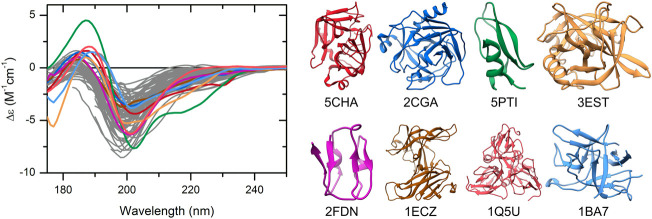
CD spectra of disordered proteins and some globular proteins with similar spectra. Proteins rich in highly twisted antiparallel β-sheets (colored spectra and corresponding structures) exhibit CD spectra reminiscent of disordered proteins (gray), which makes the distinction between them difficult. Alpha-chymotrypsin (PDB ID: 5CHA), chymotrypsinogen (2CGA), trypsin inhibitor (5PTI), elastase (3EST), ferredoxin (2FDN), ecotin (1ECZ), dUTP pyrophosphatase (1Q5U), and trypsin inhibitor (Kunitz) (1BA7) are shown.

The CD spectra of those globular proteins that are located in the disordered region in the double-wavelength plot are similar to that of the disordered ones, despite their fully ordered globular structure (Figure 2). Their X-ray structures revealed that these proteins have highly right-hand twisted antiparallel β-sheet structures (Ho and Curmi, 2002; Micsonai et al., 2015) (Figure 2). This problem has already been pointed out in our previous work (Micsonai et al., 2018) as a major issue in the distinction between highly twisted antiparallel β-sheets and disordered structures in secondary structure content estimation. These results reveal that the simple use of the 200 and 222 nm CD data might be insufficient for the proper distinction between IDPs and ordered proteins, and an improvement of this methodology is highly beneficial.

### Identification of IDPs Using Various Mathematical Models

To develop a binary classification method (IDP vs. ordered structure) for an accurate and automatized IDP identification, we analyzed the CD spectra of our database using various mathematical models, such as decision trees with different number of branches (tree: simple and medium); support vector machines with different kernel functions [SVM: linear and radial basis function (RBF)]; *k*-nearest neighbor classification with Euclidean distance and three different numbers of nearest neighbors, a weighted distance function and a cosine distance metric (KNN: fine, medium, coarse, weighted, and cosine); and discriminant analyses with linear or quadratic decision surface including linear diagonal or quadratic diagonal models (discriminant: linear, quadratic, diaglinear, and diagquadratic). These models are available in the MATLAB Classification Toolbox.

As a starting point, we tested the performance of using the CD amplitudes at 200 and 222 nm wavelengths to identify IDPs using the 85 spectra collected from the literature based on Uversky’s works (Uversky, 2002; Uversky, 2003; Uversky and Fink, 2004) as training set and using our entire database as test set (in a cross-validated manner). SVM–RBF was proven to be the best mathematical model providing 11.1, 3.5, and 8.6% errors in identifying the ordered structures, disordered structures, in overall accuracy, respectively (see also Figure 1B). In the next step, we tested the performance of all models using CD data at two wavelengths varying the wavelength values to find the best performing pairs as a function of the cutoff wavelength of the CD spectra. The different methods varied in global error and in the error on ordered and disordered structures. We selected the best methods for minimal global errors and for minimal errors in disorder prediction. The results were dependent on the spectral range (wavelength cutoff), as shown in Supplementary Table S2. Generally, decision tree algorithms provided good performance; however, other models also gave similar results. The error was increasing with higher cutoff wavelengths. As an example, with 200 nm cutoff, SVM-linear showed 7.7 and 2.5% errors for ordered and disordered structures and 6.1% global error using the 204 and 215 nm wavelength pair, respectively.

On further analysis, we studied if disorder–order classification can be improved by using three data points. Spectra with 175 nm cutoff could be classified without any error by the SVM–RBF algorithm using the “182-194–209 nm” data triplet (Table 1). It is worthy to note that the number of disordered spectra was only 21 in this wavelength range. For all algorithms, the error was increasing with higher wavelength cutoff; however, it was significantly lower in the case using two wavelengths for classification. At each cutoff wavelengths, 3–5 algorithms gave similar results, making it difficult to select between them at first sight. Generally, SVM-linear and RBF, KNN-fine and cosine, tree-medium, and discriminant-quadratic algorithms using various wavelength triplets worked efficiently. At 200 nm cutoff, the accuracy is decreased, which, we believe, is because the spectra collected down to 175 or 180 nm have higher quality than the spectra collected from the literature with 190 or 200 nm wavelength cutoffs. Spectra in the PCDDB and collected by us underwent a careful inspection (Woollett et al., 2013). However, the error of the classification is still sufficiently low for these methods to be suitable as experimental classifiers for IDPs (Table 1). The error of classification for all the algorithms as the function of cutoff wavelength for two and three wavelengths is presented in Supplementary Figures S2–S5. Tables presenting the detailed results of all algorithms are provided as the Supplementary Material.

**TABLE 1:** Disorder–order classification using three wavelengths.^a^

	Wavelength (nm)				Error (%)		
Cutoff (nm)	Algorithm	WL1	WL2	WL3	Ordered	Disordered	Global
175	SVM–RBF	182	194	209	0	0	0
	Discr-quadratic	179	214	225	0.8	0	0.7
	Tree-medium	192	220	228	0.8	0	0.7
180	KNN-fine	184	197	208	0.7	0	0.6
	Discr-quadratic	197	216	221	1.3	0	1.1
	SVM–RBF	195	217	227	2	0	1.7
	Tree-simple	185	192	211	2	0	1.7
185	Tree-medium	191	201	250	1.3	2.7	1.6
	SVM–RBF	195	217	227	2	2.4	2.1
	Discr-quadratic	199	213	234	2	2.4	2.1
190	Tree-medium	191	201	250	1.9	5.6	2.8
	SVM–RBF	196	216	229	2.4	5.1	3.1
	Discr-quadratic	199	213	234	3.5	1.7	3.1
195	Discr-quadratic	199	213	234	3.5	2.9	3.3
	SVM-linear	196	212	235	4.1	1.5	3.4
	KNN-cosine	197	206	233	4.7	1.5	3.8
	SVM–RBF	196	216	223	3.5	4.4	3.8
	Discr-linear	195	219	237	3.5	4.5	3.8
200	KNN-cosine	212	217	225	4.7	1.5	3.8
	SVM-linear	202	205	231	7.2	2.5	5.7
	SVM–RBF	206	212	229	5	7.5	5.7
	Discr-quadratic	201	211	215	6.6	3.8	5.7
	KNN-fine	212	215	227	3.9	10	5.7
205	KNN-cosine	212	217	225	3.3	7.4	4.6
	SVM–RBF	206	212	229	5	7.4	5.7
	KNN-fine	212	215	227	3.9	9.9	5.7

a Algorithms showing the least errors using three wavelengths (WL1, WL2, WL3) for classification as a function of the cutoff wavelength are presented. For training dataset, for a given wavelength triplet, all proteins’ spectra that covered those wavelengths were used.

### Effect of Concentration Error on Disorder–Order Classification

**FIGURE 3:**
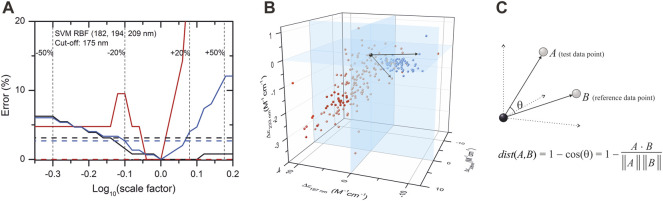
Effect of concentration error on disordered–ordered classification and introduction of the KNN-cosine method. **(A)** Error of the SVM–RBF algorithm as a function of the scaling factor on the spectra of the database with 175 nm cutoff are shown for disordered (red) and ordered (black) structures. The global error is shown in blue. Dashed lines show the errors of classification using the KNN-cosine algorithm for disordered (red), ordered (black), and the overall error (blue). For convenience, ±20% and ±50% changes in the concentration (i.e., in the scaling factor) are shown. **(B)** Reference points in the space determined by the CD data measured at 197, 206, and 233 nm wavelengths and an example for vectors by using the KNN-cosine method. Red and blue points represent ordered and disordered proteins, respectively. **(C)** The distance metric of this KNN algorithm uses the cosine of the angle between vectors pointing from the origin to data points. The prediction is based on the labels (ordered/disordered) of the first 10 reference points with the lowest “distance” from the test point. The direction and the angles of the vectors will not change with scaling, that is, the method is independent of concentration errors.

Due to their unusual amino acid composition, concentration determination of IDPs with the widely used basic techniques is challenging and might lead to large inaccuracies (Szőllősi et al., 2007). Measurement by the aromatic absorption is problematic because of the usually low number of such residues in IDPs. Colorimetric assays are also affected by the special amino acid composition of IDPs and are sensitive to contaminations. One solution might be the absorbance measurement at 205 or 214 nm (Kuipers and Gruppen, 2007; Anthis and Clore, 2013; Micsonai et al., 2021); however, buffer absorption can limit its applicability. Measurement by mass of the dry sample usually also produces errors because of the bound water or remaining salts. We estimated that a 20% error might regularly occur in concentration measurements of IDPs, which might have an effect on the accuracy of disorder classification. Thus, we tested the robustness of the classification algorithms for such errors by re-evaluating the spectra after rescaling them with factors between 0.5 and 2. Supplementary Figure S6 shows the dependence of the classification error on the rescaling for the various algorithms presented in Table 1. Most of the methods showed a surprisingly high sensitivity for concentration errors. The SVM–RBF algorithm works without error on the correctly normalized spectra (scale factor = 1); however, even a 10% increase in the spectral amplitude increases the error on the disordered structure identification to over 10% (Figure 3A). The exception is the KNN-cosine method, which showed no dependence on the spectral amplitude (Figure 3A). The “cosine” distance metric of the KNN algorithm uses the cosine of the angle between vectors pointing from the origin and data points. The direction of these vectors will neither change with scaling nor will the angles. Considering these facts, we propose the selection of KNN-cosine as the optimal classification algorithm. It performs with acceptable accuracy and is free of concentration errors (Figures 3B,C). Table 2 shows the performance of KNN-cosine as a function of the wavelength cutoff. Intriguingly, the best wavelength triplet in the cutoff range from 175 to 179 nm is proven to be the “214-218–232 nm” triplet. It suggests that we do not really need CD data down to 175 nm for the binary classification. However, with a cutoff of 200 nm, KNN-cosine provided significantly lower accuracy, despite the fact that the lower wavelength range was not needed for the method. We believe this is because of the quality difference between SRCD spectra collected down to 175 nm and conventional measurements with 200 nm wavelength minimum. To address this question, further investigations were carried out.

**TABLE 2:** Accuracy of KNN-cosine algorithm as a function of cutoff wavelength.^a^

	Wavelength (nm)			Error (%)		
Cutoff (nm)	WL1	WL2	WL3	Ordered	Disordered	Global
175	214	218	232	1.6	0	1.3
176	214	218	232	1.5	0	1.3
177	214	218	232	1.5	0	1.3
178	214	218	232	1.5	0	1.3
179	214	218	232	1.5	0	1.3
180	197	206	233	4	0	3.4
183	197	206	233	4	0	3.3
185	197	206	233	3.9	0	3.1
190	197	206	233	4.7	1.7	3.9
195	197	206	233	4.7	1.5	3.8
198	198	205	237	4	2.9	3.7
200	212	217	225	3.3	7.5	4.6
205	212	217	225	3.3	7.4	4.6

a Wavelengths of the data points (WL1, WL2, WL3) for the best performance at each cutoff and the errors of classification are shown.

### Effect of Experimental Noise

**FIGURE 4:**
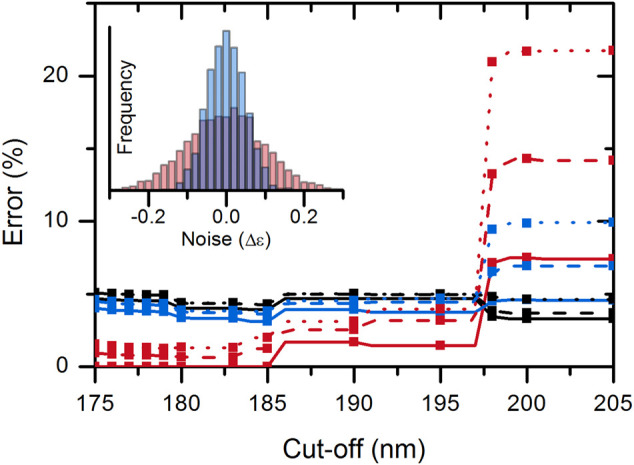
Accuracy of the KNN-cosine method as a function of wavelength cutoff. Error on disordered (red) and ordered (black) proteins and the global error (blue) are shown with solid curves for the original spectra and with dashed and dotted lines for spectra with added noise of σ = 0.05 and 0.1 M^−1^cm^−1^, respectively. Up to 197 nm cutoff, the “197-206-233 nm” triplet and above 197 nm, the “212-217-225 nm” triplet were used for analysis.

To investigate the effect of spectrum quality/spectral noise on the disordered–ordered classification, we added artificial noise to the spectra tested. The noise was added independently to each CD value using random values from normal distribution (µ = 0 M^−1^ cm^−1^, σ = 0.1 M^−1^ cm^−1^ or σ = 0.05 M^−1^ cm^−1^). The effect of noise was calculated by averaging the results of 1,000 simulations on each of the wavelength triplets of the KNN-cosine model on the possible wavelength cutoff ranges (Supplementary Figure S7). The addition of noise significantly increased the error of classification. Noise had the highest effect when using the “214-218-232 nm” data triplet possibly because 214 and 218 nm data are close to each other. The “197-206-233 nm” triplet was more robust for noise and generally showed a good performance for all possible wavelength ranges from 175 nm up to 197 nm cutoffs. Therefore, we suggest using this model as a classification tool. Above 197 nm, the “212-217-225 nm” data triplet should be used (Supplementary Figure S7). Performance of the KNN-cosine method combined for all wavelength cutoffs including the effect of noise is presented in Figure 4.

Based on all these results, for the best disorder–order classification, it is recommended to collect good-quality CD spectra down to ∼195 nm and use the KNN-cosine algorithm with data at 197-206-233 nm wavelengths.

### Disorder Classification for Limited Wavelength Range, Under Strong Absorbing Conditions

It is an interesting and maybe unexpected finding that KNN-cosine with the data triplet “212-217-225 nm,” that is, with 212 nm lowest wavelength, is a good choice for disorder classification. Although the error shown in Figure 4 is increased for cutoffs above 197 nm, this is somewhat misleading. This method gives better results for high-quality spectra recorded down to 175–180 nm even without using any of their data points below 212 nm for the classification (Supplementary Figure S7). The error on these spectra, downloaded from PCDDB or measured by us using SRCD, is 3.3, 0, and 2.8% for ordered structure, disordered structure, and globally, respectively. These spectra were treated and validated using careful protocols (Kelly et al., 2005; Woollett et al., 2013; Micsonai et al., 2021). The 89 spectra collected from the literature have obviously lower average quality, and this increases the error of the classification on them to 3.3, 10.9, and 8.24% for disordered and ordered structures and for global error, respectively. These calculations were performed in a leave-one-out cross-validated manner using all available data as training dataset. Careful, noiseless experiments with correct baseline subtractions might give better accuracy than the average error found here.

A real advantage of the KNN-cosine method with “212-217-225 nm” data is that it can be used for CD spectra recorded in the presence of strongly absorbing solutions such as the case of chemical denaturants (e.g., urea and GdnHCl), or under crowded conditions if the spectrum can only be recorded down to ∼210 nm. It might help to study the crucial question if a supposedly IDP will indeed exhibit disordered structures under crowded conditions or become structured (Szasz et al., 2011; Qin and Zhou, 2013; Banks et al., 2018; Simpson et al., 2020; König et al., 2021).

### Experimental Classification of Disorder vs. *In Silico* Predictions

Numerous bioinformatics tools have been developed in the last decade to predict intrinsic disorder from the amino acid sequence (Katuwawala et al., 2019; Liu et al., 2019; Necci et al., 2021). Among them, AlphaFold2 was proven to be the most accurate method to detect disorder. Low values of the plDDT parameter (confidence) have been shown to be indicative of disordered regions (Jumper et al., 2021)*.* AlphaFold2 and previous methods are useful when investigating large datasets, and high-throughput analysis is needed, and they indeed provide good statistics. However, *in silico* predictions always have a level of uncertainty and thus need experimental verification, especially when investigations are narrowed down and focus on a particular protein. To confirm this statement, we analyzed the disordered proteins of our reference database by AlphaFold2 and found that several disordered chains were mistakenly predicted to be highly α-helical, such as α-synuclein, thymosin-α1, basic subdomain of the c-Jun oncoprotein, α-tubulin (fragment 404–451), β-tubulin (fragment 395–445), S21 protein from the 30S subunit of the *E. coli* ribosome, and artificial disordered peptides #1, 2, and 6. Moreover, computational methods, like AlphaFold2, can neither take the actual environmental conditions into account, such as pH, ionic strength, temperature, the presence of additives or crowding agents, the effect of protein concentration, intermolecular interactions, nor accurately calculate the effect of single mutations (unless the crystal structure was already solved and deposited in the PDB) and the effects of post-translational modifications (e.g., phosphorylation) (Pak et al., 2021; Perrakis and Sixma, 2021). As IDPs are specifically sensitive to their surroundings, depending on the solvent environment, a single polypeptide chain can take up various conformations, which results in important biological readouts. Therefore, an experimental method, such as CD spectroscopy, can validate and specify the prediction of AlphaFold2 and should be used for this purpose. When CD spectroscopy confirms the prediction of AlphaFold2, the site-specific information of AlphaFold2 is likely valuable. However, if there is a large discrepancy between the prediction of AlphaFold2 and the experimental results, then the priority has to be given to the experience.

### Case Studies

**FIGURE 5:**
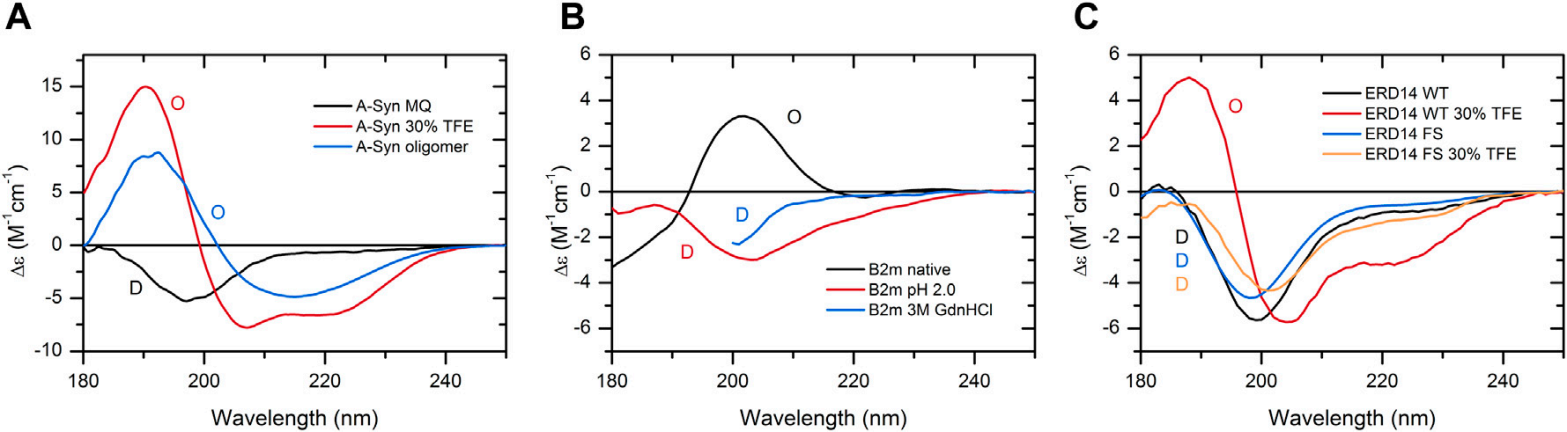
Case studies showing the structural variability of individual proteins, which can only be revealed experimentally. **(A)** CD spectrum of α-synuclein in water is characteristic of a fully disordered chain. In 30% TFE, the protein exhibits an ordered, α-helix-rich conformation, and at higher concentrations (10 mg/ml), it readily forms oligomers with a spectral shape of β-structure. **(B)** In the native state, β_2_-microglobulin (β2m) exhibits a β-sandwich fold of an antiparallel β-structure. At low pH or in 3 M GdnHCl, its structure becomes disordered. **(C)** ERD14 disordered plant chaperone and its artificial pair consisting of the full scrambled sequence both exhibit disordered structure in water. The presence of 30% TFE induces the formation of α-helix in the wild-type protein, while its scrambled variant preserves its disordered conformation. For **(C)**, experimental data modified from Murvai et al. (2021) were used with the authors’ permission. The results of the binary classification are shown by O (ordered) and D (disordered) letters in the figures.

As a further support for the aforementioned statement, here, we provide specific case studies presenting the dependence of the protein structure and disorder on the buffer conditions. These reveal the necessity of experimental techniques and the limitations of *in silico* predictions for the detection of protein disorder. One example is the well-known α-synuclein, a protein associated with Parkinson’s disease. It is an IDP, and CD spectroscopy shows that indeed, the protein is disordered under physiological buffer conditions. In the presence of 30% TFE, which mimics a less polar solvent environment, such as in membranes, the protein becomes ordered with 47% α-helix content as estimated from the CD spectrum by the BeStSel algorithm (Micsonai et al., 2015; Micsonai et al., 2018). At concentrations above 2 mg/ml, α-synuclein readily forms oligomers in 30% TFE with a spectral shape characteristic of the β-structure. The corresponding CD spectra of α-synuclein and results of the binary classification are shown in Figure 5A. In contrast, AlphaFold2, irrespectively of the buffer conditions, erroneously predicts with high confidence that 64% of the α-synuclein chain is in an α-helical structure.

β2-microglobulin (β2m) is the light chain of MHC-1 and can also be found in a monomeric form in the blood. It causes serious complications upon long-term dialysis depositing in the form of amyloid fibrils in the osteoarticular system of patients. The native protein exhibits an immunoglobulin fold with an antiparallel β-sandwich, which is a cinch for AlphaFold2. However, β2m is sensitive to the drop of pH; it becomes unfolded below pH 4, which cannot be deduced from the AlphaFold2 prediction. We also present the disordered spectrum of the protein in 3 M GdnHCl, showing that it is possible to identify disordered structures even in highly absorbing solutions by our method (Figure 5B).

ERD14 is a disordered plant chaperone, which is correctly predicted by AlphaFold2. However, in 30% TFE, the protein gains a significant amount of α-helix, which turns out to be indispensable for the protein’s function (Murvai et al., 2020). An artificial variant of ERD14 with a full-scrambled sequence (having the same amino acid composition) and no biological function is similarly disordered in water; however, in the presence of TFE, it still preserves its disordered conformation. In this type of comparison, CD spectroscopy reveals the secondary structure forming tendency of disordered wild-type ERD14 under suitable conditions or upon intermolecular interactions (Figure 5C).

In our previous work on a Trp-cage miniprotein (Kardos et al., 2015), we showed that a single side-chain phosphorylation can cause drastic conformational changes. Our classification shows that the protein obviously loses its α-helix content and becomes disordered upon the phosphorylation of its Ser9 residue. Such drastic change is also missed when the structure is predicted with AlphaFold2.

These examples reveal the limitations of *in silico* predictions and the necessity of integration of various experimental techniques for the detection of protein disorder.

### Limitations: Intrinsically Disordered Regions (IDRs)

The binary classification method presented here is to identify essentially disordered proteins, that is, to detect “global” disorder. In the case of partial disorder, this binary classification will not detect a disordered protein region of an otherwise ordered protein. In such a case, partial disorder can be deduced from the secondary structure composition determined by analyzing the entire CD spectrum with some of the available methods, such as BeStSel (Micsonai et al., 2018; Micsonai et al., 2021). Upon intermolecular interactions of disordered proteins, localized segments might take up ordered structure, which, depending on the size of the segment, might not change the result of the classification. To study such partial structural changes, a full CD spectrum analysis is required with BeStSel (Micsonai et al., 2015; Micsonai et al., 2018) or other algorithms (Sreerama and Woody, 2000; Lobley et al., 2002).

## Conclusion

Intrinsically disordered proteins are abundant in nature and responsible for a plethora of cellular functions (Dunker et al., 2002; Habchi et al., 2014). They lack a stable tertiary structure and form dynamic conformational ensembles due to their characteristic physicochemical properties and amino acid composition (Varadi et al., 2015; Katuwawala et al., 2020). Although numerous bioinformatics tools have been developed for disorder prediction in the last 2 decades, there is still a high need for experimental verification of the disordered state. Here, we proposed an automatized binary disorder–order classification by analyzing far-UV CD spectroscopy data. The method uses CD data at three wavelength points, which makes high-throughput data collection possible. To reach the best classification accuracy, CD of the protein should be measurable down to 197 nm in good quality. However, in case of strong absorbing samples, such as in crowded environmental conditions, 212 nm lowest wavelength still provides acceptable performance. The mathematical analysis uses the *k*-nearest neighbor algorithm with cosine distance function, which is independent of the spectral amplitude, that is, free of concentration determination errors. We believe the classification method will be useful in identifying or verifying disorder in individual problems and will also facilitate the growth of experimental data in IDP databases, such as DisProt (Quaglia et al., 2021). The method is implemented on a webserver and freely available for academic use at https://bestsel.elte.hu/idp_classification.php.

## Data Availability

The datasets generated for this study are included in the article/Supplementary Material; further inquiries can be directed to the corresponding author.
