# Supplementary material for: Disordered–Ordered Protein Binary Classification by Circular Dichroism Spectroscopy
Source: Front Mol Biosci. 2022 May 3;9:863141. doi: 10.3389/fmolb.2022.863141 (PMC9110821; doi:10.3389/fmolb.2022.863141)
Supplement: Supplementary file 1 [file DataSheet2.PDF]

```
%--------------------------------------------------------------------------
--
% Matlab scipts for
% András Micsonai, Éva Moussong, Nikoletta Murvai, Ágnes Tantos, Orsolya
Tõke, Matthieu Réfrégiers, Frank Wien and József Kardos.
% Disordered-ordered protein binary classification by circular dichroism
spectroscopy.
% Frontiers in Molecular Biosciences, 2022
% Contact: kardos@elte.hu, micsonai@ttk.elte.hu
%--------------------------------------------------------------------------
--

% SVM2_Linear
%-------------
clear all
clc
load CD_from_PCDDB.mat;
load CD_from_Papers.mat;
load CD_from_Own_measurements.mat;
alldata_WL = [175:250]';
alldata_CD = [CD_PCDDB CD_Papers_ CD_Own];
alldata_Label = [Label_PCDDB Label_Papers_ Label_Own];
alldata_serial = [1:size(alldata_Label,2)];

alldata_minWL=zeros(1,size(alldata_CD,2));
for i=1:size(alldata_CD,2)
    alldata_minWL(1,i) = min(find(~isnan(alldata_CD(:,i))))+174;
end

Out_data = cell(1,15);
Out_data = {'WL1','WL2',...
            'Ordered reference',...
            'Unordered reference',...
            'Total reference',...
            'Cutoff',...
            'Ordered test',...
            'Unordered test',...
            'Total test',...
            'Ordered ERROR',...
            'Unordere ERROR',...
            'Total ERROR',...
            'Ordered ERROR Ratio',...
            'Unordered ERROR Ratio',...
            'Total ERROR Ratio'};
row = 1;
for wl1 = 175 : 250
    for wl2 = wl1+3 : 250
        tmp = find( ~isnan(alldata_CD(wl1-174,:)) &
~isnan(alldata_CD(wl2-174,:)) );
        reference_WL      = alldata_WL;
        reference_CD      = alldata_CD(:,tmp);
        reference_Label   = alldata_Label(:,tmp);
        reference_sorszam = alldata_sorszam(:,tmp);
        reference_minWL   = alldata_minWL(1,tmp);

        reference_preform = [reference_CD(wl1-174,:)' reference_CD(wl2-
174,:)' reference_Label'];
```

```
        pred=[];
        for CV = 1 : size(reference_CD,2)
            reference       = reference_preform;
            reference(CV,:) = [];
            Model           = fitcsvm(reference(:,1:end-
1),reference(:,end),'Standardize',false,'KernelFunction','linear');
            pred(CV,1)      = Model.predict(reference_preform(CV,1:end-
1));
        end

        unik_cutoff = unique(reference_minWL)';
        for cutoff = 1 : size(unik_cutoff,1)
            testset = find(reference_minWL <= unik_cutoff(cutoff));
            row = row+1;
            Out_data{row, 1} = wl1;
            Out_data{row, 2} = wl2;
            Out_data{row, 3} = size(find(reference_Label==0),2);
            Out_data{row, 4} = size(find(reference_Label==1),2);
            Out_data{row, 5} = size(reference_Label,2);
            Out_data{row, 6} = unik_cutoff(cutoff);
            Out_data{row, 7} = size(find(reference_Label(testset)==0),2);
            Out_data{row, 8} = size(find(reference_Label(testset)==1),2);
            Out_data{row, 9} = size(reference_Label(testset),2);
            Out_data{row,10} =
size(find( (pred(testset)~=reference_Label(testset)') &
(reference_Label(testset)==0)' ),1);
            Out_data{row,11} =
size(find( (pred(testset)~=reference_Label(testset)') &
(reference_Label(testset)==1)' ),1);
            Out_data{row,12} =
size(find(pred(testset)~=reference_Label(testset)'),1);
            Out_data{row,13} = Out_data{row,10} / Out_data{row,7};
            Out_data{row,14} = Out_data{row,11} / Out_data{row,8};
            Out_data{row,15} = Out_data{row,12} / Out_data{row,9};
        end
    end
end

All_Out_data = [];
Out_data_mat = cell2mat(Out_data(2:end,:));
unik_cutoff  = unique(Out_data_mat(:,6));
for cutoff = 1 : size(unik_cutoff,1)
    Out_data_cutoff =
Out_data_mat(find(Out_data_mat(:,6)==unik_cutoff(cutoff)),:);
    [u v]           = sort(Out_data_cutoff(:,end));
    All_Out_data    = [All_Out_data;
Out_data_cutoff(v(find(u==u(1))),:)];
end

SVM2_Linear = [Out_data(1,:); num2cell(All_Out_data)];
save SVM2_Linear.mat;

% SVM2_RBF
%----------
clear all
clc
load CD_from_PCDDB.mat;
load CD_from_Papers.mat;
```

```
load CD_from_Own_measurements.mat;
alldata_WL = [175:250]';
alldata_CD = [CD_PCDDB CD_Papers_ CD_Own];
alldata_Label = [Label_PCDDB Label_Papers_ Label_Own];
alldata_serial = [1:size(alldata_Label,2)];

alldata_minWL=zeros(1,size(alldata_CD,2));
for i=1:size(alldata_CD,2)
    alldata_minWL(1,i) = min(find(~isnan(alldata_CD(:,i))))+174;
end

Out_data = cell(1,15);
Out_data = {'WL1','WL2',...
            'Ordered reference',...
            'Unordered reference',...
            'Total reference',...
            'Cutoff',...
            'Ordered test',...
            'Unordered test',...
            'Total test',...
            'Ordered ERROR',...
            'Unordere ERROR',...
            'Total ERROR',...
            'Ordered ERROR Ratio',...
            'Unordered ERROR Ratio',...
            'Total ERROR Ratio'};
row = 1;
for wl1 = 175 : 250
    for wl2 = wl1+3 : 250
        tmp = find( ~isnan(alldata_CD(wl1-174,:)) &
~isnan(alldata_CD(wl2-174,:)) );
        reference_WL      = alldata_WL;
        reference_CD      = alldata_CD(:,tmp);
        reference_Label   = alldata_Label(:,tmp);
        reference_sorszam = alldata_sorszam(:,tmp);
        reference_minWL   = alldata_minWL(1,tmp);

        reference_preform = [reference_CD(wl1-174,:)' reference_CD(wl2-
174,:)' reference_Label'];

        pred=[];
        for CV = 1 : size(reference_CD,2)
            reference       = reference_preform;
            reference(CV,:) = [];
            Model           = fitcsvm(reference(:,1:end-
1),reference(:,end),'Standardize',false,'KernelFunction','rbf');
            pred(CV,1)      = Model.predict(reference_preform(CV,1:end-
1));
        end

        unik_cutoff = unique(reference_minWL)';
        for cutoff = 1 : size(unik_cutoff,1)
            testset = find(reference_minWL <= unik_cutoff(cutoff));
            row = row+1;
            Out_data{row, 1} = wl1;
            Out_data{row, 2} = wl2;
            Out_data{row, 3} = size(find(reference_Label==0),2);
            Out_data{row, 4} = size(find(reference_Label==1),2);
```

```
            Out_data{row, 5} = size(reference_Label,2);
            Out_data{row, 6} = unik_cutoff(cutoff);
            Out_data{row, 7} = size(find(reference_Label(testset)==0),2);
            Out_data{row, 8} = size(find(reference_Label(testset)==1),2);
            Out_data{row, 9} = size(reference_Label(testset),2);
            Out_data{row,10} =
size(find( (pred(testset)~=reference_Label(testset)') &
(reference_Label(testset)==0)' ),1);
            Out_data{row,11} =
size(find( (pred(testset)~=reference_Label(testset)') &
(reference_Label(testset)==1)' ),1);
            Out_data{row,12} =
size(find(pred(testset)~=reference_Label(testset)'),1);
            Out_data{row,13} = Out_data{row,10} / Out_data{row,7};
            Out_data{row,14} = Out_data{row,11} / Out_data{row,8};
            Out_data{row,15} = Out_data{row,12} / Out_data{row,9};
        end
    end
end

All_Out_data = [];
Out_data_mat = cell2mat(Out_data(2:end,:));
unik_cutoff  = unique(Out_data_mat(:,6));
for cutoff = 1 : size(unik_cutoff,1)
    Out_data_cutoff =
Out_data_mat(find(Out_data_mat(:,6)==unik_cutoff(cutoff)),:);
    [u v]           = sort(Out_data_cutoff(:,end));
    All_Out_data    = [All_Out_data;
Out_data_cutoff(v(find(u==u(1))),:)];
end

SVM2_Linear = [Out_data(1,:); num2cell(All_Out_data)];
save SVM2_RBF.mat;

% Discr2_Linear
%---------------
clear all
clc
load CD_from_PCDDB.mat;
load CD_from_Papers.mat;
load CD_from_Own_measurements.mat;
alldata_WL = [175:250]';
alldata_CD = [CD_PCDDB CD_Papers_ CD_Own];
alldata_Label = [Label_PCDDB Label_Papers_ Label_Own];
alldata_serial = [1:size(alldata_Label,2)];

alldata_minWL=zeros(1,size(alldata_CD,2));
for i=1:size(alldata_CD,2)
    alldata_minWL(1,i) = min(find(~isnan(alldata_CD(:,i))))+174;
end

Out_data = cell(1,15);
Out_data = {'WL1','WL2',...
            'Ordered reference',...
            'Unordered reference',...
            'Total reference',...
            'Cutoff',...
            'Ordered test',...
```

```
            'Unordered test',...
            'Total test',...
            'Ordered ERROR',...
            'Unordere ERROR',...
            'Total ERROR',...
            'Ordered ERROR Ratio',...
            'Unordered ERROR Ratio',...
            'Total ERROR Ratio'};
row = 1;
for wl1 = 175 : 250
    for wl2 = wl1+3 : 250
        tmp = find( ~isnan(alldata_CD(wl1-174,:)) &
~isnan(alldata_CD(wl2-174,:)) );
        reference_WL      = alldata_WL;
        reference_CD      = alldata_CD(:,tmp);
        reference_Label   = alldata_Label(:,tmp);
        reference_sorszam = alldata_sorszam(:,tmp);
        reference_minWL   = alldata_minWL(1,tmp);

        reference_preform = [reference_CD(wl1-174,:)' reference_CD(wl2-
174,:)' reference_Label'];

        pred=[];
        for CV = 1 : size(reference_CD,2)
            reference       = reference_preform;
            reference(CV,:) = [];
            Model           = fitcdiscr(reference(:,1:end-
1),reference(:,end),'DiscrimType','linear');
            pred(CV,1)      = Model.predict(reference_preform(CV,1:end-
1));
        end

        unik_cutoff = unique(reference_minWL)';
        for cutoff = 1 : size(unik_cutoff,1)
            testset = find(reference_minWL <= unik_cutoff(cutoff));
            row = row+1;
            Out_data{row, 1} = wl1;
            Out_data{row, 2} = wl2;
            Out_data{row, 3} = size(find(reference_Label==0),2);
            Out_data{row, 4} = size(find(reference_Label==1),2);
            Out_data{row, 5} = size(reference_Label,2);
            Out_data{row, 6} = unik_cutoff(cutoff);
            Out_data{row, 7} = size(find(reference_Label(testset)==0),2);
            Out_data{row, 8} = size(find(reference_Label(testset)==1),2);
            Out_data{row, 9} = size(reference_Label(testset),2);
            Out_data{row,10} =
size(find( (pred(testset)~=reference_Label(testset)') &
(reference_Label(testset)==0)' ),1);
            Out_data{row,11} =
size(find( (pred(testset)~=reference_Label(testset)') &
(reference_Label(testset)==1)' ),1);
            Out_data{row,12} =
size(find(pred(testset)~=reference_Label(testset)'),1);
            Out_data{row,13} = Out_data{row,10} / Out_data{row,7};
            Out_data{row,14} = Out_data{row,11} / Out_data{row,8};
            Out_data{row,15} = Out_data{row,12} / Out_data{row,9};
        end
    end
```

```
end

All_Out_data = [];
Out_data_mat = cell2mat(Out_data(2:end,:));
unik_cutoff  = unique(Out_data_mat(:,6));
for cutoff = 1 : size(unik_cutoff,1)
    Out_data_cutoff =
Out_data_mat(find(Out_data_mat(:,6)==unik_cutoff(cutoff)),:);
    [u v]           = sort(Out_data_cutoff(:,end));
    All_Out_data    = [All_Out_data;
Out_data_cutoff(v(find(u==u(1))),:)];
end

Discr2_Linear = [Out_data(1,:); num2cell(All_Out_data)];
save Discr2_Linear.mat;

% Discr2_Diaglinear
%-------------------
clear all
clc
load CD_from_PCDDB.mat;
load CD_from_Papers.mat;
load CD_from_Own_measurements.mat;
alldata_WL = [175:250]';
alldata_CD = [CD_PCDDB CD_Papers_ CD_Own];
alldata_Label = [Label_PCDDB Label_Papers_ Label_Own];
alldata_serial = [1:size(alldata_Label,2)];

alldata_minWL=zeros(1,size(alldata_CD,2));
for i=1:size(alldata_CD,2)
    alldata_minWL(1,i) = min(find(~isnan(alldata_CD(:,i))))+174;
end

Out_data = cell(1,15);
Out_data = {'WL1','WL2',...
            'Ordered reference',...
            'Unordered reference',...
            'Total reference',...
            'Cutoff',...
            'Ordered test',...
            'Unordered test',...
            'Total test',...
            'Ordered ERROR',...
            'Unordere ERROR',...
            'Total ERROR',...
            'Ordered ERROR Ratio',...
            'Unordered ERROR Ratio',...
            'Total ERROR Ratio'};
row = 1;
for wl1 = 175 : 250
    for wl2 = wl1+3 : 250
        tmp = find( ~isnan(alldata_CD(wl1-174,:)) &
~isnan(alldata_CD(wl2-174,:)) );
        reference_WL      = alldata_WL;
        reference_CD      = alldata_CD(:,tmp);
        reference_Label   = alldata_Label(:,tmp);
        reference_sorszam = alldata_sorszam(:,tmp);
        reference_minWL   = alldata_minWL(1,tmp);
```

```
        reference_preform = [reference_CD(wl1-174,:)' reference_CD(wl2-
174,:)' reference_Label'];

        pred=[];
        for CV = 1 : size(reference_CD,2)
            reference       = reference_preform;
            reference(CV,:) = [];
            Model           = fitcdiscr(reference(:,1:end-
1),reference(:,end),'DiscrimType','diaglinear');
            pred(CV,1)      = Model.predict(reference_preform(CV,1:end-
1));
        end

        unik_cutoff = unique(reference_minWL)';
        for cutoff = 1 : size(unik_cutoff,1)
            testset = find(reference_minWL <= unik_cutoff(cutoff));
            row = row+1;
            Out_data{row, 1} = wl1;
            Out_data{row, 2} = wl2;
            Out_data{row, 3} = size(find(reference_Label==0),2);
            Out_data{row, 4} = size(find(reference_Label==1),2);
            Out_data{row, 5} = size(reference_Label,2);
            Out_data{row, 6} = unik_cutoff(cutoff);
            Out_data{row, 7} = size(find(reference_Label(testset)==0),2);
            Out_data{row, 8} = size(find(reference_Label(testset)==1),2);
            Out_data{row, 9} = size(reference_Label(testset),2);
            Out_data{row,10} =
size(find( (pred(testset)~=reference_Label(testset)') &
(reference_Label(testset)==0)' ),1);
            Out_data{row,11} =
size(find( (pred(testset)~=reference_Label(testset)') &
(reference_Label(testset)==1)' ),1);
            Out_data{row,12} =
size(find(pred(testset)~=reference_Label(testset)'),1);
            Out_data{row,13} = Out_data{row,10} / Out_data{row,7};
            Out_data{row,14} = Out_data{row,11} / Out_data{row,8};
            Out_data{row,15} = Out_data{row,12} / Out_data{row,9};
        end
    end
end

All_Out_data = [];
Out_data_mat = cell2mat(Out_data(2:end,:));
unik_cutoff  = unique(Out_data_mat(:,6));
for cutoff = 1 : size(unik_cutoff,1)
    Out_data_cutoff =
Out_data_mat(find(Out_data_mat(:,6)==unik_cutoff(cutoff)),:);
    [u v]           = sort(Out_data_cutoff(:,end));
    All_Out_data    = [All_Out_data;
Out_data_cutoff(v(find(u==u(1))),:)];
end

Discr2_Diaglinear = [Out_data(1,:); num2cell(All_Out_data)];
save Discr2_Diaglinear.mat;

% Discr2_Quadratic
%------------------
```

```
clear all
clc
load CD_from_PCDDB.mat;
load CD_from_Papers.mat;
load CD_from_Own_measurements.mat;
alldata_WL = [175:250]';
alldata_CD = [CD_PCDDB CD_Papers_ CD_Own];
alldata_Label = [Label_PCDDB Label_Papers_ Label_Own];
alldata_serial = [1:size(alldata_Label,2)];

alldata_minWL=zeros(1,size(alldata_CD,2));
for i=1:size(alldata_CD,2)
    alldata_minWL(1,i) = min(find(~isnan(alldata_CD(:,i))))+174;
end

Out_data = cell(1,15);
Out_data = {'WL1','WL2',...
            'Ordered reference',...
            'Unordered reference',...
            'Total reference',...
            'Cutoff',...
            'Ordered test',...
            'Unordered test',...
            'Total test',...
            'Ordered ERROR',...
            'Unordere ERROR',...
            'Total ERROR',...
            'Ordered ERROR Ratio',...
            'Unordered ERROR Ratio',...
            'Total ERROR Ratio'};
row = 1;
for wl1 = 175 : 250
    for wl2 = wl1+3 : 250
        tmp = find( ~isnan(alldata_CD(wl1-174,:)) &
~isnan(alldata_CD(wl2-174,:)) );
        reference_WL      = alldata_WL;
        reference_CD      = alldata_CD(:,tmp);
        reference_Label   = alldata_Label(:,tmp);
        reference_sorszam = alldata_sorszam(:,tmp);
        reference_minWL   = alldata_minWL(1,tmp);

        reference_preform = [reference_CD(wl1-174,:)' reference_CD(wl2-
174,:)' reference_Label'];

        pred=[];
        for CV = 1 : size(reference_CD,2)
            reference       = reference_preform;
            reference(CV,:) = [];
            Model           = fitcdiscr(reference(:,1:end-
1),reference(:,end),'DiscrimType','quadratic');
            pred(CV,1)      = Model.predict(reference_preform(CV,1:end-
1));
        end

        unik_cutoff = unique(reference_minWL)';
        for cutoff = 1 : size(unik_cutoff,1)
            testset = find(reference_minWL <= unik_cutoff(cutoff));
            row = row+1;
```

```
            Out_data{row, 1} = wl1;
            Out_data{row, 2} = wl2;
            Out_data{row, 3} = size(find(reference_Label==0),2);
            Out_data{row, 4} = size(find(reference_Label==1),2);
            Out_data{row, 5} = size(reference_Label,2);
            Out_data{row, 6} = unik_cutoff(cutoff);
            Out_data{row, 7} = size(find(reference_Label(testset)==0),2);
            Out_data{row, 8} = size(find(reference_Label(testset)==1),2);
            Out_data{row, 9} = size(reference_Label(testset),2);
            Out_data{row,10} =
size(find( (pred(testset)~=reference_Label(testset)') &
(reference_Label(testset)==0)' ),1);
            Out_data{row,11} =
size(find( (pred(testset)~=reference_Label(testset)') &
(reference_Label(testset)==1)' ),1);
            Out_data{row,12} =
size(find(pred(testset)~=reference_Label(testset)'),1);
            Out_data{row,13} = Out_data{row,10} / Out_data{row,7};
            Out_data{row,14} = Out_data{row,11} / Out_data{row,8};
            Out_data{row,15} = Out_data{row,12} / Out_data{row,9};
        end
    end
end

All_Out_data = [];
Out_data_mat = cell2mat(Out_data(2:end,:));
unik_cutoff  = unique(Out_data_mat(:,6));
for cutoff = 1 : size(unik_cutoff,1)
    Out_data_cutoff =
Out_data_mat(find(Out_data_mat(:,6)==unik_cutoff(cutoff)),:);
    [u v]           = sort(Out_data_cutoff(:,end));
    All_Out_data    = [All_Out_data;
Out_data_cutoff(v(find(u==u(1))),:)];
end

Discr2_Quadratic = [Out_data(1,:); num2cell(All_Out_data)];
save Discr2_Quadratic.mat;

% Discr2_Diagquadratic
%----------------------
clear all
clc
load CD_from_PCDDB.mat;
load CD_from_Papers.mat;
load CD_from_Own_measurements.mat;
alldata_WL = [175:250]';
alldata_CD = [CD_PCDDB CD_Papers_ CD_Own];
alldata_Label = [Label_PCDDB Label_Papers_ Label_Own];
alldata_serial = [1:size(alldata_Label,2)];

alldata_minWL=zeros(1,size(alldata_CD,2));
for i=1:size(alldata_CD,2)
    alldata_minWL(1,i) = min(find(~isnan(alldata_CD(:,i))))+174;
end

Out_data = cell(1,15);
Out_data = {'WL1','WL2',...
            'Ordered reference',...
```

```
            'Unordered reference',...
            'Total reference',...
            'Cutoff',...
            'Ordered test',...
            'Unordered test',...
            'Total test',...
            'Ordered ERROR',...
            'Unordere ERROR',...
            'Total ERROR',...
            'Ordered ERROR Ratio',...
            'Unordered ERROR Ratio',...
            'Total ERROR Ratio'};
row = 1;
for wl1 = 175 : 250
    for wl2 = wl1+3 : 250
        tmp = find( ~isnan(alldata_CD(wl1-174,:)) &
~isnan(alldata_CD(wl2-174,:)) );
        reference_WL      = alldata_WL;
        reference_CD      = alldata_CD(:,tmp);
        reference_Label   = alldata_Label(:,tmp);
        reference_sorszam = alldata_sorszam(:,tmp);
        reference_minWL   = alldata_minWL(1,tmp);

        reference_preform = [reference_CD(wl1-174,:)' reference_CD(wl2-
174,:)' reference_Label'];

        pred=[];
        for CV = 1 : size(reference_CD,2)
            reference       = reference_preform;
            reference(CV,:) = [];
            Model           = fitcdiscr(reference(:,1:end-
1),reference(:,end),'DiscrimType','diagquadratic');
            pred(CV,1)      = Model.predict(reference_preform(CV,1:end-
1));
        end

        unik_cutoff = unique(reference_minWL)';
        for cutoff = 1 : size(unik_cutoff,1)
            testset = find(reference_minWL <= unik_cutoff(cutoff));
            row = row+1;
            Out_data{row, 1} = wl1;
            Out_data{row, 2} = wl2;
            Out_data{row, 3} = size(find(reference_Label==0),2);
            Out_data{row, 4} = size(find(reference_Label==1),2);
            Out_data{row, 5} = size(reference_Label,2);
            Out_data{row, 6} = unik_cutoff(cutoff);
            Out_data{row, 7} = size(find(reference_Label(testset)==0),2);
            Out_data{row, 8} = size(find(reference_Label(testset)==1),2);
            Out_data{row, 9} = size(reference_Label(testset),2);
            Out_data{row,10} =
size(find( (pred(testset)~=reference_Label(testset)') &
(reference_Label(testset)==0)' ),1);
            Out_data{row,11} =
size(find( (pred(testset)~=reference_Label(testset)') &
(reference_Label(testset)==1)' ),1);
            Out_data{row,12} =
size(find(pred(testset)~=reference_Label(testset)'),1);
            Out_data{row,13} = Out_data{row,10} / Out_data{row,7};
```

```
            Out_data{row,14} = Out_data{row,11} / Out_data{row,8};
            Out_data{row,15} = Out_data{row,12} / Out_data{row,9};
        end
    end
end

All_Out_data = [];
Out_data_mat = cell2mat(Out_data(2:end,:));
unik_cutoff  = unique(Out_data_mat(:,6));
for cutoff = 1 : size(unik_cutoff,1)
    Out_data_cutoff =
Out_data_mat(find(Out_data_mat(:,6)==unik_cutoff(cutoff)),:);
    [u v]           = sort(Out_data_cutoff(:,end));
    All_Out_data    = [All_Out_data;
Out_data_cutoff(v(find(u==u(1))),:)];
end

Discr2_Diagquadratic = [Out_data(1,:); num2cell(All_Out_data)];
save Discr2_Diagquadratic.mat;

% Tree2_Simple
%--------------
clear all
clc
load CD_from_PCDDB.mat;
load CD_from_Papers.mat;
load CD_from_Own_measurements.mat;
alldata_WL = [175:250]';
alldata_CD = [CD_PCDDB CD_Papers_ CD_Own];
alldata_Label = [Label_PCDDB Label_Papers_ Label_Own];
alldata_serial = [1:size(alldata_Label,2)];

alldata_minWL=zeros(1,size(alldata_CD,2));
for i=1:size(alldata_CD,2)
    alldata_minWL(1,i) = min(find(~isnan(alldata_CD(:,i))))+174;
end

Out_data = cell(1,15);
Out_data = {'WL1','WL2',...
            'Ordered reference',...
            'Unordered reference',...
            'Total reference',...
            'Cutoff',...
            'Ordered test',...
            'Unordered test',...
            'Total test',...
            'Ordered ERROR',...
            'Unordere ERROR',...
            'Total ERROR',...
            'Ordered ERROR Ratio',...
            'Unordered ERROR Ratio',...
            'Total ERROR Ratio'};
row = 1;
for wl1 = 175 : 250
    for wl2 = wl1+3 : 250
        tmp = find( ~isnan(alldata_CD(wl1-174,:)) &
~isnan(alldata_CD(wl2-174,:)) );
        reference_WL      = alldata_WL;
```

```
        reference_CD      = alldata_CD(:,tmp);
        reference_Label   = alldata_Label(:,tmp);
        reference_sorszam = alldata_sorszam(:,tmp);
        reference_minWL   = alldata_minWL(1,tmp);

        reference_preform = [reference_CD(wl1-174,:)' reference_CD(wl2-
174,:)' reference_Label'];

        pred=[];
        for CV = 1 : size(reference_CD,2)
            reference       = reference_preform;
            reference(CV,:) = [];
            Model           = fitctree(reference(:,1:end-
1),reference(:,end),'MaxNumSplits', 4);
            pred(CV,1)      = Model.predict(reference_preform(CV,1:end-
1));
        end

        unik_cutoff = unique(reference_minWL)';
        for cutoff = 1 : size(unik_cutoff,1)
            testset = find(reference_minWL <= unik_cutoff(cutoff));
            row = row+1;
            Out_data{row, 1} = wl1;
            Out_data{row, 2} = wl2;
            Out_data{row, 3} = size(find(reference_Label==0),2);
            Out_data{row, 4} = size(find(reference_Label==1),2);
            Out_data{row, 5} = size(reference_Label,2);
            Out_data{row, 6} = unik_cutoff(cutoff);
            Out_data{row, 7} = size(find(reference_Label(testset)==0),2);
            Out_data{row, 8} = size(find(reference_Label(testset)==1),2);
            Out_data{row, 9} = size(reference_Label(testset),2);
            Out_data{row,10} =
size(find( (pred(testset)~=reference_Label(testset)') &
(reference_Label(testset)==0)' ),1);
            Out_data{row,11} =
size(find( (pred(testset)~=reference_Label(testset)') &
(reference_Label(testset)==1)' ),1);
            Out_data{row,12} =
size(find(pred(testset)~=reference_Label(testset)'),1);
            Out_data{row,13} = Out_data{row,10} / Out_data{row,7};
            Out_data{row,14} = Out_data{row,11} / Out_data{row,8};
            Out_data{row,15} = Out_data{row,12} / Out_data{row,9};
        end
    end
end

All_Out_data = [];
Out_data_mat = cell2mat(Out_data(2:end,:));
unik_cutoff  = unique(Out_data_mat(:,6));
for cutoff = 1 : size(unik_cutoff,1)
    Out_data_cutoff =
Out_data_mat(find(Out_data_mat(:,6)==unik_cutoff(cutoff)),:);
    [u v]           = sort(Out_data_cutoff(:,end));
    All_Out_data    = [All_Out_data;
Out_data_cutoff(v(find(u==u(1))),:)];
end

Tree2_Simple = [Out_data(1,:); num2cell(All_Out_data)];
```

```
save Tree2_Simple.mat;

% Tree2_Medium
%--------------
clear all
clc
load CD_from_PCDDB.mat;
load CD_from_Papers.mat;
load CD_from_Own_measurements.mat;
alldata_WL = [175:250]';
alldata_CD = [CD_PCDDB CD_Papers_ CD_Own];
alldata_Label = [Label_PCDDB Label_Papers_ Label_Own];
alldata_serial = [1:size(alldata_Label,2)];

alldata_minWL=zeros(1,size(alldata_CD,2));
for i=1:size(alldata_CD,2)
    alldata_minWL(1,i) = min(find(~isnan(alldata_CD(:,i))))+174;
end

Out_data = cell(1,15);
Out_data = {'WL1','WL2',...
            'Ordered reference',...
            'Unordered reference',...
            'Total reference',...
            'Cutoff',...
            'Ordered test',...
            'Unordered test',...
            'Total test',...
            'Ordered ERROR',...
            'Unordere ERROR',...
            'Total ERROR',...
            'Ordered ERROR Ratio',...
            'Unordered ERROR Ratio',...
            'Total ERROR Ratio'};
row = 1;
for wl1 = 175 : 250
    for wl2 = wl1+3 : 250
        tmp = find( ~isnan(alldata_CD(wl1-174,:)) &
~isnan(alldata_CD(wl2-174,:)) );
        reference_WL      = alldata_WL;
        reference_CD      = alldata_CD(:,tmp);
        reference_Label   = alldata_Label(:,tmp);
        reference_sorszam = alldata_sorszam(:,tmp);
        reference_minWL   = alldata_minWL(1,tmp);

        reference_preform = [reference_CD(wl1-174,:)' reference_CD(wl2-
174,:)' reference_Label'];

        pred=[];
        for CV = 1 : size(reference_CD,2)
            reference       = reference_preform;
            reference(CV,:) = [];
            Model           = fitctree(reference(:,1:end-
1),reference(:,end),'MaxNumSplits', 20);
            pred(CV,1)      = Model.predict(reference_preform(CV,1:end-
1));
        end
```

```
        unik_cutoff = unique(reference_minWL)';
        for cutoff = 1 : size(unik_cutoff,1)
            testset = find(reference_minWL <= unik_cutoff(cutoff));
            row = row+1;
            Out_data{row, 1} = wl1;
            Out_data{row, 2} = wl2;
            Out_data{row, 3} = size(find(reference_Label==0),2);
            Out_data{row, 4} = size(find(reference_Label==1),2);
            Out_data{row, 5} = size(reference_Label,2);
            Out_data{row, 6} = unik_cutoff(cutoff);
            Out_data{row, 7} = size(find(reference_Label(testset)==0),2);
            Out_data{row, 8} = size(find(reference_Label(testset)==1),2);
            Out_data{row, 9} = size(reference_Label(testset),2);
            Out_data{row,10} =
size(find( (pred(testset)~=reference_Label(testset)') &
(reference_Label(testset)==0)' ),1);
            Out_data{row,11} =
size(find( (pred(testset)~=reference_Label(testset)') &
(reference_Label(testset)==1)' ),1);
            Out_data{row,12} =
size(find(pred(testset)~=reference_Label(testset)'),1);
            Out_data{row,13} = Out_data{row,10} / Out_data{row,7};
            Out_data{row,14} = Out_data{row,11} / Out_data{row,8};
            Out_data{row,15} = Out_data{row,12} / Out_data{row,9};
        end
    end
end

All_Out_data = [];
Out_data_mat = cell2mat(Out_data(2:end,:));
unik_cutoff  = unique(Out_data_mat(:,6));
for cutoff = 1 : size(unik_cutoff,1)
    Out_data_cutoff =
Out_data_mat(find(Out_data_mat(:,6)==unik_cutoff(cutoff)),:);
    [u v]           = sort(Out_data_cutoff(:,end));
    All_Out_data    = [All_Out_data;
Out_data_cutoff(v(find(u==u(1))),:)];
end

Tree2_Medium = [Out_data(1,:); num2cell(All_Out_data)];
save Tree2_Medium.mat;

% Tree2_Complex
%---------------
clear all
clc
load CD_from_PCDDB.mat;
load CD_from_Papers.mat;
load CD_from_Own_measurements.mat;
alldata_WL = [175:250]';
alldata_CD = [CD_PCDDB CD_Papers_ CD_Own];
alldata_Label = [Label_PCDDB Label_Papers_ Label_Own];
alldata_serial = [1:size(alldata_Label,2)];

alldata_minWL=zeros(1,size(alldata_CD,2));
for i=1:size(alldata_CD,2)
    alldata_minWL(1,i) = min(find(~isnan(alldata_CD(:,i))))+174;
end
```

```
Out_data = cell(1,15);
Out_data = {'WL1','WL2',...
            'Ordered reference',...
            'Unordered reference',...
            'Total reference',...
            'Cutoff',...
            'Ordered test',...
            'Unordered test',...
            'Total test',...
            'Ordered ERROR',...
            'Unordere ERROR',...
            'Total ERROR',...
            'Ordered ERROR Ratio',...
            'Unordered ERROR Ratio',...
            'Total ERROR Ratio'};
row = 1;
for wl1 = 175 : 250
    for wl2 = wl1+3 : 250
        tmp = find( ~isnan(alldata_CD(wl1-174,:)) &
~isnan(alldata_CD(wl2-174,:)) );
        reference_WL      = alldata_WL;
        reference_CD      = alldata_CD(:,tmp);
        reference_Label   = alldata_Label(:,tmp);
        reference_sorszam = alldata_sorszam(:,tmp);
        reference_minWL   = alldata_minWL(1,tmp);

        reference_preform = [reference_CD(wl1-174,:)' reference_CD(wl2-
174,:)' reference_Label'];

        pred=[];
        for CV = 1 : size(reference_CD,2)
            reference       = reference_preform;
            reference(CV,:) = [];
            Model           = fitctree(reference(:,1:end-
1),reference(:,end),'MaxNumSplits', 100);
            pred(CV,1)      = Model.predict(reference_preform(CV,1:end-
1));
        end

        unik_cutoff = unique(reference_minWL)';
        for cutoff = 1 : size(unik_cutoff,1)
            testset = find(reference_minWL <= unik_cutoff(cutoff));
            row = row+1;
            Out_data{row, 1} = wl1;
            Out_data{row, 2} = wl2;
            Out_data{row, 3} = size(find(reference_Label==0),2);
            Out_data{row, 4} = size(find(reference_Label==1),2);
            Out_data{row, 5} = size(reference_Label,2);
            Out_data{row, 6} = unik_cutoff(cutoff);
            Out_data{row, 7} = size(find(reference_Label(testset)==0),2);
            Out_data{row, 8} = size(find(reference_Label(testset)==1),2);
            Out_data{row, 9} = size(reference_Label(testset),2);
            Out_data{row,10} =
size(find( (pred(testset)~=reference_Label(testset)') &
(reference_Label(testset)==0)' ),1);
```

```
            Out_data{row,11} =
size(find( (pred(testset)~=reference_Label(testset)') &
(reference_Label(testset)==1)' ),1);
            Out_data{row,12} =
size(find(pred(testset)~=reference_Label(testset)'),1);
            Out_data{row,13} = Out_data{row,10} / Out_data{row,7};
            Out_data{row,14} = Out_data{row,11} / Out_data{row,8};
            Out_data{row,15} = Out_data{row,12} / Out_data{row,9};
        end
    end
end

All_Out_data = [];
Out_data_mat = cell2mat(Out_data(2:end,:));
unik_cutoff  = unique(Out_data_mat(:,6));
for cutoff = 1 : size(unik_cutoff,1)
    Out_data_cutoff =
Out_data_mat(find(Out_data_mat(:,6)==unik_cutoff(cutoff)),:);
    [u v]           = sort(Out_data_cutoff(:,end));
    All_Out_data    = [All_Out_data;
Out_data_cutoff(v(find(u==u(1))),:)];
end

Tree2_Complex = [Out_data(1,:); num2cell(All_Out_data)];
save Tree2_Complex.mat;


% KNN2_Fine
%-----------
clear all
clc
load CD_from_PCDDB.mat;
load CD_from_Papers.mat;
load CD_from_Own_measurements.mat;
alldata_WL = [175:250]';
alldata_CD = [CD_PCDDB CD_Papers_ CD_Own];
alldata_Label = [Label_PCDDB Label_Papers_ Label_Own];
alldata_serial = [1:size(alldata_Label,2)];

alldata_minWL=zeros(1,size(alldata_CD,2));
for i=1:size(alldata_CD,2)
    alldata_minWL(1,i) = min(find(~isnan(alldata_CD(:,i))))+174;
end

Out_data = cell(1,15);
Out_data = {'WL1','WL2',...
            'Ordered reference',...
            'Unordered reference',...
            'Total reference',...
            'Cutoff',...
            'Ordered test',...
            'Unordered test',...
            'Total test',...
            'Ordered ERROR',...
            'Unordere ERROR',...
            'Total ERROR',...
            'Ordered ERROR Ratio',...
            'Unordered ERROR Ratio',...
```

```
            'Total ERROR Ratio'};
row = 1;
for wl1 = 175 : 250
    for wl2 = wl1+3 : 250
        tmp = find( ~isnan(alldata_CD(wl1-174,:)) &
~isnan(alldata_CD(wl2-174,:)) );
        reference_WL      = alldata_WL;
        reference_CD      = alldata_CD(:,tmp);
        reference_Label   = alldata_Label(:,tmp);
        reference_sorszam = alldata_sorszam(:,tmp);
        reference_minWL   = alldata_minWL(1,tmp);

        reference_preform = [reference_CD(wl1-174,:)' reference_CD(wl2-
174,:)' reference_Label'];

        pred=[];
        for CV = 1 : size(reference_CD,2)
            reference       = reference_preform;
            reference(CV,:) = [];
            Model           = fitcknn(reference(:,1:end-
1),reference(:,end),'Distance', 'Euclidean','Exponent',
[],'NumNeighbors', 1,'DistanceWeight', 'Equal','Standardize', false);
            pred(CV,1)      = Model.predict(reference_preform(CV,1:end-
1));
        end

        unik_cutoff = unique(reference_minWL)';
        for cutoff = 1 : size(unik_cutoff,1)
            testset = find(reference_minWL <= unik_cutoff(cutoff));
            row = row+1;
            Out_data{row, 1} = wl1;
            Out_data{row, 2} = wl2;
            Out_data{row, 3} = size(find(reference_Label==0),2);
            Out_data{row, 4} = size(find(reference_Label==1),2);
            Out_data{row, 5} = size(reference_Label,2);
            Out_data{row, 6} = unik_cutoff(cutoff);
            Out_data{row, 7} = size(find(reference_Label(testset)==0),2);
            Out_data{row, 8} = size(find(reference_Label(testset)==1),2);
            Out_data{row, 9} = size(reference_Label(testset),2);
            Out_data{row,10} =
size(find( (pred(testset)~=reference_Label(testset)') &
(reference_Label(testset)==0)' ),1);
            Out_data{row,11} =
size(find( (pred(testset)~=reference_Label(testset)') &
(reference_Label(testset)==1)' ),1);
            Out_data{row,12} =
size(find(pred(testset)~=reference_Label(testset)'),1);
            Out_data{row,13} = Out_data{row,10} / Out_data{row,7};
            Out_data{row,14} = Out_data{row,11} / Out_data{row,8};
            Out_data{row,15} = Out_data{row,12} / Out_data{row,9};
        end
    end
end

All_Out_data = [];
Out_data_mat = cell2mat(Out_data(2:end,:));
unik_cutoff  = unique(Out_data_mat(:,6));
for cutoff = 1 : size(unik_cutoff,1)
```

```
    Out_data_cutoff =
Out_data_mat(find(Out_data_mat(:,6)==unik_cutoff(cutoff)),:);
    [u v]           = sort(Out_data_cutoff(:,end));
    All_Out_data    = [All_Out_data;
Out_data_cutoff(v(find(u==u(1))),:)];
end

KNN2_Fine = [Out_data(1,:); num2cell(All_Out_data)];
save KNN2_Fine.mat;

% KNN2_Medium
%-------------
clear all
clc
load CD_from_PCDDB.mat;
load CD_from_Papers.mat;
load CD_from_Own_measurements.mat;
alldata_WL = [175:250]';
alldata_CD = [CD_PCDDB CD_Papers_ CD_Own];
alldata_Label = [Label_PCDDB Label_Papers_ Label_Own];
alldata_serial = [1:size(alldata_Label,2)];

alldata_minWL=zeros(1,size(alldata_CD,2));
for i=1:size(alldata_CD,2)
    alldata_minWL(1,i) = min(find(~isnan(alldata_CD(:,i))))+174;
end

Out_data = cell(1,15);
Out_data = {'WL1','WL2',...
            'Ordered reference',...
            'Unordered reference',...
            'Total reference',...
            'Cutoff',...
            'Ordered test',...
            'Unordered test',...
            'Total test',...
            'Ordered ERROR',...
            'Unordere ERROR',...
            'Total ERROR',...
            'Ordered ERROR Ratio',...
            'Unordered ERROR Ratio',...
            'Total ERROR Ratio'};
row = 1;
for wl1 = 175 : 250
    for wl2 = wl1+3 : 250
        tmp = find( ~isnan(alldata_CD(wl1-174,:)) &
~isnan(alldata_CD(wl2-174,:)) );
        reference_WL      = alldata_WL;
        reference_CD      = alldata_CD(:,tmp);
        reference_Label   = alldata_Label(:,tmp);
        reference_sorszam = alldata_sorszam(:,tmp);
        reference_minWL   = alldata_minWL(1,tmp);

        reference_preform = [reference_CD(wl1-174,:)' reference_CD(wl2-
174,:)' reference_Label'];

        pred=[];
        for CV = 1 : size(reference_CD,2)
```

```
            reference       = reference_preform;
            reference(CV,:) = [];
            Model           = fitcknn(reference(:,1:end-
1),reference(:,end),'Distance', 'Euclidean','Exponent',
[],'NumNeighbors', 10,'DistanceWeight', 'Equal','Standardize', false);
            pred(CV,1)      = Model.predict(reference_preform(CV,1:end-
1));
        end

        unik_cutoff = unique(reference_minWL)';
        for cutoff = 1 : size(unik_cutoff,1)
            testset = find(reference_minWL <= unik_cutoff(cutoff));
            row = row+1;
            Out_data{row, 1} = wl1;
            Out_data{row, 2} = wl2;
            Out_data{row, 3} = size(find(reference_Label==0),2);
            Out_data{row, 4} = size(find(reference_Label==1),2);
            Out_data{row, 5} = size(reference_Label,2);
            Out_data{row, 6} = unik_cutoff(cutoff);
            Out_data{row, 7} = size(find(reference_Label(testset)==0),2);
            Out_data{row, 8} = size(find(reference_Label(testset)==1),2);
            Out_data{row, 9} = size(reference_Label(testset),2);
            Out_data{row,10} =
size(find( (pred(testset)~=reference_Label(testset)') &
(reference_Label(testset)==0)' ),1);
            Out_data{row,11} =
size(find( (pred(testset)~=reference_Label(testset)') &
(reference_Label(testset)==1)' ),1);
            Out_data{row,12} =
size(find(pred(testset)~=reference_Label(testset)'),1);
            Out_data{row,13} = Out_data{row,10} / Out_data{row,7};
            Out_data{row,14} = Out_data{row,11} / Out_data{row,8};
            Out_data{row,15} = Out_data{row,12} / Out_data{row,9};
        end
    end
end

All_Out_data = [];
Out_data_mat = cell2mat(Out_data(2:end,:));
unik_cutoff  = unique(Out_data_mat(:,6));
for cutoff = 1 : size(unik_cutoff,1)
    Out_data_cutoff =
Out_data_mat(find(Out_data_mat(:,6)==unik_cutoff(cutoff)),:);
    [u v]           = sort(Out_data_cutoff(:,end));
    All_Out_data    = [All_Out_data;
Out_data_cutoff(v(find(u==u(1))),:)];
end

KNN2_Medium = [Out_data(1,:); num2cell(All_Out_data)];
save KNN2_Medium.mat;

% KNN2_Coarse
%-------------
clear all
clc
load CD_from_PCDDB.mat;
load CD_from_Papers.mat;
load CD_from_Own_measurements.mat;
```

```
alldata_WL = [175:250]';
alldata_CD = [CD_PCDDB CD_Papers_ CD_Own];
alldata_Label = [Label_PCDDB Label_Papers_ Label_Own];
alldata_serial = [1:size(alldata_Label,2)];

alldata_minWL=zeros(1,size(alldata_CD,2));
for i=1:size(alldata_CD,2)
    alldata_minWL(1,i) = min(find(~isnan(alldata_CD(:,i))))+174;
end

Out_data = cell(1,15);
Out_data = {'WL1','WL2',...
            'Ordered reference',...
            'Unordered reference',...
            'Total reference',...
            'Cutoff',...
            'Ordered test',...
            'Unordered test',...
            'Total test',...
            'Ordered ERROR',...
            'Unordere ERROR',...
            'Total ERROR',...
            'Ordered ERROR Ratio',...
            'Unordered ERROR Ratio',...
            'Total ERROR Ratio'};
row = 1;
for wl1 = 175 : 250
    for wl2 = wl1+3 : 250
        tmp = find( ~isnan(alldata_CD(wl1-174,:)) &
~isnan(alldata_CD(wl2-174,:)) );
        reference_WL      = alldata_WL;
        reference_CD      = alldata_CD(:,tmp);
        reference_Label   = alldata_Label(:,tmp);
        reference_sorszam = alldata_sorszam(:,tmp);
        reference_minWL   = alldata_minWL(1,tmp);

        reference_preform = [reference_CD(wl1-174,:)' reference_CD(wl2-
174,:)' reference_Label'];

        pred=[];
        for CV = 1 : size(reference_CD,2)
            reference       = reference_preform;
            reference(CV,:) = [];
            Model           = fitcknn(reference(:,1:end-
1),reference(:,end),'Distance', 'Euclidean','Exponent',
[],'NumNeighbors', 100,'DistanceWeight', 'Equal','Standardize', false);
            pred(CV,1)      = Model.predict(reference_preform(CV,1:end-
1));
        end

        unik_cutoff = unique(reference_minWL)';
        for cutoff = 1 : size(unik_cutoff,1)
            testset = find(reference_minWL <= unik_cutoff(cutoff));
            row = row+1;
            Out_data{row, 1} = wl1;
            Out_data{row, 2} = wl2;
            Out_data{row, 3} = size(find(reference_Label==0),2);
            Out_data{row, 4} = size(find(reference_Label==1),2);
```

```
            Out_data{row, 5} = size(reference_Label,2);
            Out_data{row, 6} = unik_cutoff(cutoff);
            Out_data{row, 7} = size(find(reference_Label(testset)==0),2);
            Out_data{row, 8} = size(find(reference_Label(testset)==1),2);
            Out_data{row, 9} = size(reference_Label(testset),2);
            Out_data{row,10} =
size(find( (pred(testset)~=reference_Label(testset)') &
(reference_Label(testset)==0)' ),1);
            Out_data{row,11} =
size(find( (pred(testset)~=reference_Label(testset)') &
(reference_Label(testset)==1)' ),1);
            Out_data{row,12} =
size(find(pred(testset)~=reference_Label(testset)'),1);
            Out_data{row,13} = Out_data{row,10} / Out_data{row,7};
            Out_data{row,14} = Out_data{row,11} / Out_data{row,8};
            Out_data{row,15} = Out_data{row,12} / Out_data{row,9};
        end
    end
end

All_Out_data = [];
Out_data_mat = cell2mat(Out_data(2:end,:));
unik_cutoff  = unique(Out_data_mat(:,6));
for cutoff = 1 : size(unik_cutoff,1)
    Out_data_cutoff =
Out_data_mat(find(Out_data_mat(:,6)==unik_cutoff(cutoff)),:);
    [u v]           = sort(Out_data_cutoff(:,end));
    All_Out_data    = [All_Out_data;
Out_data_cutoff(v(find(u==u(1))),:)];
end

KNN2_Coarse = [Out_data(1,:); num2cell(All_Out_data)];
save KNN2_Coarse.mat;

% KNN2_Cosine
%-------------
clear all
clc
load CD_from_PCDDB.mat;
load CD_from_Papers.mat;
load CD_from_Own_measurements.mat;
alldata_WL = [175:250]';
alldata_CD = [CD_PCDDB CD_Papers_ CD_Own];
alldata_Label = [Label_PCDDB Label_Papers_ Label_Own];
alldata_serial = [1:size(alldata_Label,2)];

alldata_minWL=zeros(1,size(alldata_CD,2));
for i=1:size(alldata_CD,2)
    alldata_minWL(1,i) = min(find(~isnan(alldata_CD(:,i))))+174;
end

Out_data = cell(1,15);
Out_data = {'WL1','WL2',...
            'Ordered reference',...
            'Unordered reference',...
            'Total reference',...
            'Cutoff',...
            'Ordered test',...
```

```
            'Unordered test',...
            'Total test',...
            'Ordered ERROR',...
            'Unordere ERROR',...
            'Total ERROR',...
            'Ordered ERROR Ratio',...
            'Unordered ERROR Ratio',...
            'Total ERROR Ratio'};
row = 1;
for wl1 = 175 : 250
    for wl2 = wl1+3 : 250
        tmp = find( ~isnan(alldata_CD(wl1-174,:)) &
~isnan(alldata_CD(wl2-174,:)) );
        reference_WL      = alldata_WL;
        reference_CD      = alldata_CD(:,tmp);
        reference_Label   = alldata_Label(:,tmp);
        reference_sorszam = alldata_sorszam(:,tmp);
        reference_minWL   = alldata_minWL(1,tmp);

        reference_preform = [reference_CD(wl1-174,:)' reference_CD(wl2-
174,:)' reference_Label'];

        pred=[];
        for CV = 1 : size(reference_CD,2)
            reference       = reference_preform;
            reference(CV,:) = [];
            Model           = fitcknn(reference(:,1:end-
1),reference(:,end),'Distance', 'Cosine','Exponent', [],'NumNeighbors',
10,'DistanceWeight', 'Equal','Standardize', false);
            pred(CV,1)      = Model.predict(reference_preform(CV,1:end-
1));
        end

        unik_cutoff = unique(reference_minWL)';
        for cutoff = 1 : size(unik_cutoff,1)
            testset = find(reference_minWL <= unik_cutoff(cutoff));
            row = row+1;
            Out_data{row, 1} = wl1;
            Out_data{row, 2} = wl2;
            Out_data{row, 3} = size(find(reference_Label==0),2);
            Out_data{row, 4} = size(find(reference_Label==1),2);
            Out_data{row, 5} = size(reference_Label,2);
            Out_data{row, 6} = unik_cutoff(cutoff);
            Out_data{row, 7} = size(find(reference_Label(testset)==0),2);
            Out_data{row, 8} = size(find(reference_Label(testset)==1),2);
            Out_data{row, 9} = size(reference_Label(testset),2);
            Out_data{row,10} =
size(find( (pred(testset)~=reference_Label(testset)') &
(reference_Label(testset)==0)' ),1);
            Out_data{row,11} =
size(find( (pred(testset)~=reference_Label(testset)') &
(reference_Label(testset)==1)' ),1);
            Out_data{row,12} =
size(find(pred(testset)~=reference_Label(testset)'),1);
            Out_data{row,13} = Out_data{row,10} / Out_data{row,7};
            Out_data{row,14} = Out_data{row,11} / Out_data{row,8};
            Out_data{row,15} = Out_data{row,12} / Out_data{row,9};
        end
```

```
    end
end

All_Out_data = [];
Out_data_mat = cell2mat(Out_data(2:end,:));
unik_cutoff  = unique(Out_data_mat(:,6));
for cutoff = 1 : size(unik_cutoff,1)
    Out_data_cutoff =
Out_data_mat(find(Out_data_mat(:,6)==unik_cutoff(cutoff)),:);
    [u v]          = sort(Out_data_cutoff(:,end));
    All_Out_data    = [All_Out_data;
Out_data_cutoff(v(find(u==u(1))),:)];
end

KNN2_Cosine = [Out_data(1,:); num2cell(All_Out_data)];
save KNN2_Cosine.mat;

% KNN2_Wighted
%--------------
clear all
clc
load CD_from_PCDDB.mat;
load CD_from_Papers.mat;
load CD_from_Own_measurements.mat;
alldata_WL = [175:250]';
alldata_CD = [CD_PCDDB CD_Papers_ CD_Own];
alldata_Label = [Label_PCDDB Label_Papers_ Label_Own];
alldata_serial = [1:size(alldata_Label,2)];

alldata_minWL=zeros(1,size(alldata_CD,2));
for i=1:size(alldata_CD,2)
    alldata_minWL(1,i) = min(find(~isnan(alldata_CD(:,i))))+174;
end

Out_data = cell(1,15);
Out_data = {'WL1','WL2',...
            'Ordered reference',...
            'Unordered reference',...
            'Total reference',...
            'Cutoff',...
            'Ordered test',...
            'Unordered test',...
            'Total test',...
            'Ordered ERROR',...
            'Unordere ERROR',...
            'Total ERROR',...
            'Ordered ERROR Ratio',...
            'Unordered ERROR Ratio',...
            'Total ERROR Ratio'};
row = 1;
for wl1 = 175 : 250
    for wl2 = wl1+3 : 250
        tmp = find( ~isnan(alldata_CD(wl1-174,:)) &
~isnan(alldata_CD(wl2-174,:)) );
        reference_WL      = alldata_WL;
        reference_CD      = alldata_CD(:,tmp);
        reference_Label   = alldata_Label(:,tmp);
        reference_sorszam = alldata_sorszam(:,tmp);
```

```
        reference_minWL   = alldata_minWL(1,tmp);

        reference_preform = [reference_CD(wl1-174,:)' reference_CD(wl2-
174,:)' reference_Label'];

        pred=[];
        for CV = 1 : size(reference_CD,2)
            reference       = reference_preform;
            reference(CV,:) = [];
            Model           = fitcknn(reference(:,1:end-
1),reference(:,end),'Distance', 'Euclidean','Exponent',
[],'NumNeighbors', 10,'DistanceWeight', 'Squaredinverse','Standardize',
false);
            pred(CV,1)      = Model.predict(reference_preform(CV,1:end-
1));
        end

        unik_cutoff = unique(reference_minWL)';
        for cutoff = 1 : size(unik_cutoff,1)
            testset = find(reference_minWL <= unik_cutoff(cutoff));
            row = row+1;
            Out_data{row, 1} = wl1;
            Out_data{row, 2} = wl2;
            Out_data{row, 3} = size(find(reference_Label==0),2);
            Out_data{row, 4} = size(find(reference_Label==1),2);
            Out_data{row, 5} = size(reference_Label,2);
            Out_data{row, 6} = unik_cutoff(cutoff);
            Out_data{row, 7} = size(find(reference_Label(testset)==0),2);
            Out_data{row, 8} = size(find(reference_Label(testset)==1),2);
            Out_data{row, 9} = size(reference_Label(testset),2);
            Out_data{row,10} =
size(find( (pred(testset)~=reference_Label(testset)') &
(reference_Label(testset)==0)' ),1);
            Out_data{row,11} =
size(find( (pred(testset)~=reference_Label(testset)') &
(reference_Label(testset)==1)' ),1);
            Out_data{row,12} =
size(find(pred(testset)~=reference_Label(testset)'),1);
            Out_data{row,13} = Out_data{row,10} / Out_data{row,7};
            Out_data{row,14} = Out_data{row,11} / Out_data{row,8};
            Out_data{row,15} = Out_data{row,12} / Out_data{row,9};
        end
    end
end

All_Out_data = [];
Out_data_mat = cell2mat(Out_data(2:end,:));
unik_cutoff  = unique(Out_data_mat(:,6));
for cutoff = 1 : size(unik_cutoff,1)
    Out_data_cutoff =
Out_data_mat(find(Out_data_mat(:,6)==unik_cutoff(cutoff)),:);
    [u v]           = sort(Out_data_cutoff(:,end));
    All_Out_data    = [All_Out_data;
Out_data_cutoff(v(find(u==u(1))),:)];
end

KNN2_Wighted = [Out_data(1,:); num2cell(All_Out_data)];
save KNN2_Wighted.mat;
```

```
% SVM3_Linear
%-------------
clear all
clc
load CD_from_PCDDB.mat;
load CD_from_Papers.mat;
load CD_from_Own_measurements.mat;
alldata_WL = [175:250]';
alldata_CD = [CD_PCDDB CD_Papers_ CD_Own];
alldata_Label = [Label_PCDDB Label_Papers_ Label_Own];
alldata_serial = [1:size(alldata_Label,2)];

alldata_minWL=zeros(1,size(alldata_CD,2));
for i=1:size(alldata_CD,2)
    alldata_minWL(1,i) = min(find(~isnan(alldata_CD(:,i))))+174;
end

Out_data = cell(1,16);
Out_data = {'WL1','WL2','WL3',...
            'Ordered reference',...
            'Unordered reference',...
            'Total reference',...
            'Cutoff',...
            'Ordered test',...
            'Unordered test',...
            'Total test',...
            'Ordered ERROR',...
            'Unordere ERROR',...
            'Total ERROR',...
            'Ordered ERROR Ratio',...
            'Unordered ERROR Ratio',...
            'Total ERROR Ratio'};
row = 1;
for wl1 = 175 : 250
    for wl2 = wl1+3 : 250
        for wl3 = wl2+3 : 250
            tmp = find( ~isnan(alldata_CD(wl1-174,:)) &
~isnan(alldata_CD(wl2-174,:)) & ~isnan(alldata_CD(wl3-174,:)));
            reference_WL      = alldata_WL;
            reference_CD      = alldata_CD(:,tmp);
            reference_Label   = alldata_Label(:,tmp);
            reference_sorszam = alldata_sorszam(:,tmp);
            reference_minWL   = alldata_minWL(1,tmp);

            reference_preform = [reference_CD(wl1-174,:)'
reference_CD(wl2-174,:)' reference_CD(wl3-174,:)' reference_Label'];

            pred=[];
            for CV = 1 : size(reference_CD,2)
                reference       = reference_preform;
                reference(CV,:) = [];
                Model           = fitcsvm(reference(:,1:end-
1),reference(:,end),'Standardize',false,'KernelFunction','linear');
                pred(CV,1)      =
Model.predict(reference_preform(CV,1:end-1));
            end
```

```
            unik_cutoff = unique(reference_minWL)';
            for cutoff = 1 : size(unik_cutoff,1)
                testset = find(reference_minWL <= unik_cutoff(cutoff));
                row = row+1;
                Out_data{row, 1} = wl1;
                Out_data{row, 2} = wl2;
                Out_data{row, 3} = wl3;
                Out_data{row, 4} = size(find(reference_Label==0),2);
                Out_data{row, 5} = size(find(reference_Label==1),2);
                Out_data{row, 6} = size(reference_Label,2);
                Out_data{row, 7} = unik_cutoff(cutoff);
                Out_data{row, 8} =
size(find(reference_Label(testset)==0),2);
                Out_data{row, 9} =
size(find(reference_Label(testset)==1),2);
                Out_data{row,10} = size(reference_Label(testset),2);
                Out_data{row,11} =
size(find( (pred(testset)~=reference_Label(testset)') &
(reference_Label(testset)==0)' ),1);
                Out_data{row,12} =
size(find( (pred(testset)~=reference_Label(testset)') &
(reference_Label(testset)==1)' ),1);
                Out_data{row,13} =
size(find(pred(testset)~=reference_Label(testset)'),1);
                Out_data{row,14} = Out_data{row,11} / Out_data{row, 8};
                Out_data{row,15} = Out_data{row,12} / Out_data{row, 9};
                Out_data{row,16} = Out_data{row,13} / Out_data{row,10};
            end
        end
    end
end

All_Out_data = [];
Out_data_mat = cell2mat(Out_data(2:end,:));
unik_cutoff  = unique(Out_data_mat(:,7));
for cutoff = 1 : size(unik_cutoff,1)
    Out_data_cutoff =
Out_data_mat(find(Out_data_mat(:,7)==unik_cutoff(cutoff)),:);
    [u v]          = sort(Out_data_cutoff(:,end));
    All_Out_data    = [All_Out_data;
Out_data_cutoff(v(find(u==u(1))),:)];
end

SVM3_Linear = [Out_data(1,:); num2cell(All_Out_data)];
save SVM3_Linear.mat;

% SVM3_RBF
%----------
clear all
clc
load CD_from_PCDDB.mat;
load CD_from_Papers.mat;
load CD_from_Own_measurements.mat;
alldata_WL = [175:250]';
alldata_CD = [CD_PCDDB CD_Papers_ CD_Own];
alldata_Label = [Label_PCDDB Label_Papers_ Label_Own];
alldata_serial = [1:size(alldata_Label,2)];
```

```
alldata_minWL=zeros(1,size(alldata_CD,2));
for i=1:size(alldata_CD,2)
    alldata_minWL(1,i) = min(find(~isnan(alldata_CD(:,i))))+174;
end

Out_data = cell(1,16);
Out_data = {'WL1','WL2','WL3',...
            'Ordered reference',...
            'Unordered reference',...
            'Total reference',...
            'Cutoff',...
            'Ordered test',...
            'Unordered test',...
            'Total test',...
            'Ordered ERROR',...
            'Unordere ERROR',...
            'Total ERROR',...
            'Ordered ERROR Ratio',...
            'Unordered ERROR Ratio',...
            'Total ERROR Ratio'};
row = 1;
for wl1 = 175 : 250
    for wl2 = wl1+3 : 250
        for wl3 = wl2+3 : 250
            tmp = find( ~isnan(alldata_CD(wl1-174,:)) &
~isnan(alldata_CD(wl2-174,:)) & ~isnan(alldata_CD(wl3-174,:)));
            reference_WL      = alldata_WL;
            reference_CD      = alldata_CD(:,tmp);
            reference_Label   = alldata_Label(:,tmp);
            reference_sorszam = alldata_sorszam(:,tmp);
            reference_minWL   = alldata_minWL(1,tmp);

            reference_preform = [reference_CD(wl1-174,:)'
reference_CD(wl2-174,:)' reference_CD(wl3-174,:)' reference_Label'];

            pred=[];
            for CV = 1 : size(reference_CD,2)
                reference       = reference_preform;
                reference(CV,:) = [];
                Model           = fitcsvm(reference(:,1:end-
1),reference(:,end),'Standardize',false,'KernelFunction','rbf');
                pred(CV,1)      =
Model.predict(reference_preform(CV,1:end-1));
            end

            unik_cutoff = unique(reference_minWL)';
            for cutoff = 1 : size(unik_cutoff,1)
                testset = find(reference_minWL <= unik_cutoff(cutoff));
                row = row+1;
                Out_data{row, 1} = wl1;
                Out_data{row, 2} = wl2;
                Out_data{row, 3} = wl3;
                Out_data{row, 4} = size(find(reference_Label==0),2);
                Out_data{row, 5} = size(find(reference_Label==1),2);
                Out_data{row, 6} = size(reference_Label,2);
                Out_data{row, 7} = unik_cutoff(cutoff);
                Out_data{row, 8} =
size(find(reference_Label(testset)==0),2);
```

```
                Out_data{row, 9} =
size(find(reference_Label(testset)==1),2);
                Out_data{row,10} = size(reference_Label(testset),2);
                Out_data{row,11} =
size(find( (pred(testset)~=reference_Label(testset)') &
(reference_Label(testset)==0)' ),1);
                Out_data{row,12} =
size(find( (pred(testset)~=reference_Label(testset)') &
(reference_Label(testset)==1)' ),1);
                Out_data{row,13} =
size(find(pred(testset)~=reference_Label(testset)'),1);
                Out_data{row,14} = Out_data{row,11} / Out_data{row, 8};
                Out_data{row,15} = Out_data{row,12} / Out_data{row, 9};
                Out_data{row,16} = Out_data{row,13} / Out_data{row,10};
            end
        end
    end
end

All_Out_data = [];
Out_data_mat = cell2mat(Out_data(2:end,:));
unik_cutoff  = unique(Out_data_mat(:,7));
for cutoff = 1 : size(unik_cutoff,1)
    Out_data_cutoff =
Out_data_mat(find(Out_data_mat(:,7)==unik_cutoff(cutoff)),:);
    [u v]           = sort(Out_data_cutoff(:,end));
    All_Out_data    = [All_Out_data;
Out_data_cutoff(v(find(u==u(1))),:)];
end

SVM3_RBF = [Out_data(1,:); num2cell(All_Out_data)];
save SVM3_RBF.mat;

% Discr3_Linear
%---------------
clear all
clc
load CD_from_PCDDB.mat;
load CD_from_Papers.mat;
load CD_from_Own_measurements.mat;
alldata_WL = [175:250]';
alldata_CD = [CD_PCDDB CD_Papers_ CD_Own];
alldata_Label = [Label_PCDDB Label_Papers_ Label_Own];
alldata_serial = [1:size(alldata_Label,2)];

alldata_minWL=zeros(1,size(alldata_CD,2));
for i=1:size(alldata_CD,2)
    alldata_minWL(1,i) = min(find(~isnan(alldata_CD(:,i))))+174;
end

Out_data = cell(1,16);
Out_data = {'WL1','WL2','WL3',...
            'Ordered reference',...
            'Unordered reference',...
            'Total reference',...
            'Cutoff',...
            'Ordered test',...
            'Unordered test',...
```

```
            'Total test',...
            'Ordered ERROR',...
            'Unordere ERROR',...
            'Total ERROR',...
            'Ordered ERROR Ratio',...
            'Unordered ERROR Ratio',...
            'Total ERROR Ratio'};
row = 1;
for wl1 = 175 : 250
    for wl2 = wl1+3 : 250
        for wl3 = wl2+3 : 250
            tmp = find( ~isnan(alldata_CD(wl1-174,:)) &
~isnan(alldata_CD(wl2-174,:)) & ~isnan(alldata_CD(wl3-174,:)));
            reference_WL      = alldata_WL;
            reference_CD      = alldata_CD(:,tmp);
            reference_Label   = alldata_Label(:,tmp);
            reference_sorszam = alldata_sorszam(:,tmp);
            reference_minWL   = alldata_minWL(1,tmp);

            reference_preform = [reference_CD(wl1-174,:)'
reference_CD(wl2-174,:)' reference_CD(wl3-174,:)' reference_Label'];

            pred=[];
            for CV = 1 : size(reference_CD,2)
                reference       = reference_preform;
                reference(CV,:) = [];
                Model           = fitcdiscr(reference(:,1:end-
1),reference(:,end),'DiscrimType','linear');
                pred(CV,1)      =
Model.predict(reference_preform(CV,1:end-1));
            end

            unik_cutoff = unique(reference_minWL)';
            for cutoff = 1 : size(unik_cutoff,1)
                testset = find(reference_minWL <= unik_cutoff(cutoff));
                row = row+1;
                Out_data{row, 1} = wl1;
                Out_data{row, 2} = wl2;
                Out_data{row, 3} = wl3;
                Out_data{row, 4} = size(find(reference_Label==0),2);
                Out_data{row, 5} = size(find(reference_Label==1),2);
                Out_data{row, 6} = size(reference_Label,2);
                Out_data{row, 7} = unik_cutoff(cutoff);
                Out_data{row, 8} =
size(find(reference_Label(testset)==0),2);
                Out_data{row, 9} =
size(find(reference_Label(testset)==1),2);
                Out_data{row,10} = size(reference_Label(testset),2);
                Out_data{row,11} =
size(find( (pred(testset)~=reference_Label(testset)') &
(reference_Label(testset)==0)' ),1);
                Out_data{row,12} =
size(find( (pred(testset)~=reference_Label(testset)') &
(reference_Label(testset)==1)' ),1);
                Out_data{row,13} =
size(find(pred(testset)~=reference_Label(testset)'),1);
                Out_data{row,14} = Out_data{row,11} / Out_data{row, 8};
                Out_data{row,15} = Out_data{row,12} / Out_data{row, 9};
```

```
                Out_data{row,16} = Out_data{row,13} / Out_data{row,10};
            end
        end
    end
end

All_Out_data = [];
Out_data_mat = cell2mat(Out_data(2:end,:));
unik_cutoff  = unique(Out_data_mat(:,7));
for cutoff = 1 : size(unik_cutoff,1)
    Out_data_cutoff =
Out_data_mat(find(Out_data_mat(:,7)==unik_cutoff(cutoff)),:);
    [u v]           = sort(Out_data_cutoff(:,end));
    All_Out_data    = [All_Out_data;
Out_data_cutoff(v(find(u==u(1))),:)];
end

Discr3_Linear = [Out_data(1,:); num2cell(All_Out_data)];
save Discr3_Linear.mat;

% Discr3_Diaglinear
%-------------------
clear all
clc
load CD_from_PCDDB.mat;
load CD_from_Papers.mat;
load CD_from_Own_measurements.mat;
alldata_WL = [175:250]';
alldata_CD = [CD_PCDDB CD_Papers_ CD_Own];
alldata_Label = [Label_PCDDB Label_Papers_ Label_Own];
alldata_serial = [1:size(alldata_Label,2)];

alldata_minWL=zeros(1,size(alldata_CD,2));
for i=1:size(alldata_CD,2)
    alldata_minWL(1,i) = min(find(~isnan(alldata_CD(:,i))))+174;
end

Out_data = cell(1,16);
Out_data = {'WL1','WL2','WL3',...
            'Ordered reference',...
            'Unordered reference',...
            'Total reference',...
            'Cutoff',...
            'Ordered test',...
            'Unordered test',...
            'Total test',...
            'Ordered ERROR',...
            'Unordere ERROR',...
            'Total ERROR',...
            'Ordered ERROR Ratio',...
            'Unordered ERROR Ratio',...
            'Total ERROR Ratio'};
row = 1;
for wl1 = 175 : 250
    for wl2 = wl1+3 : 250
        for wl3 = wl2+3 : 250
            tmp = find( ~isnan(alldata_CD(wl1-174,:)) &
~isnan(alldata_CD(wl2-174,:)) & ~isnan(alldata_CD(wl3-174,:)));
```

```
            reference_WL      = alldata_WL;
            reference_CD      = alldata_CD(:,tmp);
            reference_Label   = alldata_Label(:,tmp);
            reference_sorszam = alldata_sorszam(:,tmp);
            reference_minWL   = alldata_minWL(1,tmp);

            reference_preform = [reference_CD(wl1-174,:)'
reference_CD(wl2-174,:)' reference_CD(wl3-174,:)' reference_Label'];

            pred=[];
            for CV = 1 : size(reference_CD,2)
                reference        = reference_preform;
                reference(CV,:) = [];
                Model            = fitcdiscr(reference(:,1:end-
1),reference(:,end),'DiscrimType','diaglinear');
                pred(CV,1)       =
Model.predict(reference_preform(CV,1:end-1));
            end

            unik_cutoff = unique(reference_minWL)';
            for cutoff = 1 : size(unik_cutoff,1)
                testset = find(reference_minWL <= unik_cutoff(cutoff));
                row = row+1;
                Out_data{row, 1} = wl1;
                Out_data{row, 2} = wl2;
                Out_data{row, 3} = wl3;
                Out_data{row, 4} = size(find(reference_Label==0),2);
                Out_data{row, 5} = size(find(reference_Label==1),2);
                Out_data{row, 6} = size(reference_Label,2);
                Out_data{row, 7} = unik_cutoff(cutoff);
                Out_data{row, 8} =
size(find(reference_Label(testset)==0),2);
                Out_data{row, 9} =
size(find(reference_Label(testset)==1),2);
                Out_data{row,10} = size(reference_Label(testset),2);
                Out_data{row,11} =
size(find( (pred(testset)~=reference_Label(testset)') &
(reference_Label(testset)==0)' ),1);
                Out_data{row,12} =
size(find( (pred(testset)~=reference_Label(testset)') &
(reference_Label(testset)==1)' ),1);
                Out_data{row,13} =
size(find(pred(testset)~=reference_Label(testset)'),1);
                Out_data{row,14} = Out_data{row,11} / Out_data{row, 8};
                Out_data{row,15} = Out_data{row,12} / Out_data{row, 9};
                Out_data{row,16} = Out_data{row,13} / Out_data{row,10};
            end
        end
    end
end

All_Out_data = [];
Out_data_mat = cell2mat(Out_data(2:end,:));
unik_cutoff  = unique(Out_data_mat(:,7));
for cutoff = 1 : size(unik_cutoff,1)
    Out_data_cutoff =
Out_data_mat(find(Out_data_mat(:,7)==unik_cutoff(cutoff)),:);
    [u v]           = sort(Out_data_cutoff(:,end));
```

```
    All_Out_data    = [All_Out_data;
Out_data_cutoff(v(find(u==u(1))),:)];
end

Discr3_Diaglinear = [Out_data(1,:); num2cell(All_Out_data)];
save Discr3_Diaglinear.mat;

% Discr3_Quadratic
%------------------
clear all
clc
load CD_from_PCDDB.mat;
load CD_from_Papers.mat;
load CD_from_Own_measurements.mat;
alldata_WL = [175:250]';
alldata_CD = [CD_PCDDB CD_Papers_ CD_Own];
alldata_Label = [Label_PCDDB Label_Papers_ Label_Own];
alldata_serial = [1:size(alldata_Label,2)];

alldata_minWL=zeros(1,size(alldata_CD,2));
for i=1:size(alldata_CD,2)
    alldata_minWL(1,i) = min(find(~isnan(alldata_CD(:,i))))+174;
end

Out_data = cell(1,16);
Out_data = {'WL1','WL2','WL3',...
            'Ordered reference',...
            'Unordered reference',...
            'Total reference',...
            'Cutoff',...
            'Ordered test',...
            'Unordered test',...
            'Total test',...
            'Ordered ERROR',...
            'Unordere ERROR',...
            'Total ERROR',...
            'Ordered ERROR Ratio',...
            'Unordered ERROR Ratio',...
            'Total ERROR Ratio'};
row = 1;
for wl1 = 175 : 250
    for wl2 = wl1+3 : 250
        for wl3 = wl2+3 : 250
            tmp = find( ~isnan(alldata_CD(wl1-174,:)) &
~isnan(alldata_CD(wl2-174,:)) & ~isnan(alldata_CD(wl3-174,:)));
            reference_WL      = alldata_WL;
            reference_CD      = alldata_CD(:,tmp);
            reference_Label   = alldata_Label(:,tmp);
            reference_sorszam = alldata_sorszam(:,tmp);
            reference_minWL   = alldata_minWL(1,tmp);

            reference_preform = [reference_CD(wl1-174,:)'
reference_CD(wl2-174,:)' reference_CD(wl3-174,:)' reference_Label'];

            pred=[];
            for CV = 1 : size(reference_CD,2)
                reference       = reference_preform;
                reference(CV,:) = [];
```

```
                Model           = fitcdiscr(reference(:,1:end-
1),reference(:,end),'DiscrimType','quadratic');
                pred(CV,1)      =
Model.predict(reference_preform(CV,1:end-1));
            end

            unik_cutoff = unique(reference_minWL)';
            for cutoff = 1 : size(unik_cutoff,1)
                testset = find(reference_minWL <= unik_cutoff(cutoff));
                row = row+1;
                Out_data{row, 1} = wl1;
                Out_data{row, 2} = wl2;
                Out_data{row, 3} = wl3;
                Out_data{row, 4} = size(find(reference_Label==0),2);
                Out_data{row, 5} = size(find(reference_Label==1),2);
                Out_data{row, 6} = size(reference_Label,2);
                Out_data{row, 7} = unik_cutoff(cutoff);
                Out_data{row, 8} =
size(find(reference_Label(testset)==0),2);
                Out_data{row, 9} =
size(find(reference_Label(testset)==1),2);
                Out_data{row,10} = size(reference_Label(testset),2);
                Out_data{row,11} =
size(find( (pred(testset)~=reference_Label(testset)') &
(reference_Label(testset)==0)' ),1);
                Out_data{row,12} =
size(find( (pred(testset)~=reference_Label(testset)') &
(reference_Label(testset)==1)' ),1);
                Out_data{row,13} =
size(find(pred(testset)~=reference_Label(testset)'),1);
                Out_data{row,14} = Out_data{row,11} / Out_data{row, 8};
                Out_data{row,15} = Out_data{row,12} / Out_data{row, 9};
                Out_data{row,16} = Out_data{row,13} / Out_data{row,10};
            end
        end
    end
end

All_Out_data = [];
Out_data_mat = cell2mat(Out_data(2:end,:));
unik_cutoff  = unique(Out_data_mat(:,7));
for cutoff = 1 : size(unik_cutoff,1)
    Out_data_cutoff =
Out_data_mat(find(Out_data_mat(:,7)==unik_cutoff(cutoff)),:);
    [u v]           = sort(Out_data_cutoff(:,end));
    All_Out_data    = [All_Out_data;
Out_data_cutoff(v(find(u==u(1))),:)];
end

Discr3_Quadratic = [Out_data(1,:); num2cell(All_Out_data)];
save Discr3_Quadratic.mat;

% Discr3_Diagquadratic
%----------------------
clear all
clc
load CD_from_PCDDB.mat;
load CD_from_Papers.mat;
```

```
load CD_from_Own_measurements.mat;
alldata_WL = [175:250]';
alldata_CD = [CD_PCDDB CD_Papers_ CD_Own];
alldata_Label = [Label_PCDDB Label_Papers_ Label_Own];
alldata_serial = [1:size(alldata_Label,2)];

alldata_minWL=zeros(1,size(alldata_CD,2));
for i=1:size(alldata_CD,2)
    alldata_minWL(1,i) = min(find(~isnan(alldata_CD(:,i))))+174;
end

Out_data = cell(1,16);
Out_data = {'WL1','WL2','WL3',...
            'Ordered reference',...
            'Unordered reference',...
            'Total reference',...
            'Cutoff',...
            'Ordered test',...
            'Unordered test',...
            'Total test',...
            'Ordered ERROR',...
            'Unordere ERROR',...
            'Total ERROR',...
            'Ordered ERROR Ratio',...
            'Unordered ERROR Ratio',...
            'Total ERROR Ratio'};
row = 1;
for wl1 = 175 : 250
    for wl2 = wl1+3 : 250
        for wl3 = wl2+3 : 250
            tmp = find( ~isnan(alldata_CD(wl1-174,:)) &
~isnan(alldata_CD(wl2-174,:)) & ~isnan(alldata_CD(wl3-174,:)));
            reference_WL      = alldata_WL;
            reference_CD      = alldata_CD(:,tmp);
            reference_Label   = alldata_Label(:,tmp);
            reference_sorszam = alldata_sorszam(:,tmp);
            reference_minWL   = alldata_minWL(1,tmp);

            reference_preform = [reference_CD(wl1-174,:)'
reference_CD(wl2-174,:)' reference_CD(wl3-174,:)' reference_Label'];

            pred=[];
            for CV = 1 : size(reference_CD,2)
                reference       = reference_preform;
                reference(CV,:) = [];
                Model           = fitcdiscr(reference(:,1:end-
1),reference(:,end),'DiscrimType','diagquadratic');
                pred(CV,1)      =
Model.predict(reference_preform(CV,1:end-1));
            end

            unik_cutoff = unique(reference_minWL)';
            for cutoff = 1 : size(unik_cutoff,1)
                testset = find(reference_minWL <= unik_cutoff(cutoff));
                row = row+1;
                Out_data{row, 1} = wl1;
                Out_data{row, 2} = wl2;
                Out_data{row, 3} = wl3;
```

```
                Out_data{row, 4} = size(find(reference_Label==0),2);
                Out_data{row, 5} = size(find(reference_Label==1),2);
                Out_data{row, 6} = size(reference_Label,2);
                Out_data{row, 7} = unik_cutoff(cutoff);
                Out_data{row, 8} =
size(find(reference_Label(testset)==0),2);
                Out_data{row, 9} =
size(find(reference_Label(testset)==1),2);
                Out_data{row,10} = size(reference_Label(testset),2);
                Out_data{row,11} =
size(find( (pred(testset)~=reference_Label(testset)') &
(reference_Label(testset)==0)' ),1);
                Out_data{row,12} =
size(find( (pred(testset)~=reference_Label(testset)') &
(reference_Label(testset)==1)' ),1);
                Out_data{row,13} =
size(find(pred(testset)~=reference_Label(testset)'),1);
                Out_data{row,14} = Out_data{row,11} / Out_data{row, 8};
                Out_data{row,15} = Out_data{row,12} / Out_data{row, 9};
                Out_data{row,16} = Out_data{row,13} / Out_data{row,10};
            end
        end
    end
end

All_Out_data = [];
Out_data_mat = cell2mat(Out_data(2:end,:));
unik_cutoff  = unique(Out_data_mat(:,7));
for cutoff = 1 : size(unik_cutoff,1)
    Out_data_cutoff =
Out_data_mat(find(Out_data_mat(:,7)==unik_cutoff(cutoff)),:);
    [u v]           = sort(Out_data_cutoff(:,end));
    All_Out_data    = [All_Out_data;
Out_data_cutoff(v(find(u==u(1))),:)];
end

Discr3_Diagquadratic = [Out_data(1,:); num2cell(All_Out_data)];
save Discr3_Diagquadratic.mat;

% Tree3_Simple
%--------------
clear all
clc
load CD_from_PCDDB.mat;
load CD_from_Papers.mat;
load CD_from_Own_measurements.mat;
alldata_WL = [175:250]';
alldata_CD = [CD_PCDDB CD_Papers_ CD_Own];
alldata_Label = [Label_PCDDB Label_Papers_ Label_Own];
alldata_serial = [1:size(alldata_Label,2)];

alldata_minWL=zeros(1,size(alldata_CD,2));
for i=1:size(alldata_CD,2)
    alldata_minWL(1,i) = min(find(~isnan(alldata_CD(:,i))))+174;
end

Out_data = cell(1,16);
Out_data = {'WL1','WL2','WL3',...
```

```
            'Ordered reference',...
            'Unordered reference',...
            'Total reference',...
            'Cutoff',...
            'Ordered test',...
            'Unordered test',...
            'Total test',...
            'Ordered ERROR',...
            'Unordere ERROR',...
            'Total ERROR',...
            'Ordered ERROR Ratio',...
            'Unordered ERROR Ratio',...
            'Total ERROR Ratio'};
row = 1;
for wl1 = 175 : 250
    for wl2 = wl1+3 : 250
        for wl3 = wl2+3 : 250
            tmp = find( ~isnan(alldata_CD(wl1-174,:)) &
~isnan(alldata_CD(wl2-174,:)) & ~isnan(alldata_CD(wl3-174,:)));
            reference_WL      = alldata_WL;
            reference_CD      = alldata_CD(:,tmp);
            reference_Label   = alldata_Label(:,tmp);
            reference_sorszam = alldata_sorszam(:,tmp);
            reference_minWL   = alldata_minWL(1,tmp);

            reference_preform = [reference_CD(wl1-174,:)'
reference_CD(wl2-174,:)' reference_CD(wl3-174,:)' reference_Label'];

            pred=[];
            for CV = 1 : size(reference_CD,2)
                reference       = reference_preform;
                reference(CV,:) = [];
                Model           = fitctree(reference(:,1:end-
1),reference(:,end),'MaxNumSplits', 4);
                pred(CV,1)      =
Model.predict(reference_preform(CV,1:end-1));
            end

            unik_cutoff = unique(reference_minWL)';
            for cutoff = 1 : size(unik_cutoff,1)
                testset = find(reference_minWL <= unik_cutoff(cutoff));
                row = row+1;
                Out_data{row, 1} = wl1;
                Out_data{row, 2} = wl2;
                Out_data{row, 3} = wl3;
                Out_data{row, 4} = size(find(reference_Label==0),2);
                Out_data{row, 5} = size(find(reference_Label==1),2);
                Out_data{row, 6} = size(reference_Label,2);
                Out_data{row, 7} = unik_cutoff(cutoff);
                Out_data{row, 8} =
size(find(reference_Label(testset)==0),2);
                Out_data{row, 9} =
size(find(reference_Label(testset)==1),2);
                Out_data{row,10} = size(reference_Label(testset),2);
                Out_data{row,11} =
size(find( (pred(testset)~=reference_Label(testset)') &
(reference_Label(testset)==0)' ),1);
```

```
                Out_data{row,12} =
size(find( (pred(testset)~=reference_Label(testset)') &
(reference_Label(testset)==1)' ),1);
                Out_data{row,13} =
size(find(pred(testset)~=reference_Label(testset)'),1);
                Out_data{row,14} = Out_data{row,11} / Out_data{row, 8};
                Out_data{row,15} = Out_data{row,12} / Out_data{row, 9};
                Out_data{row,16} = Out_data{row,13} / Out_data{row,10};
            end
        end
    end
end

All_Out_data = [];
Out_data_mat = cell2mat(Out_data(2:end,:));
unik_cutoff  = unique(Out_data_mat(:,7));
for cutoff = 1 : size(unik_cutoff,1)
    Out_data_cutoff =
Out_data_mat(find(Out_data_mat(:,7)==unik_cutoff(cutoff)),:);
    [u v]           = sort(Out_data_cutoff(:,end));
    All_Out_data    = [All_Out_data;
Out_data_cutoff(v(find(u==u(1))),:)];
end

Tree3_Simple = [Out_data(1,:); num2cell(All_Out_data)];
save Tree3_Simple.mat;

% Tree3_Medium
%--------------
clear all
clc
load CD_from_PCDDB.mat;
load CD_from_Papers.mat;
load CD_from_Own_measurements.mat;
alldata_WL = [175:250]';
alldata_CD = [CD_PCDDB CD_Papers_ CD_Own];
alldata_Label = [Label_PCDDB Label_Papers_ Label_Own];
alldata_serial = [1:size(alldata_Label,2)];

alldata_minWL=zeros(1,size(alldata_CD,2));
for i=1:size(alldata_CD,2)
    alldata_minWL(1,i) = min(find(~isnan(alldata_CD(:,i))))+174;
end

Out_data = cell(1,16);
Out_data = {'WL1','WL2','WL3',...
            'Ordered reference',...
            'Unordered reference',...
            'Total reference',...
            'Cutoff',...
            'Ordered test',...
            'Unordered test',...
            'Total test',...
            'Ordered ERROR',...
            'Unordere ERROR',...
            'Total ERROR',...
            'Ordered ERROR Ratio',...
            'Unordered ERROR Ratio',...
```

```
            'Total ERROR Ratio'};
row = 1;
for wl1 = 175 : 250
    for wl2 = wl1+3 : 250
        for wl3 = wl2+3 : 250
            tmp = find( ~isnan(alldata_CD(wl1-174,:)) &
~isnan(alldata_CD(wl2-174,:)) & ~isnan(alldata_CD(wl3-174,:)));
            reference_WL      = alldata_WL;
            reference_CD      = alldata_CD(:,tmp);
            reference_Label   = alldata_Label(:,tmp);
            reference_sorszam = alldata_sorszam(:,tmp);
            reference_minWL   = alldata_minWL(1,tmp);

            reference_preform = [reference_CD(wl1-174,:)'
reference_CD(wl2-174,:)' reference_CD(wl3-174,:)' reference_Label'];

            pred=[];
            for CV = 1 : size(reference_CD,2)
                reference       = reference_preform;
                reference(CV,:) = [];
                Model           = fitctree(reference(:,1:end-
1),reference(:,end),'MaxNumSplits', 20);
                pred(CV,1)      =
Model.predict(reference_preform(CV,1:end-1));
            end

            unik_cutoff = unique(reference_minWL)';
            for cutoff = 1 : size(unik_cutoff,1)
                testset = find(reference_minWL <= unik_cutoff(cutoff));
                row = row+1;
                Out_data{row, 1} = wl1;
                Out_data{row, 2} = wl2;
                Out_data{row, 3} = wl3;
                Out_data{row, 4} = size(find(reference_Label==0),2);
                Out_data{row, 5} = size(find(reference_Label==1),2);
                Out_data{row, 6} = size(reference_Label,2);
                Out_data{row, 7} = unik_cutoff(cutoff);
                Out_data{row, 8} =
size(find(reference_Label(testset)==0),2);
                Out_data{row, 9} =
size(find(reference_Label(testset)==1),2);
                Out_data{row,10} = size(reference_Label(testset),2);
                Out_data{row,11} =
size(find( (pred(testset)~=reference_Label(testset)') &
(reference_Label(testset)==0)' ),1);
                Out_data{row,12} =
size(find( (pred(testset)~=reference_Label(testset)') &
(reference_Label(testset)==1)' ),1);
                Out_data{row,13} =
size(find(pred(testset)~=reference_Label(testset)'),1);
                Out_data{row,14} = Out_data{row,11} / Out_data{row, 8};
                Out_data{row,15} = Out_data{row,12} / Out_data{row, 9};
                Out_data{row,16} = Out_data{row,13} / Out_data{row,10};
            end
        end
    end
end
```

```
All_Out_data = [];
Out_data_mat = cell2mat(Out_data(2:end,:));
unik_cutoff  = unique(Out_data_mat(:,7));
for cutoff = 1 : size(unik_cutoff,1)
    Out_data_cutoff =
Out_data_mat(find(Out_data_mat(:,7)==unik_cutoff(cutoff)),:);
    [u v]           = sort(Out_data_cutoff(:,end));
    All_Out_data    = [All_Out_data;
Out_data_cutoff(v(find(u==u(1))),:)];
end

Tree3_Medium = [Out_data(1,:); num2cell(All_Out_data)];
save Tree3_Medium.mat;

% Tree3_Complex
%---------------
clear all
clc
load CD_from_PCDDB.mat;
load CD_from_Papers.mat;
load CD_from_Own_measurements.mat;
alldata_WL = [175:250]';
alldata_CD = [CD_PCDDB CD_Papers_ CD_Own];
alldata_Label = [Label_PCDDB Label_Papers_ Label_Own];
alldata_serial = [1:size(alldata_Label,2)];

alldata_minWL=zeros(1,size(alldata_CD,2));
for i=1:size(alldata_CD,2)
    alldata_minWL(1,i) = min(find(~isnan(alldata_CD(:,i))))+174;
end

Out_data = cell(1,16);
Out_data = {'WL1','WL2','WL3',...
            'Ordered reference',...
            'Unordered reference',...
            'Total reference',...
            'Cutoff',...
            'Ordered test',...
            'Unordered test',...
            'Total test',...
            'Ordered ERROR',...
            'Unordere ERROR',...
            'Total ERROR',...
            'Ordered ERROR Ratio',...
            'Unordered ERROR Ratio',...
            'Total ERROR Ratio'};
row = 1;
for wl1 = 175 : 250
    for wl2 = wl1+3 : 250
        for wl3 = wl2+3 : 250
            tmp = find( ~isnan(alldata_CD(wl1-174,:)) &
~isnan(alldata_CD(wl2-174,:)) & ~isnan(alldata_CD(wl3-174,:)));
            reference_WL      = alldata_WL;
            reference_CD      = alldata_CD(:,tmp);
            reference_Label   = alldata_Label(:,tmp);
            reference_sorszam = alldata_sorszam(:,tmp);
            reference_minWL   = alldata_minWL(1,tmp);
```

```
            reference_preform = [reference_CD(wl1-174,:)'
reference_CD(wl2-174,:)' reference_CD(wl3-174,:)' reference_Label'];

            pred=[];
            for CV = 1 : size(reference_CD,2)
                reference       = reference_preform;
                reference(CV,:) = [];
                Model           = fitctree(reference(:,1:end-
1),reference(:,end),'MaxNumSplits', 100);
                pred(CV,1)      =
Model.predict(reference_preform(CV,1:end-1));
            end

            unik_cutoff = unique(reference_minWL)';
            for cutoff = 1 : size(unik_cutoff,1)
                testset = find(reference_minWL <= unik_cutoff(cutoff));
                row = row+1;
                Out_data{row, 1} = wl1;
                Out_data{row, 2} = wl2;
                Out_data{row, 3} = wl3;
                Out_data{row, 4} = size(find(reference_Label==0),2);
                Out_data{row, 5} = size(find(reference_Label==1),2);
                Out_data{row, 6} = size(reference_Label,2);
                Out_data{row, 7} = unik_cutoff(cutoff);
                Out_data{row, 8} =
size(find(reference_Label(testset)==0),2);
                Out_data{row, 9} =
size(find(reference_Label(testset)==1),2);
                Out_data{row,10} = size(reference_Label(testset),2);
                Out_data{row,11} =
size(find( (pred(testset)~=reference_Label(testset)') &
(reference_Label(testset)==0)' ),1);
                Out_data{row,12} =
size(find( (pred(testset)~=reference_Label(testset)') &
(reference_Label(testset)==1)' ),1);
                Out_data{row,13} =
size(find(pred(testset)~=reference_Label(testset)'),1);
                Out_data{row,14} = Out_data{row,11} / Out_data{row, 8};
                Out_data{row,15} = Out_data{row,12} / Out_data{row, 9};
                Out_data{row,16} = Out_data{row,13} / Out_data{row,10};
            end
        end
    end
end

All_Out_data = [];
Out_data_mat = cell2mat(Out_data(2:end,:));
unik_cutoff  = unique(Out_data_mat(:,7));
for cutoff = 1 : size(unik_cutoff,1)
    Out_data_cutoff =
Out_data_mat(find(Out_data_mat(:,7)==unik_cutoff(cutoff)),:);
    [u v]           = sort(Out_data_cutoff(:,end));
    All_Out_data    = [All_Out_data;
Out_data_cutoff(v(find(u==u(1))),:)];
end

Tree3_Complex = [Out_data(1,:); num2cell(All_Out_data)];
save Tree3_Complex.mat;
```

```
% KNN3_Fine
%-----------
clear all
clc
load CD_from_PCDDB.mat;
load CD_from_Papers.mat;
load CD_from_Own_measurements.mat;
alldata_WL = [175:250]';
alldata_CD = [CD_PCDDB CD_Papers_ CD_Own];
alldata_Label = [Label_PCDDB Label_Papers_ Label_Own];
alldata_serial = [1:size(alldata_Label,2)];

alldata_minWL=zeros(1,size(alldata_CD,2));
for i=1:size(alldata_CD,2)
    alldata_minWL(1,i) = min(find(~isnan(alldata_CD(:,i))))+174;
end

Out_data = cell(1,16);
Out_data = {'WL1','WL2','WL3',...
            'Ordered reference',...
            'Unordered reference',...
            'Total reference',...
            'Cutoff',...
            'Ordered test',...
            'Unordered test',...
            'Total test',...
            'Ordered ERROR',...
            'Unordere ERROR',...
            'Total ERROR',...
            'Ordered ERROR Ratio',...
            'Unordered ERROR Ratio',...
            'Total ERROR Ratio'};
row = 1;
for wl1 = 175 : 250
    for wl2 = wl1+3 : 250
        for wl3 = wl2+3 : 250
            tmp = find( ~isnan(alldata_CD(wl1-174,:)) &
~isnan(alldata_CD(wl2-174,:)) & ~isnan(alldata_CD(wl3-174,:)));
            reference_WL      = alldata_WL;
            reference_CD      = alldata_CD(:,tmp);
            reference_Label   = alldata_Label(:,tmp);
            reference_sorszam = alldata_sorszam(:,tmp);
            reference_minWL   = alldata_minWL(1,tmp);

            reference_preform = [reference_CD(wl1-174,:)'
reference_CD(wl2-174,:)' reference_CD(wl3-174,:)' reference_Label'];

            pred=[];
            for CV = 1 : size(reference_CD,2)
                reference       = reference_preform;
                reference(CV,:) = [];
                Model           = fitcknn(reference(:,1:end-
1),reference(:,end),'Distance', 'Euclidean','Exponent',
[],'NumNeighbors', 1,'DistanceWeight', 'Equal','Standardize', false);
                pred(CV,1)      =
Model.predict(reference_preform(CV,1:end-1));
            end
```

```
            unik_cutoff = unique(reference_minWL)';
            for cutoff = 1 : size(unik_cutoff,1)
                testset = find(reference_minWL <= unik_cutoff(cutoff));
                row = row+1;
                Out_data{row, 1} = wl1;
                Out_data{row, 2} = wl2;
                Out_data{row, 3} = wl3;
                Out_data{row, 4} = size(find(reference_Label==0),2);
                Out_data{row, 5} = size(find(reference_Label==1),2);
                Out_data{row, 6} = size(reference_Label,2);
                Out_data{row, 7} = unik_cutoff(cutoff);
                Out_data{row, 8} =
size(find(reference_Label(testset)==0),2);
                Out_data{row, 9} =
size(find(reference_Label(testset)==1),2);
                Out_data{row,10} = size(reference_Label(testset),2);
                Out_data{row,11} =
size(find( (pred(testset)~=reference_Label(testset)') &
(reference_Label(testset)==0)' ),1);
                Out_data{row,12} =
size(find( (pred(testset)~=reference_Label(testset)') &
(reference_Label(testset)==1)' ),1);
                Out_data{row,13} =
size(find(pred(testset)~=reference_Label(testset)'),1);
                Out_data{row,14} = Out_data{row,11} / Out_data{row, 8};
                Out_data{row,15} = Out_data{row,12} / Out_data{row, 9};
                Out_data{row,16} = Out_data{row,13} / Out_data{row,10};
            end
        end
    end
end

All_Out_data = [];
Out_data_mat = cell2mat(Out_data(2:end,:));
unik_cutoff  = unique(Out_data_mat(:,7));
for cutoff = 1 : size(unik_cutoff,1)
    Out_data_cutoff =
Out_data_mat(find(Out_data_mat(:,7)==unik_cutoff(cutoff)),:);
    [u v]           = sort(Out_data_cutoff(:,end));
    All_Out_data    = [All_Out_data;
Out_data_cutoff(v(find(u==u(1))),:)];
end

KNN3_Fine = [Out_data(1,:); num2cell(All_Out_data)];
save KNN3_Fine.mat;

% KNN3_Medium
%-------------
clear all
clc
load CD_from_PCDDB.mat;
load CD_from_Papers.mat;
load CD_from_Own_measurements.mat;
alldata_WL = [175:250]';
alldata_CD = [CD_PCDDB CD_Papers_ CD_Own];
alldata_Label = [Label_PCDDB Label_Papers_ Label_Own];
alldata_serial = [1:size(alldata_Label,2)];
```

```
alldata_minWL=zeros(1,size(alldata_CD,2));
for i=1:size(alldata_CD,2)
    alldata_minWL(1,i) = min(find(~isnan(alldata_CD(:,i))))+174;
end

Out_data = cell(1,16);
Out_data = {'WL1','WL2','WL3',...
            'Ordered reference',...
            'Unordered reference',...
            'Total reference',...
            'Cutoff',...
            'Ordered test',...
            'Unordered test',...
            'Total test',...
            'Ordered ERROR',...
            'Unordere ERROR',...
            'Total ERROR',...
            'Ordered ERROR Ratio',...
            'Unordered ERROR Ratio',...
            'Total ERROR Ratio'};
row = 1;
for wl1 = 175 : 250
    for wl2 = wl1+3 : 250
        for wl3 = wl2+3 : 250
            tmp = find( ~isnan(alldata_CD(wl1-174,:)) &
~isnan(alldata_CD(wl2-174,:)) & ~isnan(alldata_CD(wl3-174,:)));
            reference_WL      = alldata_WL;
            reference_CD      = alldata_CD(:,tmp);
            reference_Label   = alldata_Label(:,tmp);
            reference_sorszam = alldata_sorszam(:,tmp);
            reference_minWL   = alldata_minWL(1,tmp);

            reference_preform = [reference_CD(wl1-174,:)'
reference_CD(wl2-174,:)' reference_CD(wl3-174,:)' reference_Label'];

            pred=[];
            for CV = 1 : size(reference_CD,2)
                reference       = reference_preform;
                reference(CV,:) = [];
                Model           = fitcknn(reference(:,1:end-
1),reference(:,end),'Distance', 'Euclidean','Exponent',
[],'NumNeighbors', 10,'DistanceWeight', 'Equal','Standardize', false);
                pred(CV,1)      =
Model.predict(reference_preform(CV,1:end-1));
            end

            unik_cutoff = unique(reference_minWL)';
            for cutoff = 1 : size(unik_cutoff,1)
                testset = find(reference_minWL <= unik_cutoff(cutoff));
                row = row+1;
                Out_data{row, 1} = wl1;
                Out_data{row, 2} = wl2;
                Out_data{row, 3} = wl3;
                Out_data{row, 4} = size(find(reference_Label==0),2);
                Out_data{row, 5} = size(find(reference_Label==1),2);
                Out_data{row, 6} = size(reference_Label,2);
                Out_data{row, 7} = unik_cutoff(cutoff);
```

```
                Out_data{row, 8} =
size(find(reference_Label(testset)==0),2);
                Out_data{row, 9} =
size(find(reference_Label(testset)==1),2);
                Out_data{row,10} = size(reference_Label(testset),2);
                Out_data{row,11} =
size(find( (pred(testset)~=reference_Label(testset)') &
(reference_Label(testset)==0)' ),1);
                Out_data{row,12} =
size(find( (pred(testset)~=reference_Label(testset)') &
(reference_Label(testset)==1)' ),1);
                Out_data{row,13} =
size(find(pred(testset)~=reference_Label(testset)'),1);
                Out_data{row,14} = Out_data{row,11} / Out_data{row, 8};
                Out_data{row,15} = Out_data{row,12} / Out_data{row, 9};
                Out_data{row,16} = Out_data{row,13} / Out_data{row,10};
            end
        end
    end
end

All_Out_data = [];
Out_data_mat = cell2mat(Out_data(2:end,:));
unik_cutoff  = unique(Out_data_mat(:,7));
for cutoff = 1 : size(unik_cutoff,1)
    Out_data_cutoff =
Out_data_mat(find(Out_data_mat(:,7)==unik_cutoff(cutoff)),:);
    [u v]           = sort(Out_data_cutoff(:,end));
    All_Out_data    = [All_Out_data;
Out_data_cutoff(v(find(u==u(1))),:)];
end

KNN3_Medium = [Out_data(1,:); num2cell(All_Out_data)];
save KNN3_Medium.mat;

% KNN3_Coarse
%-------------
clear all
clc
load CD_from_PCDDB.mat;
load CD_from_Papers.mat;
load CD_from_Own_measurements.mat;
alldata_WL = [175:250]';
alldata_CD = [CD_PCDDB CD_Papers_ CD_Own];
alldata_Label = [Label_PCDDB Label_Papers_ Label_Own];
alldata_serial = [1:size(alldata_Label,2)];

alldata_minWL=zeros(1,size(alldata_CD,2));
for i=1:size(alldata_CD,2)
    alldata_minWL(1,i) = min(find(~isnan(alldata_CD(:,i))))+174;
end

Out_data = cell(1,16);
Out_data = {'WL1','WL2','WL3',...
            'Ordered reference',...
            'Unordered reference',...
            'Total reference',...
            'Cutoff',...
```

```
            'Ordered test',...
            'Unordered test',...
            'Total test',...
            'Ordered ERROR',...
            'Unordere ERROR',...
            'Total ERROR',...
            'Ordered ERROR Ratio',...
            'Unordered ERROR Ratio',...
            'Total ERROR Ratio'};
row = 1;
for wl1 = 175 : 250
    for wl2 = wl1+3 : 250
        for wl3 = wl2+3 : 250
            tmp = find( ~isnan(alldata_CD(wl1-174,:)) &
~isnan(alldata_CD(wl2-174,:)) & ~isnan(alldata_CD(wl3-174,:)));
            reference_WL      = alldata_WL;
            reference_CD      = alldata_CD(:,tmp);
            reference_Label   = alldata_Label(:,tmp);
            reference_sorszam = alldata_sorszam(:,tmp);
            reference_minWL   = alldata_minWL(1,tmp);

            reference_preform = [reference_CD(wl1-174,:)'
reference_CD(wl2-174,:)' reference_CD(wl3-174,:)' reference_Label'];

            pred=[];
            for CV = 1 : size(reference_CD,2)
                reference       = reference_preform;
                reference(CV,:) = [];
                Model           = fitcknn(reference(:,1:end-
1),reference(:,end),'Distance', 'Euclidean','Exponent',
[],'NumNeighbors', 100,'DistanceWeight', 'Equal','Standardize', false);
                pred(CV,1)      =
Model.predict(reference_preform(CV,1:end-1));
            end

            unik_cutoff = unique(reference_minWL)';
            for cutoff = 1 : size(unik_cutoff,1)
                testset = find(reference_minWL <= unik_cutoff(cutoff));
                row = row+1;
                Out_data{row, 1} = wl1;
                Out_data{row, 2} = wl2;
                Out_data{row, 3} = wl3;
                Out_data{row, 4} = size(find(reference_Label==0),2);
                Out_data{row, 5} = size(find(reference_Label==1),2);
                Out_data{row, 6} = size(reference_Label,2);
                Out_data{row, 7} = unik_cutoff(cutoff);
                Out_data{row, 8} =
size(find(reference_Label(testset)==0),2);
                Out_data{row, 9} =
size(find(reference_Label(testset)==1),2);
                Out_data{row,10} = size(reference_Label(testset),2);
                Out_data{row,11} =
size(find( (pred(testset)~=reference_Label(testset)') &
(reference_Label(testset)==0)' ),1);
                Out_data{row,12} =
size(find( (pred(testset)~=reference_Label(testset)') &
(reference_Label(testset)==1)' ),1);
```

```
                Out_data{row,13} =
size(find(pred(testset)~=reference_Label(testset)'),1);
                Out_data{row,14} = Out_data{row,11} / Out_data{row, 8};
                Out_data{row,15} = Out_data{row,12} / Out_data{row, 9};
                Out_data{row,16} = Out_data{row,13} / Out_data{row,10};
            end
        end
    end
end

All_Out_data = [];
Out_data_mat = cell2mat(Out_data(2:end,:));
unik_cutoff  = unique(Out_data_mat(:,7));
for cutoff = 1 : size(unik_cutoff,1)
    Out_data_cutoff =
Out_data_mat(find(Out_data_mat(:,7)==unik_cutoff(cutoff)),:);
    [u v]           = sort(Out_data_cutoff(:,end));
    All_Out_data    = [All_Out_data;
Out_data_cutoff(v(find(u==u(1))),:)];
end

KNN3_Coarse = [Out_data(1,:); num2cell(All_Out_data)];
save KNN3_Coarse.mat;

% KNN3_Cosine
%-------------
clear all
clc
load CD_from_PCDDB.mat;
load CD_from_Papers.mat;
load CD_from_Own_measurements.mat;
alldata_WL = [175:250]';
alldata_CD = [CD_PCDDB CD_Papers_ CD_Own];
alldata_Label = [Label_PCDDB Label_Papers_ Label_Own];
alldata_serial = [1:size(alldata_Label,2)];

alldata_minWL=zeros(1,size(alldata_CD,2));
for i=1:size(alldata_CD,2)
    alldata_minWL(1,i) = min(find(~isnan(alldata_CD(:,i))))+174;
end

Out_data = cell(1,16);
Out_data = {'WL1','WL2','WL3',...
            'Ordered reference',...
            'Unordered reference',...
            'Total reference',...
            'Cutoff',...
            'Ordered test',...
            'Unordered test',...
            'Total test',...
            'Ordered ERROR',...
            'Unordere ERROR',...
            'Total ERROR',...
            'Ordered ERROR Ratio',...
            'Unordered ERROR Ratio',...
            'Total ERROR Ratio'};
row = 1;
for wl1 = 175 : 250
```

```
    for wl2 = wl1+3 : 250
        for wl3 = wl2+3 : 250
            tmp = find( ~isnan(alldata_CD(wl1-174,:)) &
~isnan(alldata_CD(wl2-174,:)) & ~isnan(alldata_CD(wl3-174,:)));
            reference_WL      = alldata_WL;
            reference_CD      = alldata_CD(:,tmp);
            reference_Label   = alldata_Label(:,tmp);
            reference_sorszam = alldata_sorszam(:,tmp);
            reference_minWL   = alldata_minWL(1,tmp);

            reference_preform = [reference_CD(wl1-174,:)'
reference_CD(wl2-174,:)' reference_CD(wl3-174,:)' reference_Label'];

            pred=[];
            for CV = 1 : size(reference_CD,2)
                reference       = reference_preform;
                reference(CV,:) = [];
                Model           = fitcknn(reference(:,1:end-
1),reference(:,end),'Distance', 'Cosine','Exponent', [],'NumNeighbors',
10,'DistanceWeight', 'Equal','Standardize', false);
                pred(CV,1)      =
Model.predict(reference_preform(CV,1:end-1));
            end

            unik_cutoff = unique(reference_minWL)';
            for cutoff = 1 : size(unik_cutoff,1)
                testset = find(reference_minWL <= unik_cutoff(cutoff));
                row = row+1;
                Out_data{row, 1} = wl1;
                Out_data{row, 2} = wl2;
                Out_data{row, 3} = wl3;
                Out_data{row, 4} = size(find(reference_Label==0),2);
                Out_data{row, 5} = size(find(reference_Label==1),2);
                Out_data{row, 6} = size(reference_Label,2);
                Out_data{row, 7} = unik_cutoff(cutoff);
                Out_data{row, 8} =
size(find(reference_Label(testset)==0),2);
                Out_data{row, 9} =
size(find(reference_Label(testset)==1),2);
                Out_data{row,10} = size(reference_Label(testset),2);
                Out_data{row,11} =
size(find( (pred(testset)~=reference_Label(testset)') &
(reference_Label(testset)==0)' ),1);
                Out_data{row,12} =
size(find( (pred(testset)~=reference_Label(testset)') &
(reference_Label(testset)==1)' ),1);
                Out_data{row,13} =
size(find(pred(testset)~=reference_Label(testset)'),1);
                Out_data{row,14} = Out_data{row,11} / Out_data{row, 8};
                Out_data{row,15} = Out_data{row,12} / Out_data{row, 9};
                Out_data{row,16} = Out_data{row,13} / Out_data{row,10};
            end
        end
    end
end

All_Out_data = [];
Out_data_mat = cell2mat(Out_data(2:end,:));
```

```
unik_cutoff  = unique(Out_data_mat(:,7));
for cutoff = 1 : size(unik_cutoff,1)
    Out_data_cutoff =
Out_data_mat(find(Out_data_mat(:,7)==unik_cutoff(cutoff)),:);
    [u v]           = sort(Out_data_cutoff(:,end));
    All_Out_data    = [All_Out_data;
Out_data_cutoff(v(find(u==u(1))),:)];
end

KNN3_Cosine = [Out_data(1,:); num2cell(All_Out_data)];
save KNN3_Cosine.mat;

% KNN3_Wighted
%--------------
clear all
clc
load CD_from_PCDDB.mat;
load CD_from_Papers.mat;
load CD_from_Own_measurements.mat;
alldata_WL = [175:250]';
alldata_CD = [CD_PCDDB CD_Papers_ CD_Own];
alldata_Label = [Label_PCDDB Label_Papers_ Label_Own];
alldata_serial = [1:size(alldata_Label,2)];

alldata_minWL=zeros(1,size(alldata_CD,2));
for i=1:size(alldata_CD,2)
    alldata_minWL(1,i) = min(find(~isnan(alldata_CD(:,i))))+174;
end

Out_data = cell(1,16);
Out_data = {'WL1','WL2','WL3',...
            'Ordered reference',...
            'Unordered reference',...
            'Total reference',...
            'Cutoff',...
            'Ordered test',...
            'Unordered test',...
            'Total test',...
            'Ordered ERROR',...
            'Unordere ERROR',...
            'Total ERROR',...
            'Ordered ERROR Ratio',...
            'Unordered ERROR Ratio',...
            'Total ERROR Ratio'};
row = 1;
for wl1 = 175 : 250
    for wl2 = wl1+3 : 250
        for wl3 = wl2+3 : 250
            tmp = find( ~isnan(alldata_CD(wl1-174,:)) &
~isnan(alldata_CD(wl2-174,:)) & ~isnan(alldata_CD(wl3-174,:)));
            reference_WL      = alldata_WL;
            reference_CD      = alldata_CD(:,tmp);
            reference_Label   = alldata_Label(:,tmp);
            reference_sorszam = alldata_sorszam(:,tmp);
            reference_minWL   = alldata_minWL(1,tmp);

            reference_preform = [reference_CD(wl1-174,:)'
reference_CD(wl2-174,:)' reference_CD(wl3-174,:)' reference_Label'];
```

```
            pred=[];
            for CV = 1 : size(reference_CD,2)
                reference       = reference_preform;
                reference(CV,:) = [];
                Model           = fitcknn(reference(:,1:end-
1),reference(:,end),'Distance', 'Euclidean','Exponent',
[],'NumNeighbors', 10,'DistanceWeight', 'Squaredinverse','Standardize',
false);
                pred(CV,1)      =
Model.predict(reference_preform(CV,1:end-1));
            end

            unik_cutoff = unique(reference_minWL)';
            for cutoff = 1 : size(unik_cutoff,1)
                testset = find(reference_minWL <= unik_cutoff(cutoff));
                row = row+1;
                Out_data{row, 1} = wl1;
                Out_data{row, 2} = wl2;
                Out_data{row, 3} = wl3;
                Out_data{row, 4} = size(find(reference_Label==0),2);
                Out_data{row, 5} = size(find(reference_Label==1),2);
                Out_data{row, 6} = size(reference_Label,2);
                Out_data{row, 7} = unik_cutoff(cutoff);
                Out_data{row, 8} =
size(find(reference_Label(testset)==0),2);
                Out_data{row, 9} =
size(find(reference_Label(testset)==1),2);
                Out_data{row,10} = size(reference_Label(testset),2);
                Out_data{row,11} =
size(find( (pred(testset)~=reference_Label(testset)') &
(reference_Label(testset)==0)' ),1);
                Out_data{row,12} =
size(find( (pred(testset)~=reference_Label(testset)') &
(reference_Label(testset)==1)' ),1);
                Out_data{row,13} =
size(find(pred(testset)~=reference_Label(testset)'),1);
                Out_data{row,14} = Out_data{row,11} / Out_data{row, 8};
                Out_data{row,15} = Out_data{row,12} / Out_data{row, 9};
                Out_data{row,16} = Out_data{row,13} / Out_data{row,10};
            end
        end
    end
end

All_Out_data = [];
Out_data_mat = cell2mat(Out_data(2:end,:));
unik_cutoff  = unique(Out_data_mat(:,7));
for cutoff = 1 : size(unik_cutoff,1)
    Out_data_cutoff =
Out_data_mat(find(Out_data_mat(:,7)==unik_cutoff(cutoff)),:);
    [u v]           = sort(Out_data_cutoff(:,end));
    All_Out_data    = [All_Out_data;
Out_data_cutoff(v(find(u==u(1))),:)];
end

KNN3_Wighted = [Out_data(1,:); num2cell(All_Out_data)];
save KNN3_Wighted.mat;
```
