# Supplementary material for: Disordered–Ordered Protein Binary Classification by Circular Dichroism Spectroscopy
Source: Front Mol Biosci. 2022 May 3;9:863141. doi: 10.3389/fmolb.2022.863141 (PMC9110821; doi:10.3389/fmolb.2022.863141)
Supplement: Supplementary file 3 [file DataSheet1.PDF]

# *Supplementary Material*

## Disordered-ordered protein binary classification by circular dichroism spectroscopy

András Micsonai, Éva Moussong, Nikoletta Murvai, Ágnes Tantos, Orsolya Tőke, Matthieu Réfrégiers, Frank Wien, and József Kardos

Correspondence: kardos@elte.hu

**Supplementary Table 1.** List of proteins studied in this work

| Protein name | Source [a] | Disordered/ ordered | Wavelength cutoff (nm) |
|---|---|---|---|
| Aldolase | PCDDB/CD0000001000 | Ord | 175 |
| Alkaline phosphatase | PCDDB/CD0000002000 | Ord | 175 |
| Alpha amylase | PCDDB/CD0000003000 | Ord | 175 |
| Alpha bungarotoxin | PCDDB/CD0000004000 | Ord | 175 |
| Alpha chymotrypsin | PCDDB/CD0000005000 | Ord | 175 |
| Alpha chymotrypsinogen | PCDDB/CD0000006000 | Ord | 175 |
| Aprotinin | PCDDB/CD0000007000 | Ord | 175 |
| Avidin | PCDDB/CD0000008000 | Ord | 175 |
| Beta amylase | PCDDB/CD0000009000 | Ord | 175 |
| Beta galactosidase | PCDDB/CD0000010000 | Ord | 175 |
| Beta lactoglobulin | PCDDB/CD0000011000 | Ord | 175 |
| C-phycocyanin | PCDDB/CD0000012000 | Ord | 175 |
| Calmodulin | PCDDB/CD0000013000 | Ord | 175 |
| Carbonic anhydrase I | PCDDB/CD0000014000 | Ord | 175 |
| Carbonic anhydrase II | PCDDB/CD0000015000 | Ord | 175 |
| Carboxypeptidase A1 | PCDDB/CD0000016000 | Ord | 175 |
| Catalase | PCDDB/CD0000017000 | Ord | 175 |
| Ceruloplasmin | PCDDB/CD0000018000 | Ord | 175 |
| Citrate synthase | PCDDB/CD0000019000 | Ord | 175 |
| Concanavalin-A | PCDDB/CD0000020000 | Ord | 175 |
| Cytochrome c | PCDDB/CD0000021000 | Ord | 175 |
| Beta-crystallin B2 | PCDDB/CD0000022000 | Ord | 175 |
| Gamma-crystallin B | PCDDB/CD0000023000 | Ord | 175 |
| Gamma-crystallin D | PCDDB/CD0000024000 | Ord | 175 |
| Gamma-crystallin E | PCDDB/CD0000025000 | Ord | 175 |

| | | | |
|---|---|---|---|
| Gamma-crystallin S, C terminus | PCDDB/CD0000026000 | Ord | 175 |
| Gamma-crystallin D | PCDDB/CD0000027000 | Ord | 175 |
| 3-dehydroquinate dehydratase (*Salmonella typhi*) | PCDDB/CD0000028000 | Ord | 175 |
| 3-dehydroquinate dehydratase (*Mycobacterium tuberculosis*) | PCDDB/CD0000029000 | Ord | 175 |
| Deoxyribonuclease-1 | PCDDB/CD0000030000 | Ord | 175 |
| Elastase | PCDDB/CD0000031000 | Ord | 175 |
| Ferredoxin | PCDDB/CD0000032000 | Ord | 175 |
| Glucose oxidase | PCDDB/CD0000033000 | Ord | 175 |
| Glutamate dehydrogenase I | PCDDB/CD0000034000 | Ord | 175 |
| Glycogen phosphorylase-b | PCDDB/CD0000035000 | Ord | 175 |
| Haloalkane dehalogenase | PCDDB/CD0000036000 | Ord | 175 |
| Hemoglobin | PCDDB/CD0000037000 | Ord | 175 |
| Human serum albumin | PCDDB/CD0000038000 | Ord | 175 |
| Immunoglobulin G | PCDDB/CD0000039000 | Ord | 175 |
| Insulin | PCDDB/CD0000040000 | Ord | 175 |
| Jacalin | PCDDB/CD0000041000 | Ord | 175 |
| Lactoferrin | PCDDB/CD0000042000 | Ord | 175 |
| Lectin (*Lens culinaris*) | PCDDB/CD0000043000 | Ord | 175 |
| Leptin | PCDDB/CD0000044000 | Ord | 175 |
| Lysozyme (*Gallus gallus*) | PCDDB/CD0000045000 | Ord | 175 |
| Monellin | PCDDB/CD0000046000 | Ord | 175 |
| Myoglobin (*Equus caballus*) | PCDDB/CD0000047000 | Ord | 175 |
| Myoglobin (*Physeter catodon*) | PCDDB/CD0000048000 | Ord | 175 |
| Nitrogen metabolite repression regulator | PCDDB/CD0000049000 | Ord | 175 |
| Ovalbumin | PCDDB/CD0000050000 | Ord | 175 |
| Ovotransferrin | PCDDB/CD0000051000 | Ord | 175 |
| Papain | PCDDB/CD0000052000 | Ord | 175 |
| Lectin (*Pisum sativum*) | PCDDB/CD0000053000 | Ord | 175 |
| Pectate lyase C (*Dickeya chrysanthemi*) | PCDDB/CD0000054000 | Ord | 175 |
| Pepsinogen | PCDDB/CD0000055000 | Ord | 175 |
| Peroxidase C1 | PCDDB/CD0000056000 | Ord | 175 |
| Phosphoglucomutase-1 | PCDDB/CD0000057000 | Ord | 175 |
| Phosphoglycerate kinase | PCDDB/CD0000058000 | Ord | 175 |
| Phospholipase A2 | PCDDB/CD0000059000 | Ord | 175 |
| Phenylethanolamine N-methyltransferase | PCDDB/CD0000060000 | Ord | 175 |
| Pyruvate kinase | PCDDB/CD0000061000 | Ord | 175 |
| Rhodanese | PCDDB/CD0000062000 | Ord | 175 |

| | | | |
|---|---|---|---|
| Ribonuclease, pancreatic | PCDDB/CD0000063000 | Ord | 175 |
| Rubredoxin | PCDDB/CD0000064000 | Ord | 175 |
| Trypsin inhibitor A | PCDDB/CD0000065000 | Ord | 175 |
| Streptavidin | PCDDB/CD0000066000 | Ord | 175 |
| Subtilisin Carlsberg | PCDDB/CD0000067000 | Ord | 175 |
| Superoxide dismutase [Cu-Zn] | PCDDB/CD0000068000 | Ord | 175 |
| Thaumatin I | PCDDB/CD0000069000 | Ord | 175 |
| Triose phosphate isomerase | PCDDB/CD0000070000 | Ord | 175 |
| Ubiquitin | PCDDB/CD0000071000 | Ord | 175 |
| Alpha-lactalbumin | PCDDB/CD0000072000 | Ord | 175 |
| Amyloglucosidase | PCDDB/CD0000073000 | Ord | 175 |
| Aspartate-beta-semialdehyde dehydrogenase | PCDDB/CD0000074000 | Ord | 175 |
| Aspartokinase 3 | PCDDB/CD0000075000 | Ord | 175 |
| Azurin | PCDDB/CD0000076000 | Ord | 175 |
| Bence-Jones protein | PCDDB/CD0000077000 | Ord | 175 |
| Bowman-Birk proteinase inhibitor | PCDDB/CD0000078000 | Ord | 175 |
| Dihydrofolate reductase | PCDDB/CD0000079000 | Ord | 175 |
| 3-dehydroquinate dehydratase (*Staphylococcus aureus*) | PCDDB/CD0000080000 | Ord | 175 |
| 3-dehydroquinate synthase (*Staphylococcus aureus*) | PCDDB/CD0000081000 | Ord | 175 |
| Ferretin light chain | PCDDB/CD0000082000 | Ord | 173 |
| Guanylate kinase | PCDDB/CD0000083000 | Ord | 175 |
| RNA-binding protein Hfq | PCDDB/CD0000084000 | Ord | 175 |
| Lysine-specific demethylase 4A | PCDDB/CD0000085000 | Ord | 177 |
| Latexin | PCDDB/CD0000086000 | Ord | 175 |
| Thermonuclease | PCDDB/CD0000087000 | Ord | 175 |
| ECE zinc metalloprotease | PCDDB/CD0000088000 | Ord | 173 |
| Neuraminidase | PCDDB/CD0000089000 | Ord | 175 |
| Pectate lyase (*Bacillus subtilis*) | PCDDB/CD0000090000 | Ord | 180 |
| Prealbumin | PCDDB/CD0000091000 | Ord | 180 |
| STE20-like kinase | PCDDB/CD0000092000 | Ord | 175 |
| Mannose-specific lectin | PCDDB/CD0000093000 | Ord | 175 |
| Serotransferrin | PCDDB/CD0000094000 | Ord | 175 |
| Apo-serotransferrin | PCDDB/CD0000095000 | Ord | 175 |
| Beta-trypsin | PCDDB/CD0000096000 | Ord | 175 |
| Agglutinin isolectin 2 | PCDDB/CD0000097000 | Ord | 170 |
| Resuscitation-promoting factor | PCDDB/CD0000098000 | Ord | 175 |
| Ammonia channel | PCDDB/CD0000099000 | Ord | 170 |

| | | | |
|---|---|---|---|
| Acridine resistance protein B | PCDDB/CD0000100000 | Ord | 173 |
| Bacteriorhodopsin (*Halobacterium salinarum*) | PCDDB/CD0000101000 | Ord | 173 |
| Vitamin B12 transporter BtuB | PCDDB/CD0000102000 | Ord | 178 |
| Vitamin B12 import system permease protein BtuC | PCDDB/CD0000103000 | Ord | 176 |
| H(+)/Cl(-) exchange transporter ClcA | PCDDB/CD0000104000 | Ord | 180 |
| Cytochrome b-c1 | PCDDB/CD0000105000 | Ord | 175 |
| Cytochrome c oxidase | PCDDB/CD0000106000 | Ord | 175 |
| Ferrienterobactin receptor | PCDDB/CD0000107000 | Ord | 180 |
| Ferrichrome-iron receptor | PCDDB/CD0000108000 | Ord | 173 |
| Rhomboid protease glpG | PCDDB/CD0000109000 | Ord | 180 |
| Voltage-gated potassium channel | PCDDB/CD0000110000 | Ord | 180 |
| ATP-sensitive inwardly rectifying potassium channel | PCDDB/CD0000111000 | Ord | 180 |
| Lactose permease | PCDDB/CD0000112000 | Ord | 172 |
| Na(+):neurotransmitter symporter (Snf family) | PCDDB/CD0000113000 | Ord | 180 |
| Light harvesting protein | PCDDB/CD0000114000 | Ord | 180 |
| Large-conductance mechanosensitive channel | PCDDB/CD0000115000 | Ord | 175 |
| NalP | PCDDB/CD0000116000 | Ord | 180 |
| Sensory rhodopsin-2 (*Natronomonas pharaonis*) | PCDDB/CD0000117000 | Ord | 175 |
| Outer membrane protein G (*Escherichia coli*) | PCDDB/CD0000118000 | Ord | 173 |
| Outer membrane protein (*Neisseria meningitidis*) | PCDDB/CD0000119000 | Ord | 175 |
| TraF protein | PCDDB/CD0000120000 | Ord | 180 |
| Reaction center protein (*Cereibacter sphaeroides*) | PCDDB/CD0000121000 | Ord | 175 |
| Reaction center protein (*Blastochloris viridis*) | PCDDB/CD0000122000 | Ord | 173 |
| Rhodopsin (*Bos taurus*) | PCDDB/CD0000123000 | Ord | 172 |
| Preprotein translocase subunit secY | PCDDB/CD0000124000 | Ord | 176 |
| Sarcoplasmic/endoplasmic reticulum calcium ATPase 1 | PCDDB/CD0000125000 | Ord | 180 |
| Succinate dehydrogenase | PCDDB/CD0000126000 | Ord | 171 |
| Sucrose porin | PCDDB/CD0000127000 | Ord | 175 |
| Outer membrane lipoprotein Wza | PCDDB/CD0000128000 | Ord | 180 |
| Beta-insect excitatory toxin Bj-xtrIT | PCDDB/CD0004244000 | Ord | 175 |
| Ion transport protein, pore | PCDDB/CD0006226000 | Ord | 175 |
| Ion transport protein, full length, wild type | PCDDB/CD0006224000 | Ord | 180 |

| | | | |
|---|---|---|---|
| Formate oxidase | PCDDB/CD0005954000 | Ord | 185 |
| Calexcitin | PCDDB/CD0004676000 | Ord | 180 |
| Membrane-associated guanylate kinase-1, PDZ1 domain | PCDDB/CD0005960000 | Ord | 185 |
| Actin | PCDDB/CD0006122000 | Ord | 175 |
| Ion transport protein, full length, I218C mutant | PCDDB/CD0006141000 | Ord | 190 |
| Lasso peptide | PCDDB/CD0003930000 | Ord | 170 |
| Bovine collagen type II, 2.5 mg/ml | PCDDB/CD0004552000 | Ord | 170 |
| Poly-proline II, native | PCDDB/CD0004553000 | Ord | 173 |
| Bovine collagen type II, 1 mg/ml | PCDDB/CD0004554000 | Ord | 175 |
| Alpha1-antitrypsin | own measurement | Ord | 175 |
| Antithrombin | own measurement | Ord | 175 |
| Ecotin | own measurement | Ord | 175 |
| Human dUTPase | own measurement | Ord | 175 |
| 3-isopropylmalate dehydrogenase (*Thermus thermophilus*) | own measurement | Ord | 175 |
| K3 amyloid | own measurement | Ord | 175 |
| Abeta 1-42 amyloid | own measurement | Ord | 175 |
| Beta-2-microglobulin amyloid | own measurement | Ord | 175 |
| Alpha-2-macroglobulin | own measurement | Ord | 175 |
| Caskin-1, SH3 domain | own measurement | Ord | 175 |
| Flagellar secretion chaperone FliS | own measurement | Ord | 180 |
| Histone-lysine N-methyltransferase EZH2, wild type | own measurement | Dis | 171 |
| Histone-lysine N-methyltransferase EZH2, T-D mutant | own measurement | Dis | 171 |
| full scrambled ERD14 | own measurement | Dis | 175 |
| ERD full scrambled 100 residue seq. | own measurement | Dis | 175 |
| ERD ΔChp | own measurement | Dis | 175 |
| ERD ΔH | own measurement | Dis | 175 |
| ERD ΔKa | own measurement | Dis | 175 |
| ERD ΔKb | own measurement | Dis | 175 |
| ERD ΔKc | own measurement | Dis | 175 |
| Cellular tumor antigen p53, TAD | own measurement | Dis | 175 |
| Cellular tumor antigen p53, TAD mutant | own measurement | Dis | 175 |
| Disordered peptide 1[b] | own measurement | Dis | 177 |
| Disordered peptide 2[b] | own measurement | Dis | 179 |
| Disordered peptide 3[b] | own measurement | Dis | 175 |
| Disordered peptide 4[b] | own measurement | Dis | 175 |
| Disordered peptide 5[b] | own measurement | Dis | 176 |

| | | | |
|---|---|---|---|
| Disordered peptide 6[b] | own measurement | Dis | 177 |
| Disordered peptide 7[b] | own measurement | Dis | 176 |
| Artifical disordered protein containing Tumor necrosis factor receptor superfamily member 5 interaction domain | own measurement | Dis | 175 |
| Artifical disordered protein containing Tumor necrosis factor receptor superfamily member 5 interaction domain PG→AA mutant | own measurement | Dis | 175 |
| Artifical disordered protein containing Tumor necrosis factor receptor superfamily member 5 interaction domain SNT →AAA mutant | own measurement | Dis | 175 |
| Artifical disordered protein containing Fibronectin-binding protein A motif | own measurement | Dis | 175 |
| Artificial disordered protein containing Fibronectin-binding protein A motif GGGQ→NAKA mutant | own measurement | Dis | 175 |
| Artificial disordered protein containing SF1 (Splicing factor 1) motif | own measurement | Dis | 175 |
| Artifical disordered protein containing RAF proto-oncogene serine/threonine-protein kinase motif | own measurement | Dis | 175 |
| Artifical disordered protein containing Mothers against decapentaplegic homolog 3 motif | own measurement | Dis | 175 |
| Amylin, amyloid | (1) | Ord | 198 |
| Bovine phophatidylinositol 3-kinase p85alpha subunit, SH3 domain | (2) | Ord | 190 |
| Bovine phophatidylinositol 3-kinase p85alpha subunit, SH3 domain, amyloid | (2) | Ord | 190 |
| Cytochrome $c_{552}$ (*Hydogenobacter thermophilus*) | (3) | Ord | 190 |
| Cytochrome $c_{552}$, amyloid (*Hydogenobacter thermophilus*) | (3) | Ord | 190 |
| Monellin | (4) | Ord | 198 |
| Monellin, amyloid | (4) | Ord | 198 |
| Immunoglobulin light chain, SMA domain | (5) | Ord | 200 |
| Immunoglobulin light chain, SMA domain, amyloid | (5) | Ord | 200 |
| Bovine alpha-lactalbumin | (6) | Ord | 190 |
| Bovine alpha-lactalbumin, amyloid | (6) | Ord | 190 |
| Phosphoglycerate-kinase | (7) | Ord | 180 |
| Phosphoglycerate-kinase, amyloid | (7) | Ord | 185 |
| Prothymosin-alpha, amyloid | (8) | Ord | 195 |
| Abeta, amyloid | (9) | Ord | 200 |

| | | | |
|---|---|---|---|
| Alpha-synuclein | (10) | Dis | 190 |
| Alpha-synuclein, amyloid | (10) | Ord | 190 |
| Thymosin-alpha1 | (11) | Dis | 195 |
| C-Jun oncoprotein, basic subdomain | (12) | Dis | 185 |
| Gene 5 protein of Pf1 bacteriophage, C terminus | (13) | Dis | 190 |
| Yes-associated protein, WWdomain, W17F mutant | (14) | Dis | 200 |
| Alpha-tubulin(404-451) | (15) | Dis | 185 |
| Beta-tubulin(394-445) | (15) | Dis | 190 |
| Pig calpastatin, domain I | (16) | Dis | 190 |
| HIV-1 integrase, N terminus | (17) | Dis | 185 |
| RNA polymerase II, C terminus | (18) | Dis | 180 |
| Bob1, N terminus | (19) | Dis | 180 |
| S21 protein from the 30S subunit of the *Escherichia coli* ribosome | (20) | Dis | 185 |
| Subtilisin BPN', prodomain | (21) | Dis | 200 |
| Retinal phosphodiesterase, gamma subunit | (22) | Dis | 195 |
| Vmw65 from herpes simplex virus type 1, C terminal transactivation domain | (23) | Dis | 195 |
| S19 protein from the 30S subunit of the *Escherichia coli* ribosome | (20) | Dis | 185 |
| Wheat germ $E_m$ protein | (24) | Dis | 195 |
| Human group II protamine | (25) | Dis | 183 |
| Apo-cytochrome c | (26) | Dis | 195 |
| Prothymosin-alpha | (27) | Dis | 190 |
| Prothymosin-alpha | (28) | Dis | 190 |
| Fibronectin binding domain B | (29) | Dis | 190 |
| 4E binding protein | (30) | Dis | 200 |
| NF-kappaB, p65 subunit, C terminus | (31) | Dis | 190 |
| Fibronectin binding domain A | (29) | Dis | 190 |
| Fibronectin binding domain D | (29) | Dis | 190 |
| NACP (non-Abeta component of Alzheimer’s disease amyloid plaque) | (32) | Dis | 190 |
| Desiccation related protein | (33) | Dis | 185 |
| Thyroid transcription factor, N terminal domain | (34) | Dis | 198 |
| Myelin basic protein | (35) | Dis | 190 |
| Low affinity nerve growth factor receptor, extracellular domain | (36) | Dis | 190 |
| ACT265 | (37) | Dis | 180 |
| Proline-rich glycoprotein | (38) | Dis | 195 |

| | | | |
|---|---|---|---|
| Drosophila GAGA factor, glutamine-rich domain | (39) | Dis | 198 |
| Chromogranin A | (40) | Dis | 200 |
| Microtubule associated protein 2 | (41) | Dis | 205 |
| Parathyroid hormone-related protein, N-terminal fragment | (42) | Dis | 195 |
| Naturally occurring peptide LL-37 | (43) | Dis | 185 |
| DNA polymerase I, substrate binding peptide I | (44) | Dis | 190 |
| Yeast transcription factor GCN4, DNA-binding domain | (45) | Dis | 200 |
| RNase HI, C terminus | (46) | Dis | 200 |
| Lysozyme 1-40/98-127 (*Gallus gallus*) | (47) | Dis | 195 |
| Heat-stable inhibitor of the cAMP-dependent protein kinase | (48) | Dis | 180 |
| S18 protein from the 30S subunit of the *Escherichia coli* ribosome | (20) | Dis | 185 |
| SMK toxin, beta subunit | (49) | Dis | 200 |
| Cdk-inhibitor p21 | (50) | Dis | 200 |
| Beta-dystroglycan, N terminus | (51) | Dis | 190 |
| Human papillomavirus 16, E7 protein | (52) | Dis | 185 |
| Vitamin D receptor, DNA binding domain | (53) | Dis | 190 |
| RNase P | (54) | Dis | 195 |
| S12 protein from the 30S subunit of the *Escherichia coli* ribosome | (20) | Dis | 185 |
| Steroidogenic acute regulatory protein, N-terminal domain | (55) | Dis | 190 |
| Staphylococcal nuclease fragment | (56) | Dis | 190 |
| Calsequestrin | (57) | Dis | 200 |
| *Staphylococcus aureus* cell surface protein SdrD | (58) | Dis | 200 |
| Lysozyme (*Homo sapiens*) | (59) | Ord | 200 |
| Transthyretin | (60) | Ord | 200 |
| Prion protein | (61) | Ord | 200 |
| Beta-lactoglobulin | (62) | Ord | 200 |
| Bovine alpha-lactalbumin | (63) | Ord | 190 |
| Human alpha-lactalbumin | (63) | Ord | 190 |
| Beta-lactamase | (63) | Ord | 190 |
| Ribonuclease | (63) | Ord | 190 |
| Retinol-binding protein | (63) | Ord | 190 |
| Carbonic anhydrase | (63) | Ord | 190 |
| Phosphoglycerate kinase | (63) | Ord | 190 |
| Cytochrome c | (63) | Ord | 190 |

| | | | |
|---|---|---|---|
| Apomyoglobin | (63) | Ord | 190 |
| Leptin | (63) | Ord | 190 |

[a]CD spectra were either downloaded from PCDDB (64), measured by the authors or collected from the literature as referenced in the second column. Own measurements can be downloaded from https://bestsel.elte.hu/spectra_for_idp_classification.php. [b]Disordered peptide 1-7 are artificially designed 25 residue-peptides with high disorder estimated by various methods. Their sequences are as follows:

Disordered peptide 1: KTGKLPEETSAFEKSNESIVGETKE

Disordered peptide 2: DKMEESPEPIVSKSKVEEQEEKKEK

Disordered peptide 3: STEALEVKPLPEEYASRVEPGPHAE

Disordered peptide 4: VFDGEKVRHGGEEPGFLPAHGKSSV

Disordered peptide 5: KTEKESAIEVRHETTEPELVEQKKL

Disordered peptide 6: KPPIHKPKEDLEDVEHKEGVVDKKT

Disordered peptide 7: LPVKDLSKIKSEKMKDSEAVPKAKS

**Supplementary Table 2.** Disorder-order classification using two wavelengths[a]

| | | | | Error (%) | | |
|---|---|---|---|---|---|---|
| **Cut-off (nm)** | **Algorithm** | **WL1** | **WL2** | **Ordered** | **Disordered** | **Global** |
| 175 | Tree Simple | 187 | 190 | 1.6 | 0 | 1.3 |
| | Tree Medium | 191 | 200 | 0.8 | 4.8 | 1.3 |
| | KNN Weighted | 178 | 188 | 2.3 | 0 | 2 |
| 180 | Tree Medium | 185 | 192 | 2.7 | 0 | 2.2 |
| | KNN Fine | 189 | 199 | 2 | 3.3 | 2.2 |
| | Tree Simple | 187 | 190 | 1.3 | 6.7 | 2.2 |
| 185 | Tree Simple | 185 | 191 | 2.6 | 2.4 | 2.6 |
| | Tree Medium | 185 | 192 | 2.6 | 2.4 | 2.6 |
| | KNN Fine | 188 | 192 | 3.3 | 2.4 | 3.1 |
| 190 | Discr Quadratic | 194 | 198 | 4.7 | 3.4 | 4.4 |
| | Tree Medium | 198 | 207 | 3.5 | 6.8 | 4.4 |
| | KNN Fine | 190 | 199 | 3.5 | 8.5 | 4.8 |
| 195 | Discr Quadratic | 195 | 198 | 5.3 | 2.9 | 4.6 |
| | Tree Medium | 196 | 204 | 5.3 | 2.9 | 4.6 |
| | KNN Cosine | 197 | 216 | 5.8 | 2.9 | 5 |
| 200 | Tree Medium | 211 | 215 | 5.5 | 5 | 5.4 |
| | SVM Linear | 204 | 215 | 7.7 | 2.5 | 6.1 |
| | KNN Weighted | 210 | 213 | 6.1 | 6.3 | 6.1 |
| 205 | Tree Medium | 211 | 215 | 5.5 | 4.9 | 5.3 |
| | KNN Weighted | 210 | 213 | 6.1 | 6.2 | 6.1 |
| | KNN Cosine | 205 | 213 | 7.2 | 4.9 | 6.5 |

[a]Algorithms showing the least errors using two wavelengths (WL1 and WL2, in nm) for classification as a function of the cut-off wavelength.

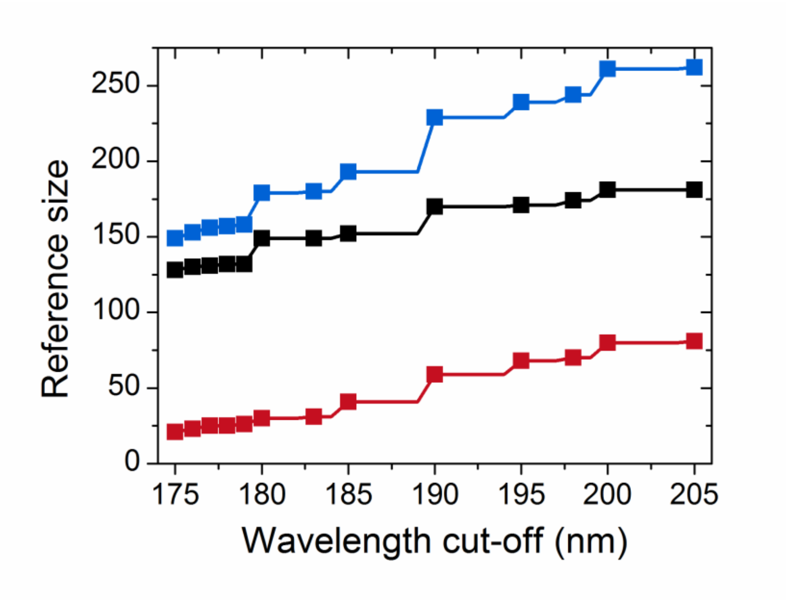


**Supplementary Figure 1.** Size of the reference data set as a function of the wavelength cut-off. Red: disordered proteins, black: ordered proteins, blue: overall reference size.

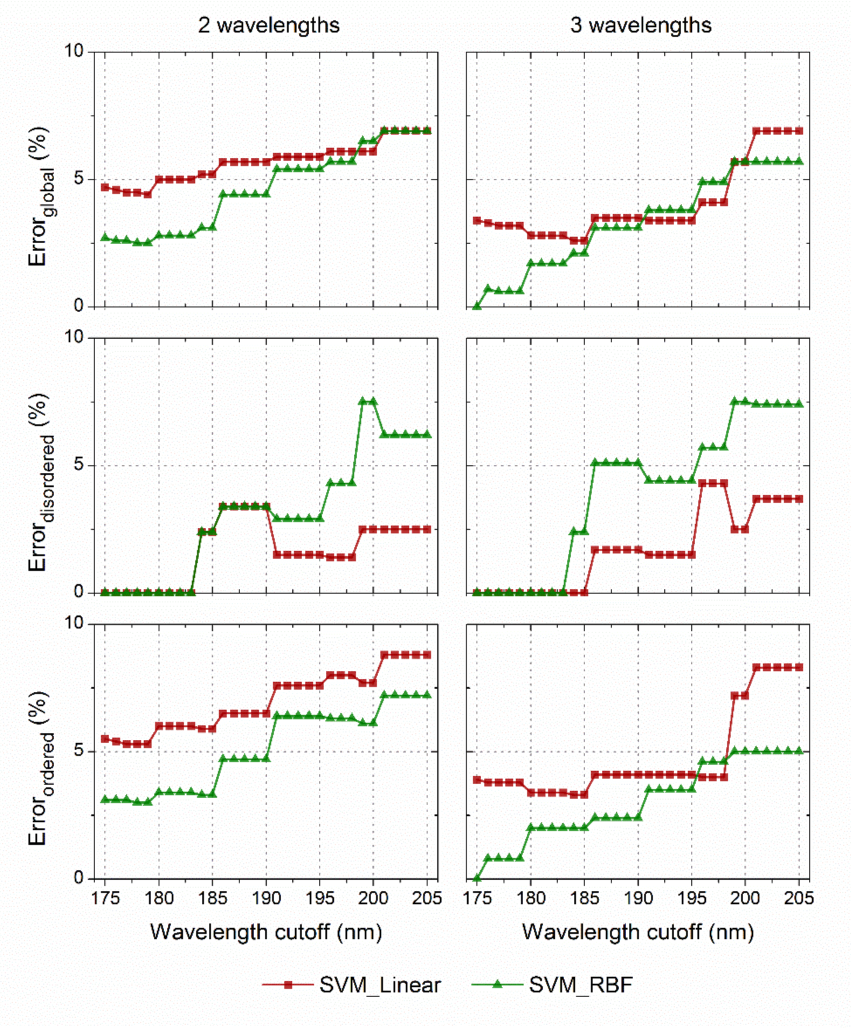


**Supplementary Figure 2.** Accuracy of the SVM-Linear and RBF algorithm as a function of the cut-off wavelength. Global error (top) and errors in classifying the disordered (middle) and ordered structures (bottom) for the two and three wavelength methods are shown.

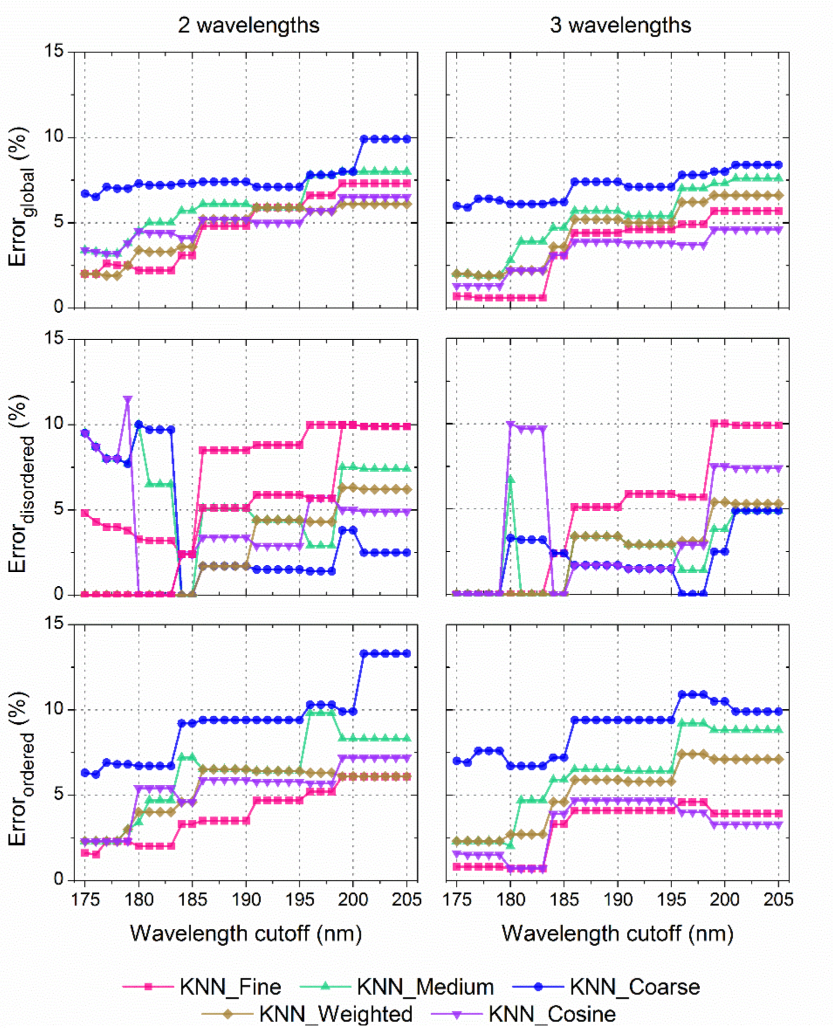


**Supplementary Figure 3.** Accuracy of the various KNN algorithms as a function of the cut-off wavelength. Global error (top) and errors in classifying the disordered (middle) and ordered structures (bottom) for the two and three wavelength methods are shown.

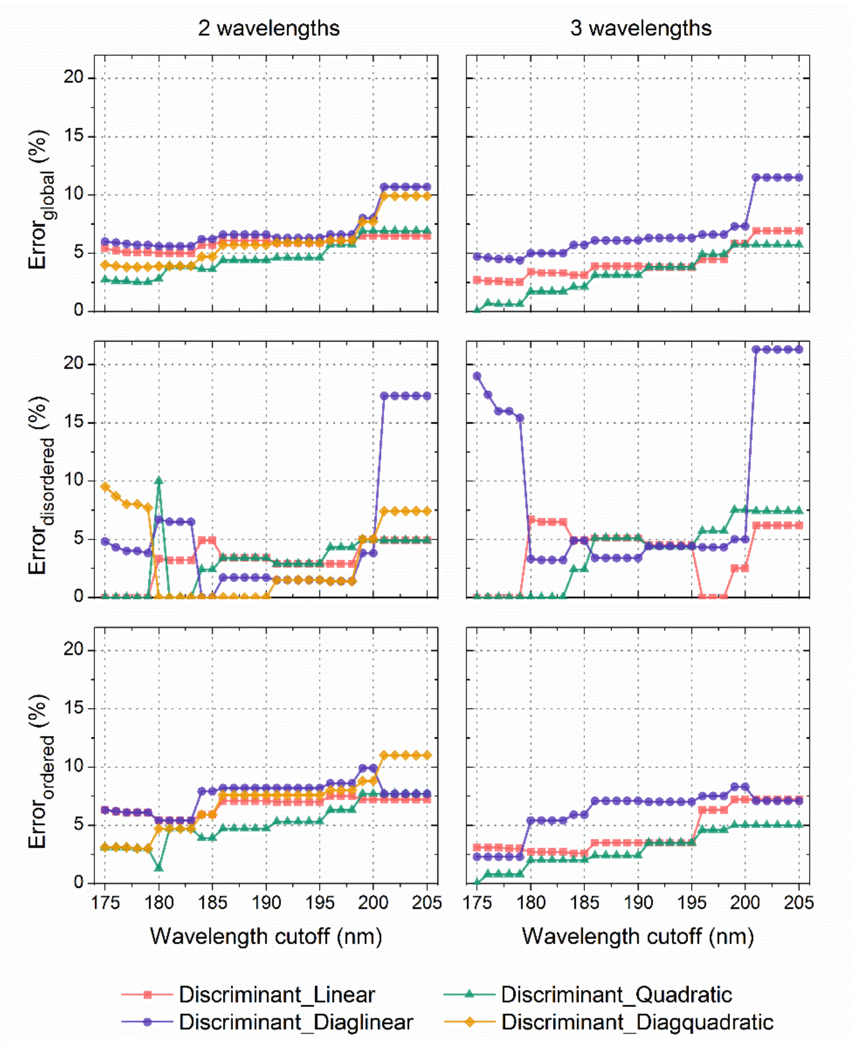


**Supplementary Figure 4.** Accuracy of the Discriminant-Linear, Quadratic, Diaglinear and Diagquadratic algorithms as a function of the cut-off wavelength. Global error (top) and errors in classifying the disordered (middle) and ordered structures (bottom) for the two and three wavelength methods are shown.

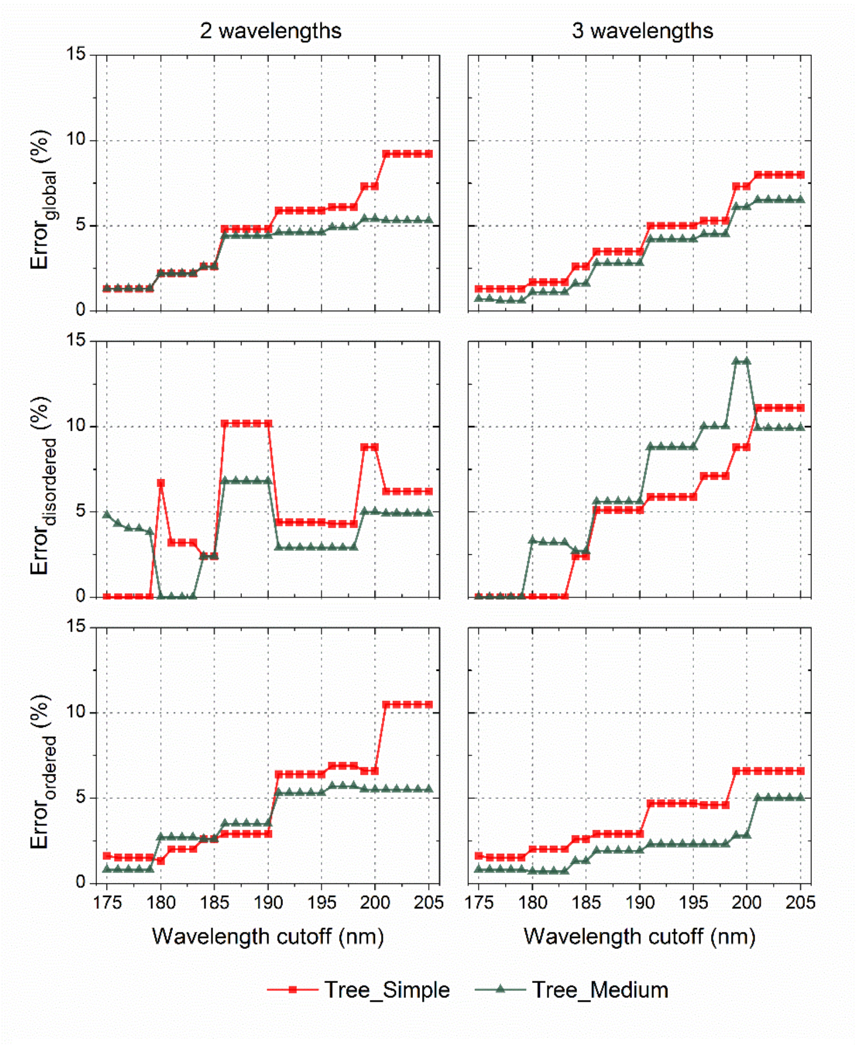


**Supplementary Figure 5.** Accuracy of Tree-Simple and Tree-Medium algorithms as a function of the cut-off wavelength. Global error (top) and errors in classifying the disordered (middle) and ordered structures (bottom) for the two and three wavelength methods are shown.

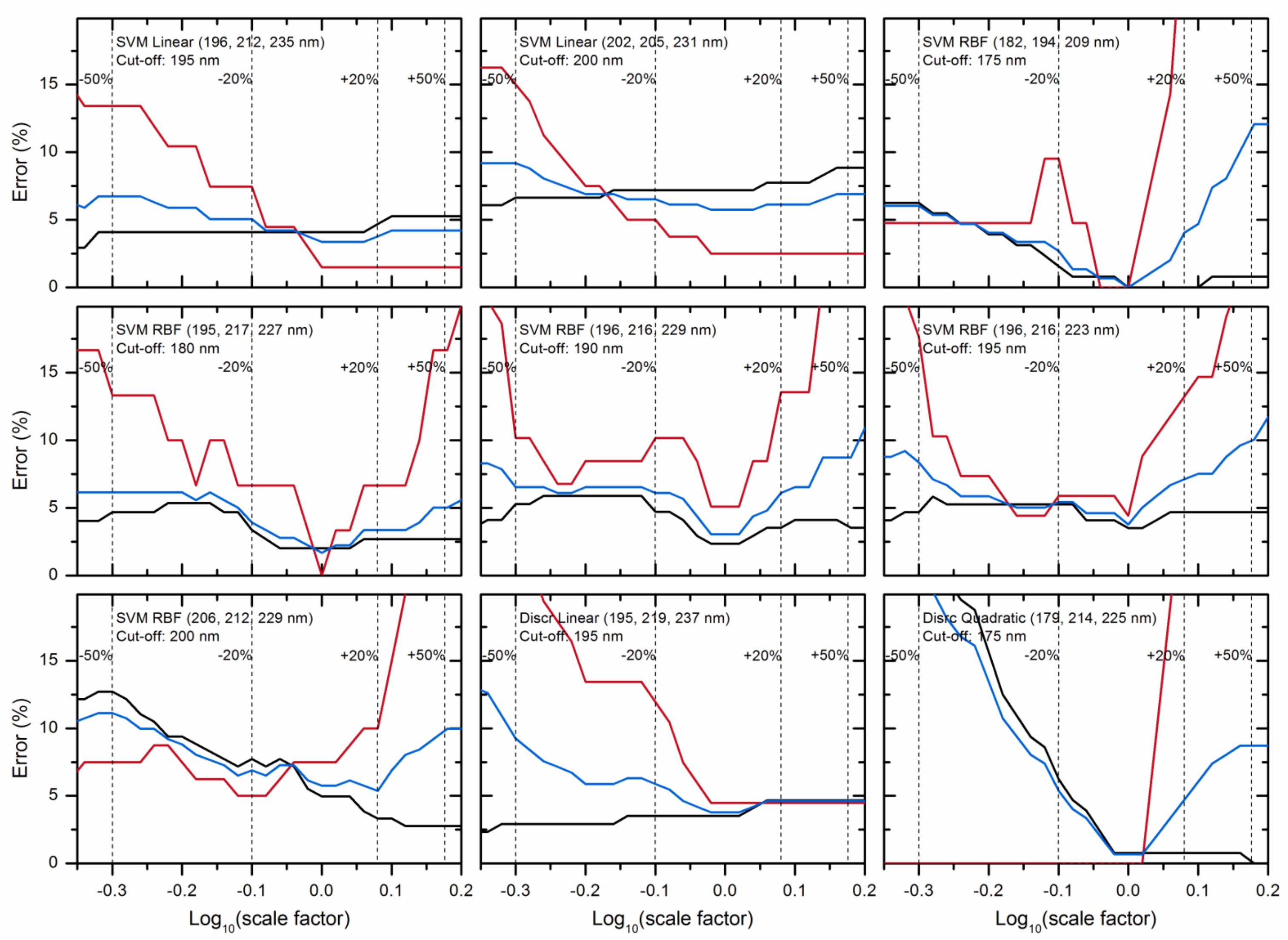


**Supplementary Figure 6,** continued on next page…

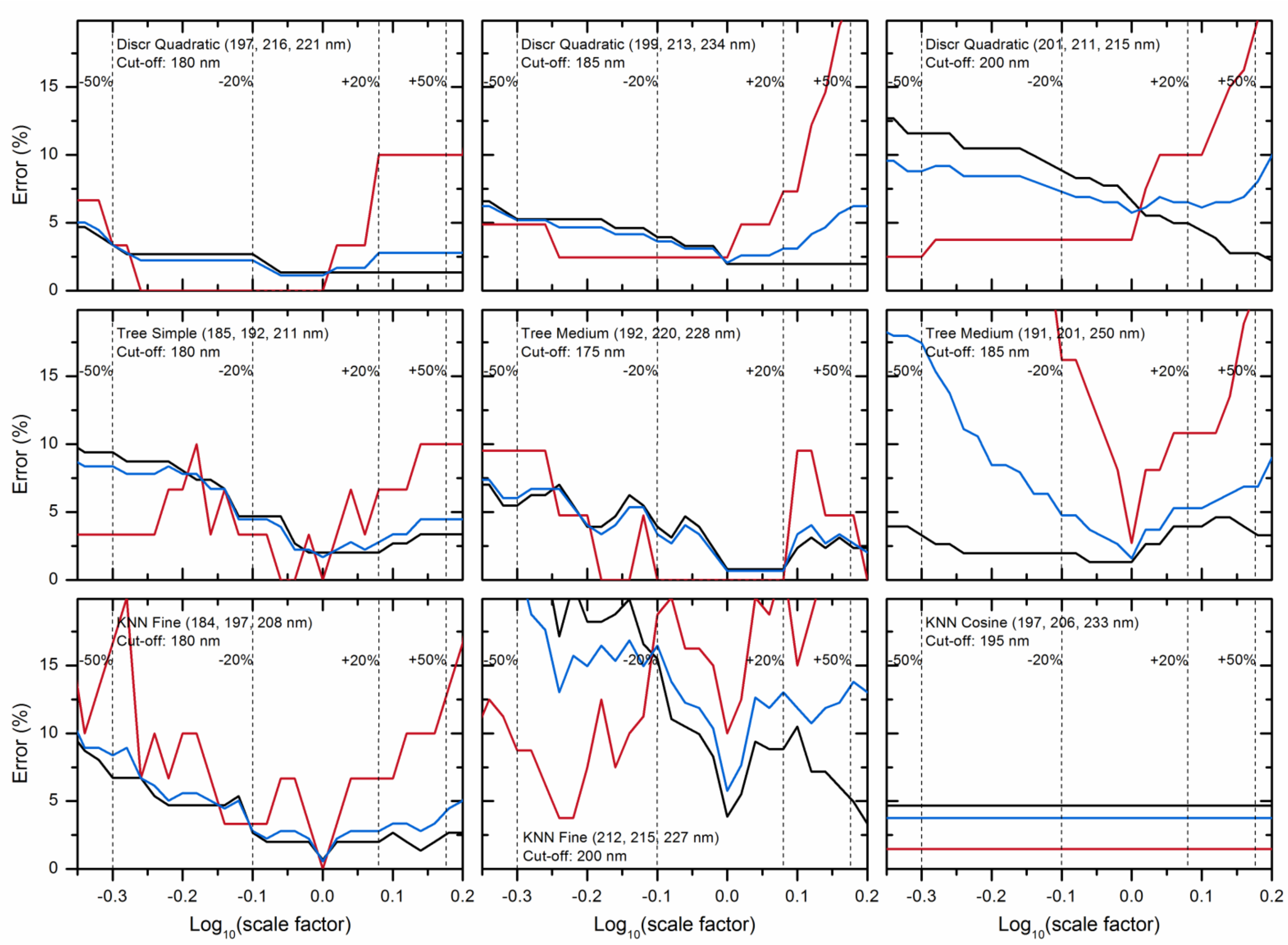


**Supplementary Figure 6.** Dependence of the accuracy of classification models on the concentration error. The spectra were re-scaled and the errors were calculated for all the algorithms with the corresponding wavelength triplets as presented in **Table 1.** Algorithm, cut-off, and wavelength values are shown on the panels. Please, note that KNN-Cosine has no concentration dependence.

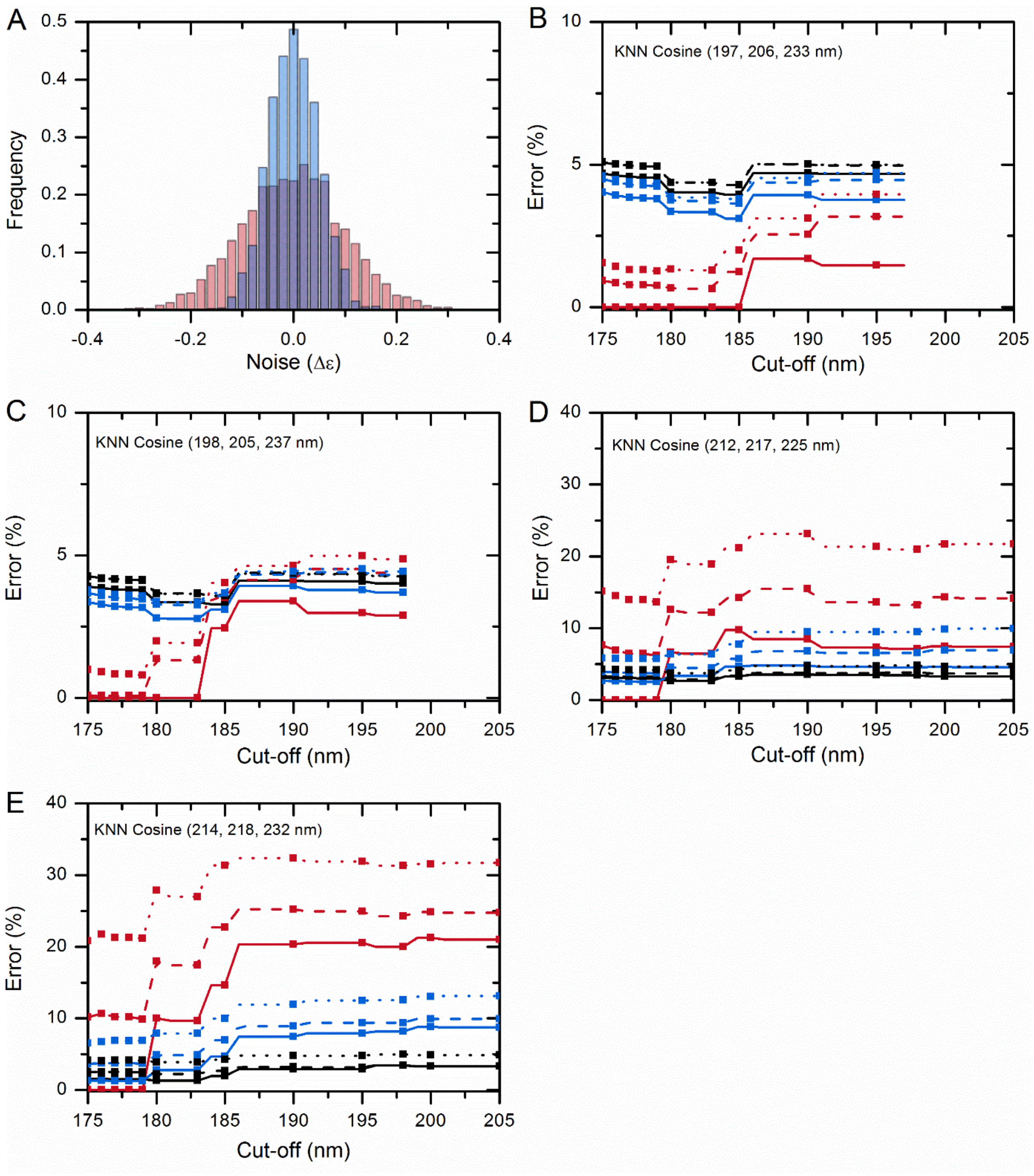


**Supplementary Figure 7.** The accuracy of KNN-Cosine algorithms and the effect of noise for various wavelength triplets. (A) Normal distribution noise-profiles applied on the spectra to test the effect of noise ($\mu$ = 0 $M^{-1}$ $cm^{-1}$, $\sigma$ = 0.05 $M^{-1}$ $cm^{-1}$ and 0.1 $M^{-1}$ $cm^{-1}$). (B-E) Disordered (red), ordered (black) and global (blue) errors are shown for the original spectra (solid line) and for spectra perturbed with the noise of $\sigma$ = 0.05 $M^{-1}$ $cm^{-1}$ (dashed line) and $\sigma$ = 0.1 $M^{-1}$ $cm^{-1}$ (dotted line). Wavelength triplets are indicated on the panels.

## Supplementary references


1. C. Goldsbury, K. Goldie, J. Pellaud, J. Seelig, P. Frey, S. Müller, J. Kistler, G. Cooper and U. Aebi: Amyloid fibril formation from full-length and fragments of amylin. *Journal of structural biology*, 130(2-3), 352-362 (2000)

2. J. I. Guijarro, M. Sunde, J. A. Jones, I. D. Campbell and C. M. Dobson: Amyloid fibril formation by an SH3 domain. *Proceedings of the National Academy of Sciences*, 95(8), 4224-4228 (1998)

3. T. A. Pertinhez, M. Bouchard, E. J. Tomlinson, R. Wain, S. J. Ferguson, C. M. Dobson and L. J. Smith: Amyloid fibril formation by a helical cytochrome. *FEBS letters*, 495(3), 184-186 (2001)

4. T. Konno, K. Murata and K. Nagayama: Amyloid-like aggregates of a plant protein: a case of a sweet-tasting protein, monellin. *FEBS letters*, 454(1-2), 122-126 (1999)

5. R. Khurana, J. R. Gillespie, A. Talapatra, L. J. Minert, C. Ionescu-Zanetti, I. Millett and A. L. Fink: Partially folded intermediates as critical precursors of light chain amyloid fibrils and amorphous aggregates. *Biochemistry*, 40(12), 3525-3535 (2001)

6. J. Goers, S. E. Permyakov, E. A. Permyakov, V. N. Uversky and A. L. Fink: Conformational prerequisites for α-lactalbumin fibrillation. *Biochemistry*, 41(41), 12546-12551 (2002)

7. G. Damaschun, H. Damaschun, K. Gast and D. Zirwer: Proteins can adopt totally different folded conformations. *Journal of molecular biology*, 291(3), 715-725 (1999)

8. N. A. Pavlov, D. I. Cherny, G. Heim, T. M. Jovin and V. Subramaniam: Amyloid fibrils from the mammalian protein prothymosin α. *FEBS letters*, 517(1-3), 37-40 (2002)

9. M. D. Kirkitadze, M. M. Condron and D. B. Teplow: Identification and characterization of key kinetic intermediates in amyloid β-protein fibrillogenesis. *Journal of molecular biology*, 312(5), 1103-1119 (2001)

10. V. N. Uversky, J. Li and A. L. Fink: Evidence for a partially folded intermediate in α-synuclein fibril formation. *Journal of Biological Chemistry*, 276(14), 10737-10744 (2001)

11. A. Grottesi, M. Sette, A. T. Palamara, G. Rotilio, E. Garaci and M. Paci: The conformation of peptide thymosin α1 in solution and in a membrane-like environment by circular dichroism and NMR spectroscopy. a possible model for its interaction with the lymphocyte membrane. *Peptides*, 19(10), 1731-1738 (1998)

12. D. Krebs, B. Dahmani, S. E. Antri, M. Monnot, O. Convert, O. Mauffret, F. Troalen and S. Fermandjian: The Basic Subdomain of the c-Jun Oncoprotein: A Joint CD, Fourier-Transform Infrared and NMR Study. *European journal of biochemistry*, 231(2), 370-380 (1995)

13. D. Fox, P. Cary and G. Kneale: Conformational studies of the C-terminal domain of bacteriophage Pf1 gene 5 protein. *Biochimica et Biophysica Acta (BBA)-Protein Structure and Molecular Enzymology*, 1435(1-2), 138-146 (1999)

14. E. K. Koepf, H. M. Petrassi, G. Ratnaswamy, M. E. Huff, M. Sudol and J. W. Kelly: Characterization of the structure and function of W→ F WW domain variants: identification of a natively unfolded protein that folds upon ligand binding. *Biochemistry*, 38(43), 14338-14351 (1999)

15. M. A. Jimenez, J. A. Evangelio, C. Aranda, A. Lopez-Brauet, D. Andreu, M. Rico, R. Lagos, J. M. Andreu and O. Monasterio: Helicity of α (404–451) and β (394–445) tubulin C-terminal recombinant peptides. *Protein Science*, 8(4), 788-799 (1999)

16. T. Konno, N. Tanaka, M. Kataoka, E. Takano and M. Maki: A circular dichroism study of preferential hydration and alcohol effects on a denatured protein, pig calpastatin domain I. *Biochimica et Biophysica Acta (BBA)-Protein Structure and Molecular Enzymology*, 1342(1), 73-82 (1997)

17. R. Zheng, T. M. Jenkins and R. Craigie: Zinc folds the N-terminal domain of HIV-1 integrase, promotes multimerization, and enhances catalytic activity. *Proceedings of the National Academy of Sciences*, 93(24), 13659-13664 (1996)

18. E. A. Bienkiewicz, A.-Y. M. Woody and R. W. Woody: Conformation of the RNA polymerase II C-terminal domain: circular dichroism of long and short fragments. *Journal of molecular biology*, 297(1), 119-133 (2000)

19. J.-F. Chang, K. Phillips, T. LundbaÈck, M. Gstaiger, J. E. Ladbury and B. Luisi: Oct-1 POU and octamer DNA co-operate to recognise the Bob-1 transcription co-activator via induced folding. *Journal of molecular biology*, 288(5), 941-952 (1999)

20. S. Y. Venyaminov and Z. V. Gogia: Optical characteristics of all individual proteins from the small subunit of Escherichia coli ribosomes. *European journal of biochemistry*, 126(2), 299-309 (1982)

21. M. A. Tangrea, P. Alexander, P. N. Bryan, E. Eisenstein, J. Toedt and J. Orban: Stability and global fold of the mouse prohormone convertase 1 pro-domain. *Biochemistry*, 40(18), 5488-5495 (2001)

22. V. N. Uversky, S. E. Permyakov, V. E. Zagranichny, I. L. Rodionov, A. L. Fink, A. M. Cherskaya, L. A. Wasserman and E. A. Permyakov: Effect of zinc and temperature on the conformation of the γ subunit of retinal phosphodiesterase: a natively unfolded protein. *Journal of proteome research*, 1(2), 149-159 (2002)

23. L. Donaldson and J. Capone: Purification and characterization of the carboxyl-terminal transactivation domain of Vmw65 from herpes simplex virus type 1. *Journal of Biological Chemistry*, 267(3), 1411-1414 (1992)

24. W. D. McCubbin, C. M. Kay and B. G. Lane: Hydrodynamic and optical properties of the wheat germ Em protein. *Canadian Journal of Biochemistry and Cell Biology*, 63(8), 803-811 (1985)

25. J. M. Gatewood, G. Schroth, C. Schmid and E. M. Bradbury: Zinc-induced secondary structure transitions in human sperm protamines. *Journal of Biological Chemistry*, 265(33), 20667-20672 (1990)

26. E. Stellwagen, R. Rysavy and G. Babul: The conformation of horse heart apocytochrome c. *Journal of Biological Chemistry*, 247(24), 8074-8077 (1972)

27. K. Gast, H. Damaschun, K. Eckert, K. Schulze-Forster, H. R. Maurer, M. Mueller-Frohne, D. Zirwer, J. Czarnecki and G. Damaschun: Prothymosin. alpha.: A biologically active protein with random coil conformation. *Biochemistry*, 34(40), 13211-13218 (1995)

28. V. N. Uversky, J. R. Gillespie, I. S. Millett, A. V. Khodyakova, A. M. Vasiliev, T. V. Chernovskaya, R. N. Vasilenko, G. D. Kozlovskaya, D. A. Dolgikh and A. L. Fink: Natively unfolded human prothymosin α adopts partially folded collapsed conformation at acidic pH. *Biochemistry*, 38(45), 15009-15016 (1999)

29. K. House-Pompeo, Y. Xu, D. Joh, P. Speziale and M. Höök: Conformational Changes in the Fibronectin Binding MSCRAMMs Are Induced by Ligand Binding (∗). *Journal of Biological Chemistry*, 271(3), 1379-1384 (1996)

30. C. M. Fletcher, A. M. McGuire, A.-C. Gingras, H. Li, H. Matsuo, N. Sonenberg and G. Wagner: 4E binding proteins inhibit the translation factor eIF4E without folded structure. *Biochemistry*, 37(1), 9-15 (1998)

31. M. L. Schmitz, M. dos Santos Silva, H. Altmann, M. Czisch, T. Holak and P. Baeuerle: Structural and functional analysis of the NF-kappa B p65 C terminus. An acidic and modular transactivation domain with the potential to adopt an alpha-helical conformation. *Journal of Biological Chemistry*, 269(41), 25613-25620 (1994)

32. P. H. Weinreb, W. Zhen, A. W. Poon, K. A. Conway and P. T. Lansbury: NACP, a protein implicated in Alzheimer's disease and learning, is natively unfolded. *Biochemistry*, 35(43), 13709-13715 (1996)

33. T. Lisse, D. Bartels, H. R. Kalbitzer and R. Jaenicke: The recombinant dehydrin-like desiccation stress protein from the resurrection plant Craterostigma plantagineum displays no defined three-dimensional structure in its native state (1996)

34. G. Tell, L. Perrone, D. Fabbro, L. Pellizzari, C. Pucillo, M. D. Felice, R. Acquaviva, S. Formisano and G. Damante: Structural and functional properties of the N transcriptional activation domain of thyroid transcription factor-1: similarities with the acidic activation domains. *Biochemical Journal*, 329(2), 395-403 (1998)

35. E. Polverini, A. Fasano, F. Zito, P. Riccio and P. Cavatorta: Conformation of bovine myelin basic protein purified with bound lipids. *European Biophysics Journal*, 28(4), 351-355 (1999)

36. D. E. Timm, P. Vissavajjhala, A. H. Ross and K. E. Neet: Spectroscopic and chemical studies of the interaction between nerve growth factor (NGF) and the extracellular domain of the low affinity NGF receptor. *Protein Science*, 1(8), 1023-1031 (1992)

37. J. P. Richards, H. P. Bächinger, R. H. Goodman and R. G. Brennan: Analysis of the structural properties of cAMP-responsive element-binding protein (CREB) and phosphorylated CREB. *Journal of Biological Chemistry*, 271(23), 13716-13723 (1996)

38. R. E. Loomis, E. J. Bergey, M. J. Levine and L. A. Tabak: Circular dichroism and fluorescence spectroscopic analyses of a proline-rich glycoprotein from human parotid saliva. *International journal of peptide and protein research*, 26(6), 621-629 (1985)

39. B. Agianian, K. Leonard, E. Bonte, H. Van der Zandt, P. B. Becker and P. A. Tucker: The glutamine-rich domain of the Drosophila GAGA factor is necessary for amyloid fibre formation in vitro, but not for chromatin remodelling. *Journal of molecular biology*, 285(2), 527-544 (1999)

40. S. H. Yoo and J. P. Albanesi: Ca2 (+)-induced conformational change and aggregation of chromogranin A. *Journal of Biological Chemistry*, 265(24), 14414-14421 (1990)

41. M. A. Hernández, J. Avila and J. M. Andreu: Physicochemical characterization of the heat-stable microtubule-associated protein MAP2. *European journal of biochemistry*, 154(1), 41-48 (1986)

42. K. Willis: Interaction with model membrane systems induces secondary structure in amino-terminal fragments of parathyroid hormone related protein. *International journal of peptide and protein research*, 43(1), 23-28 (1994)

43. J. Johansson, G. H. Gudmundsson, M. n. E. Rottenberg, K. D. Berndt and B. Agerberth: Conformation-dependent antibacterial activity of the naturally occurring human peptide LL-37. *Journal of Biological Chemistry*, 273(6), 3718-3724 (1998)

44. G. Mullen, J. Vaughn and A. Mildvan: Sequential proton NMR resonance assignments, circular dichroism, and structural properties of a 50-residue substrate-binding peptide from DNA polymerase I. *Archives of biochemistry and biophysics*, 301(1), 174-183 (1993)

45. M. A. Weiss, T. Ellenberger, C. R. Wobbe, J. P. Lee, S. C. Harrison and K. Struhl: Folding transition in the DMA-binding domain of GCN4 on specific binding to DNA. *Nature*, 347(6293), 575-578 (1990)

46. E. Kanaya and S. Kanaya: Reconstitution of Escherichia coli RNase HI from the N-fragment with High Helicity and the C-fragment with a Disordered Structure (∗). *Journal of Biological Chemistry*, 270(34), 19853-19860 (1995)

47. S. J. Demarest, S.-Q. Zhou, J. Robblee, R. Fairman, B. Chu and D. P. Raleigh: A comparative study of peptide models of the α-domain of α-lactalbumin, lysozyme, and α-lactalbumin/lysozyme chimeras allows the elucidation of critical factors that contribute to the ability to form stable partially folded states. *Biochemistry*, 40(7), 2138-2147 (2001)

48. J. Thomas, S. Van Patten, P. Howard, K. H. Day, R. D. Mitchell, T. Sosnick, J. Trewhella, D. Walsh and R. Maurer: Expression in Escherichia coli and characterization of the heat-stable inhibitor of the cAMP-dependent protein kinase. *Journal of Biological Chemistry*, 266(17), 10906-10911 (1991)

49. C. Suzuki, T. Kashiwagi, F. Tsuchiya, N. Kunishima, K. Morikawa, S. Nikkuni and Y. Arata: Circular dichroism analysis of the interaction between the alpha and beta subunits in a killer toxin produced by a halotolerant yeast, Pichia farinosa. *Protein engineering*, 10(2), 99-101 (1997)

50. R. W. Kriwacki, L. Hengst, L. Tennant, S. I. Reed and P. E. Wright: Structural studies of p21Waf1/Cip1/Sdi1 in the free and Cdk2-bound state: conformational disorder mediates binding diversity. *Proceedings of the National Academy of Sciences*, 93(21), 11504-11509 (1996)

51. E. Di Stasio, F. Sciandra, B. Maras, F. Di Tommaso, T. C. Petrucci, B. Giardina and A. Brancaccio: Structural and functional analysis of the N-terminal extracellular region of β-dystroglycan. *Biochemical and biophysical research communications*, 266(1), 274-278 (1999)

52. G. Pahel, A. Aulabaugh, S. A. Short, J. A. Barnes, G. R. Painter, P. Ray and W. Phelps: Structural and functional characterization of the HPV16 E7 protein expressed in bacteria. *Journal of Biological Chemistry*, 268(34), 26018-26025 (1993)

53. T. A. Craig, T. D. Veenstra, S. Naylor, A. J. Tomlinson, K. L. Johnson, S. Macura, N. Juranić and R. Kumar: Zinc binding properties of the DNA binding domain of the 1, 25-dihydroxyvitamin D3 receptor. *Biochemistry*, 36(34), 10482-10491 (1997)

54. C. H. Henkels, J. C. Kurz, C. A. Fierke and T. G. Oas: Linked folding and anion binding of the Bacillus subtilis ribonuclease P protein. *Biochemistry*, 40(9), 2777-2789 (2001)

55. M. Song, H. Shao, A. Mujeeb, T. L. James and W. L. Miller: Molten-globule structure and membrane binding of the N-terminal protease-resistant domain (63–193) of the steroidogenic acute regulatory protein (StAR). *Biochemical Journal*, 356(1), 151-158 (2001)

56. A. T. Alexandrescu, C. Abeygunawardana and D. Shortle: Structure and dynamics of a denatured 131-residue fragment of staphylococcal nuclease: a heteronuclear NMR study. *Biochemistry*, 33(5), 1063-1072 (1994)

57. B. Cozens and R. Reithmeier: Size and shape of rabbit skeletal muscle calsequestrin. *Journal of Biological Chemistry*, 259(10), 6248-6252 (1984)

58. E. Josefsson, D. O'Connell, T. J. Foster, I. Durussel and J. A. Cox: The binding of calcium to the B-repeat segment of SdrD, a cell surface protein of Staphylococcus aureus. *Journal of Biological Chemistry*, 273(47), 31145-31152 (1998)

59. L. A. Morozova-Roche, J. Zurdo, A. Spencer, W. Noppe, V. Receveur, D. B. Archer, M. Joniau and C. M. Dobson: Amyloid fibril formation and seeding by wild-type human lysozyme and its disease-related mutational variants. *Journal of structural biology*, 130(2-3), 339-351 (2000)

60. X. Jiang, C. S. Smith, H. M. Petrassi, P. Hammarström, J. T. White, J. C. Sacchettini and J. W. Kelly: An engineered transthyretin monomer that is nonamyloidogenic, unless it is partially denatured. *Biochemistry*, 40(38), 11442-11452 (2001)

61. P. Nandi, E. Leclerc, J.-C. Nicole and M. Takahashi: DNA-induced partial unfolding of prion protein leads to its polymerisation to amyloid. *Journal of molecular biology*, 322(1), 153-161 (2002)

62. K. Kuwajima, H. Yamaya and S. Sugai: The burst-phase intermediate in the refolding of β-lactoglobulin studied by stopped-flow circular dichroism and absorption spectroscopy. *Journal of molecular biology*, 264(4), 806-822 (1996)

63. K. S. Vassilenko and V. N. Uversky: Native-like secondary structure of molten globules. *Biochimica et Biophysica Acta (BBA)-Protein Structure and Molecular Enzymology*, 1594(1), 168-177 (2002)

64. L. Whitmore, A. J. Miles, L. Mavridis, R. W. Janes and B. A. Wallace: PCDDB: new developments at the Protein Circular Dichroism Data Bank. *Nucleic Acids Res*, 45(D1), D303-D307 (2017) doi:10.1093/nar/gkw796
